# Panchromatic PAH‐Porphyrin Hybrids with a Step‐Wise Increasing π‐System

**DOI:** 10.1002/open.202400481

**Published:** 2025-02-11

**Authors:** Christoph Oleszak, Christian L. Ritterhoff, Bernd Meyer, Norbert Jux

**Affiliations:** ^1^ Department of Chemistry and Pharmacyhsch & Interdisciplinary Center for Molecular Materials (ICMM) Chair of Organic Chemistry II Friedrich-Alexander-University Erlangen-Nürnberg Nikolaus-Fiebiger-Str. 10 91058 Erlangen Germany; ^2^ Interdisciplinary Center for Molecular Materials (ICMM) & Computer Chemistry Center (CCC) Friedrich-Alexander-University Erlangen-Nürnberg Nägelsbachstr. 25 91052 Erlangen Germany

**Keywords:** π-extension, Porphyrinoids, Fusion reaction, Scholl oxidation, Post-functionalization

## Abstract

The rational synthesis of three *β*‐*meso*‐fused porphyrins with a step‐wise increasing π‐system size is presented. The synthetic route, which introduces a five‐membered ring between the macrocycle and an aromatic fragment, is modular in its nature and proceeds straightforwardly. The well‐soluble conjugates have intriguing optical properties, namely bathochromically shifted and flattened absorption curves. Density functional theory (DFT) calculations provide insights into the electronic structure and transitions, unveiling small HOMO‐LUMO gaps.

## Graphical abstract

The synthesis of three, step‐wise in size increasing, *β*‐*meso*‐fused porphyrin conjugates is presented. The molecules exhibit exotic absorption and electronic features, originating from the five‐ring created between the porphyrin and an aromatic hydrocarbon fragment.

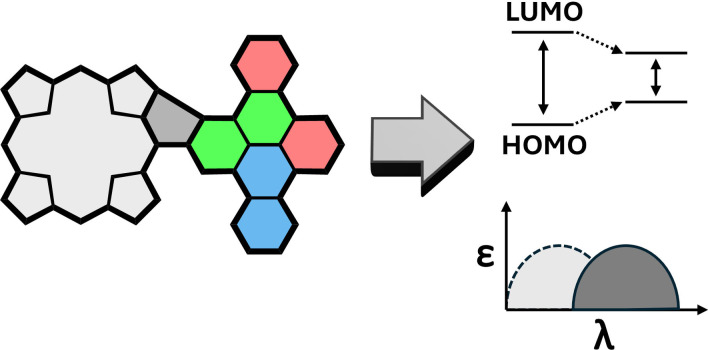

## Introduction

Not many molecules combine the features of having a standout role in nature while staying prevalent in a plethora of modern research fields for over a century. Porphyrins and their related derivatives, however, manage to meet both criteria and, as a result, draw great scientific attention to this day.[^1^, ^2^, ^3^] This is primarily due to their extraordinaire stability, modifiability, and their rich photophysical and electronic properties.^[4]^ While the pristine 18π‐electron macrocycle itself is of high scientific interest, many modern synthetic efforts also utilize it as a starting point for post‐functionalization cascades that aim at both an expansion and extension of its π‐system.[^5^, ^6^, ^7^, ^8^, ^9^] Enabled by the manifold pathways towards peripheral modification developed over the course of the decades, these enlargements of the aromatic system strongly alter and enhance the properties of porphyrins. More specifically, significant bathochromic absorption shifts and concomitant shrinkage in the energy gap are achieved, which render the resulting architectures highly interesting for applications in the fields of, *e.g*, near‐infrared (NIR) dyes, field‐effect transistors, and non‐linear optical (NLO) materials.[^10^, ^11^, ^12^, ^13^, ^14^, ^15^]

Several sub‐types and strategies for attaining the extension of porphyrins by fusion to aromatic fragments were established in the literature. These include the prominent *β*‐*β*‐fusion,[^16^, ^17^, ^18^] yielding extended benzo‐porphyrin‐type structures and the *β*‐*meso*‐fusion approach, which leads to tape‐like species.[^9^, ^19^, ^20^] While the latter method usually creates the most pronounced changes in the photophysical properties, it also often becomes more synthetically demanding with the increasing size of the attached fragment.[^21^, ^22^] This is well demonstrated by comparing the reports on naphthalene,[^23^, ^24^, ^25^] anthracene,[^9^, ^26^, ^27^, ^28^] and pyrene[^29^, ^30^] fused conjugates to the elaborate porphyrin‐nanoribbon structures recently presented by the groups of Müllen, Narita, Anderson, and Bogani.[^8^, ^31^, ^32^] However, the synthetic efforts in these examples are primarily associated with the formation of the final precursor, as the planarization step is, in most cases, a straightforward inter‐ or intramolecular oxidative cyclodehydrogenation. In solution, this aromatic coupling mostly relies on Scholl conditions involving either a mixture of FeCl_3_/CH_3_NO_2_ or DDQ/TfOH, although in some cases, more exotic combinations of Lewis acid and oxidant are needed.^[10]^ The most common fusion pattern resulting from these methods consist of either one or two six‐membered rings, connecting the two aromatic building blocks in a planar fashion. Especially when unsubstituted hydrocarbons are employed, this can lead to solubility issues due to enhanced π‐π stacking interactions between the target architectures. Therefore, several reports on molecules of this type make use of thermal fusion under vacuum conditions, as shown by Thompson and co‐workers, or on‐surface synthesis techniques,[^33^, ^34^] *e. g*., displayed by the group of Auwärter.^[35]^

**Figure 1:**
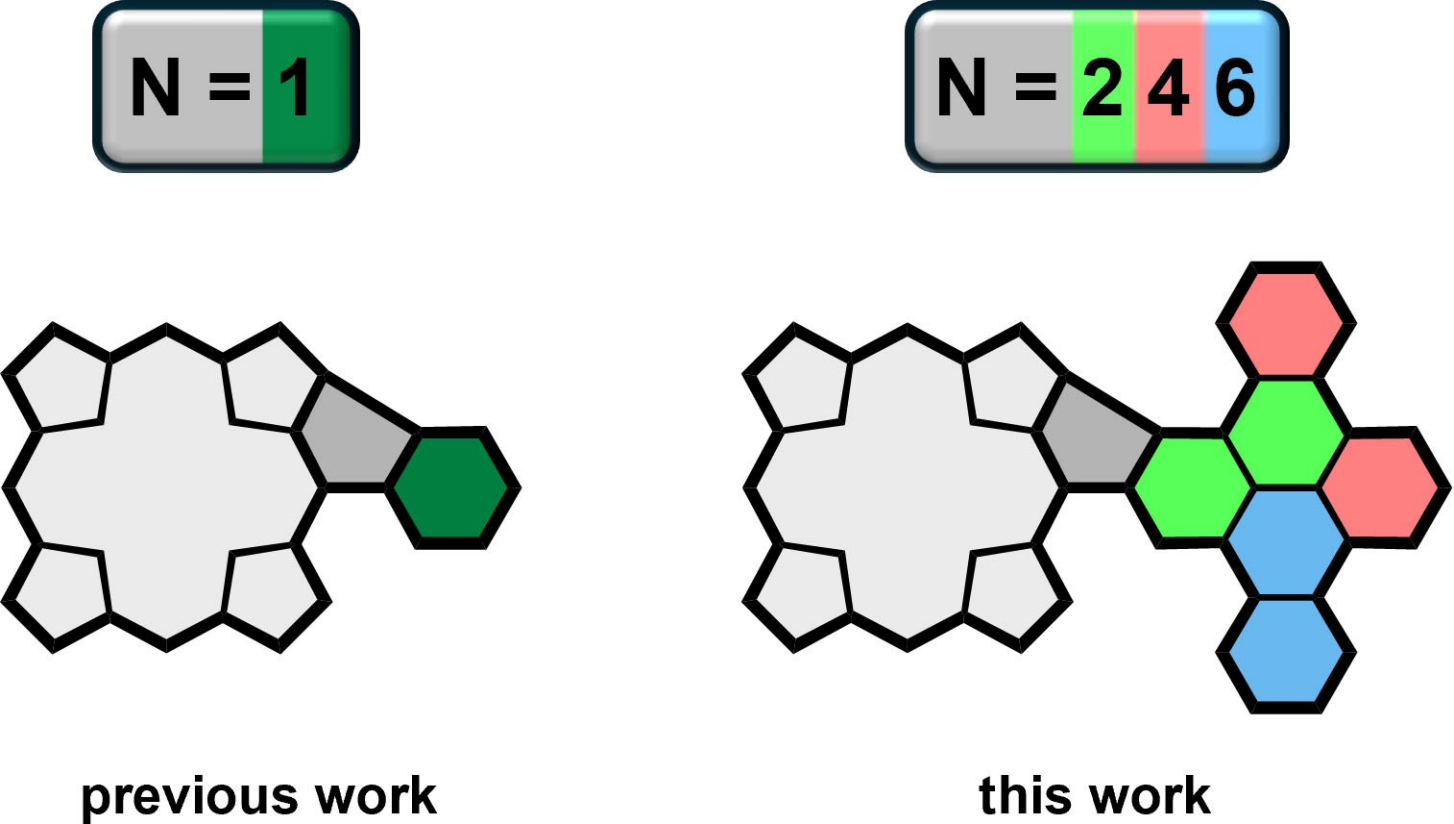
Visualization of this paper's concept of porphyrins fused to PAHs via *β*‐*meso* five‐membered rings.

A much less explored branch of porphyrin *β*‐*meso*‐fusion is one forming five‐ instead of six‐membered rings between the two aromatics.[^13^, ^36^, ^37^] Interestingly, the same motif is very much in vogue in modern nanographene chemistry because it induces negative curvature into the molecules, leading to bent structures with fascinating properties.[^38^, ^39^, ^40^] Highlighted in this context should be the efforts by Osuka and co‐workers on the fusion of multiple 3,5‐di‐*tert*‐butylphenyl groups, providing early examples of oxidatively fused conjugates.[^20^, ^41^] More recently, we followed up our own initial investigations on similarly phenyl‐fused A_4_‐porphyrins with the synthesis and thorough photophysical characterization of five‐ring‐containing molecules that included hexa‐*peri*‐hexabenzocoronene (HBC) building blocks.[^42^, ^43^, ^44^] Not only does the specific type of fusion greatly influence the geometry of the molecules by closing the space between the porphyrin and the HBC periphery, but it also significantly alters the electronic structure of the macrocycle by inducing anti‐aromatic and biradicaloid features.[^42^, ^44^] Herein, we now build on our recent findings by further extending the scope of five‐ring‐fused porphyrin‐polycyclic aromatic hydrocarbon (PAH) hybrids (Figure 1). To this end, three *β*‐*meso* fused A_3_B‐porphyrins coupled to in equal steps increasing substituents, namely naphthalene, triphenylene, and dibenzo[*fg*,*op*]tetracene, were synthesized and investigated spectroscopically and with (time‐dependent) density functional theory (DFT) calculations.

## Results and Discussion

**Scheme 1:**
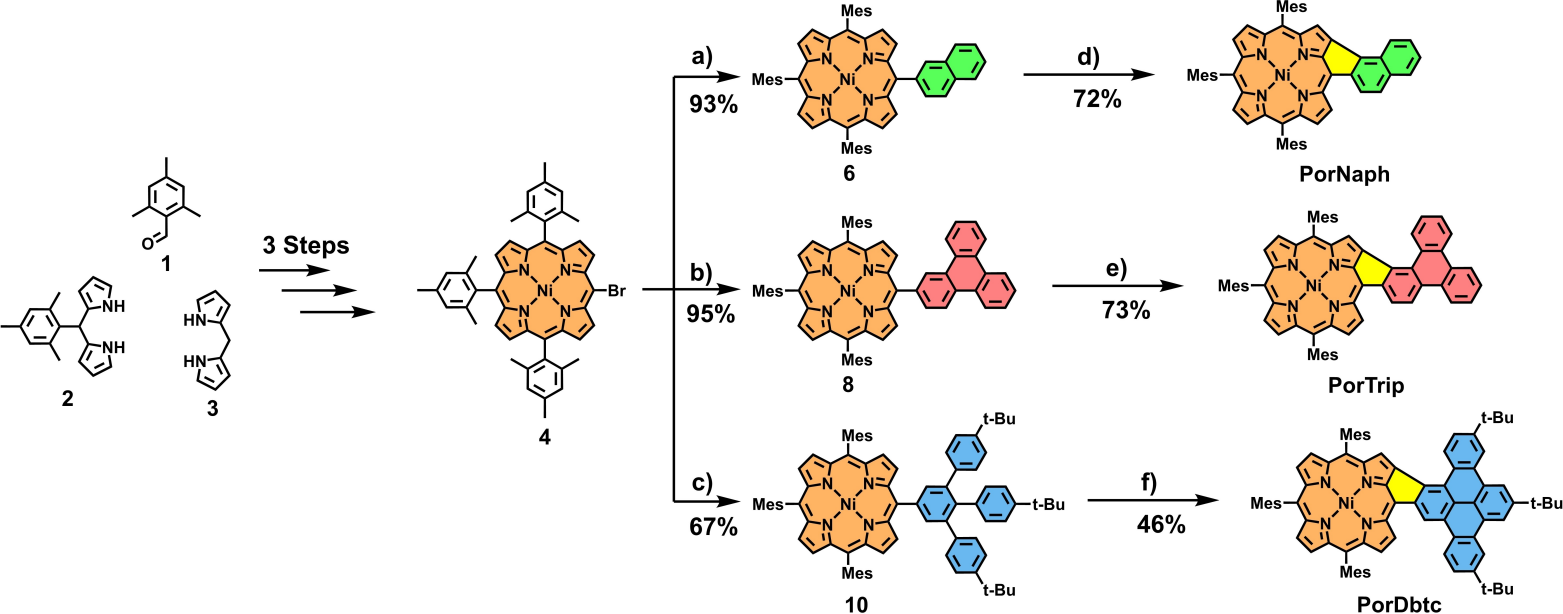
Synthesis of *β*‐*meso* Ni‐porphyrins **PorNaph**, **PorTrip**, and **PorDbtc**. Reagents and conditions: a–c) Pd(PPh_3_)_4_, Cs_2_CO_3_, toluene/DMF, 18 h, 80 °C, a: naphthyl‐boronic‐acid **5**, b: triphenylene‐boronic‐ester **7**, c: 3,4,5‐tri‐(4‐*tert*‐butyl‐phenyl)‐phenyl‐boronic‐ester **9**; d–f) FeCl_3_ (16 equiv), CH_3_NO_2_, CH_2_Cl_2_, 0 °C→rt, d: 3 h, e: 24 h, f: 48 h.

The synthetic strategy for obtaining the fused porphyrins is straightforward, relying on connecting a halogenated porphyrin with borylated PAH building blocks via Suzuki cross‐coupling reactions, followed by subsequent oxidative cyclodehydrogenation under Scholl conditions. In detail, initially, the brominated Ni‐trimesitylporphyrin **4** is prepared following procedures from the literature (for experimental details, see Scheme S1 in the Supporting Information). This porphyrin is designed with a prophylactical idea in mind. The peripheral mesityl groups protect three of the four *meso*‐sites from potential side reactions during the final oxidation step while at the same time rendering the porphyrin well‐soluble in common organic solvents. Furthermore, the porphyrin is metalated with a nickel ion. This is because the electronic structure of free‐base porphyrin doesn't allow for the *β*‐*meso* five‐ring fusion, as it has been proven on several different accounts in literature.[^20^, ^41^, ^43^, ^44^] Zink ions, on the other hand, do not withstand the relatively harsh Scholl conditions employed in our protocols.[^45^, ^46^, ^47^] Considering this, naphthyl‐porphyrin **6** was prepared by reacting **4** and 2‐naphthyl‐boronic‐acid **5** with Pd(PPh_3_)_4_ and Cs_2_CO_3_ in a mixture of toluene/DMF (ratio 2 : 1) at 80 °C for 18 h. Obtained in 93 % yield, the resulting porphyrin **6** was subjected to Scholl conditions. To this end, **6** was dissolved in CH_2_Cl_2_ and cooled to 0 °C, and 16 equivalents of FeCl_3_ in CH_3_NO_2_ were added to the mixture. The reaction was then stirred under slow warming to room temperature for 3 h until thin layer chromatography (TLC) analysis indicated complete conversion of the starting material. After a standardized work‐up, including quenching of the reaction with MeOH and NEt_3_ and filtration through silica, the fused species **PorNaph** was obtained in good yields of 72 % as a dark crystalline solid (Scheme 1). The Suzuki protocol from above was then employed to introduce two larger hydrocarbons into the *meso*‐position of **4**, namely, borylated triphenylene **7** and tailor‐made 3,4,5‐tri‐(4‐*tert*‐butyl‐phenyl)‐benzene **9**. The respective A_3_B conjugates **8** and **10** were obtained in 95 % and 67 % yield, respectively. This was followed up by subjecting the triphenylene conjugate **7** to the earlier established Scholl oxidation protocol, however, while increasing the reaction time to 24 h. After quenching and chromatographic work‐up, the fused species **PorTrip** was obtained in 73 % yield. Lastly, the largest conjugate, **10**, was reacted in the same way. Contrary to the two cyclodehydrogenation reactions laid out prior, in this step, three C−C bonds, as opposed to one, have to be formed.

**Scheme 2:**
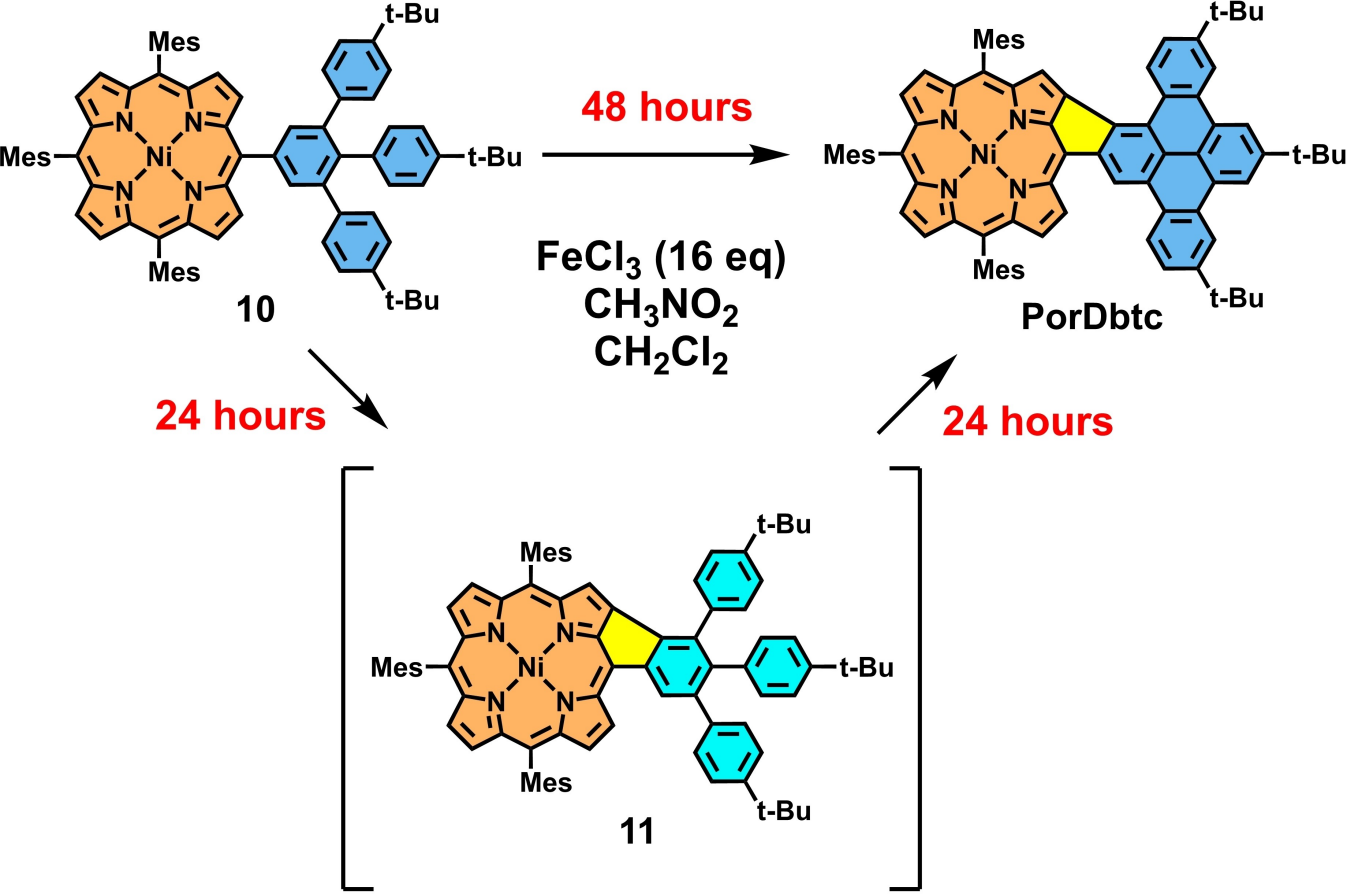
Transformation of **10** to **PorDbtc** via the intermediate **11**.

Hereby, it is noteworthy that the bond between the porphyrin and the phenyl group is closed much faster than the other two bonds. As a result, when stopping the reaction after 24 h, side product **11** can be isolated as a dark‐green solid in an excellent yield of 87 % (Scheme 2). **11** could then be subjected again to identical oxidation conditions, leading to complete planarization of the system within another 24 h. Nevertheless, fully fused congener **PorDbtc** was also obtainable in one single step with a yield of 46 % by extending the initial reaction time to 48 h. The solubility of the three fused conjugates remained excellent in common organic solvents like dichloromethane, and therefore, unambiguous characterization by NMR spectroscopy and mass spectrometry was possible.

**Figure 2:**
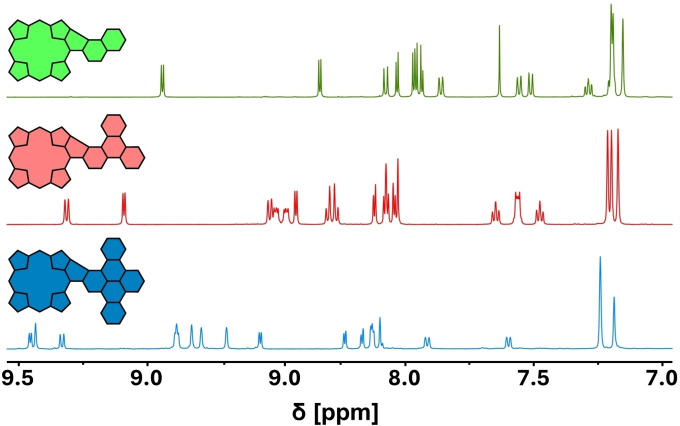
Aromatic region of the ^1^H NMR spectra of **PorNaph**, **PorTrip**, and **PorDbtc** (CD_2_Cl_2_, rt).

Comparing the ^1^H NMR Spectra of **PorNaph**, **PorTrip**, and **PorDbtc**, the loss of symmetry in comparison to the respective precursors becomes apparent (for spectra of the precursors, see Supporting Information). The signals of the aromatic hydrogens coupled to porphyrin and hydrocarbon are distributed across the region from 9.5–7.0 ppm and are partially overlapping (Figure 2). Nevertheless, all signals could be successfully assigned with the help of 2D NMR spectra (for comprehensive assignments, see Figures S32–34).

**Figure 3:**
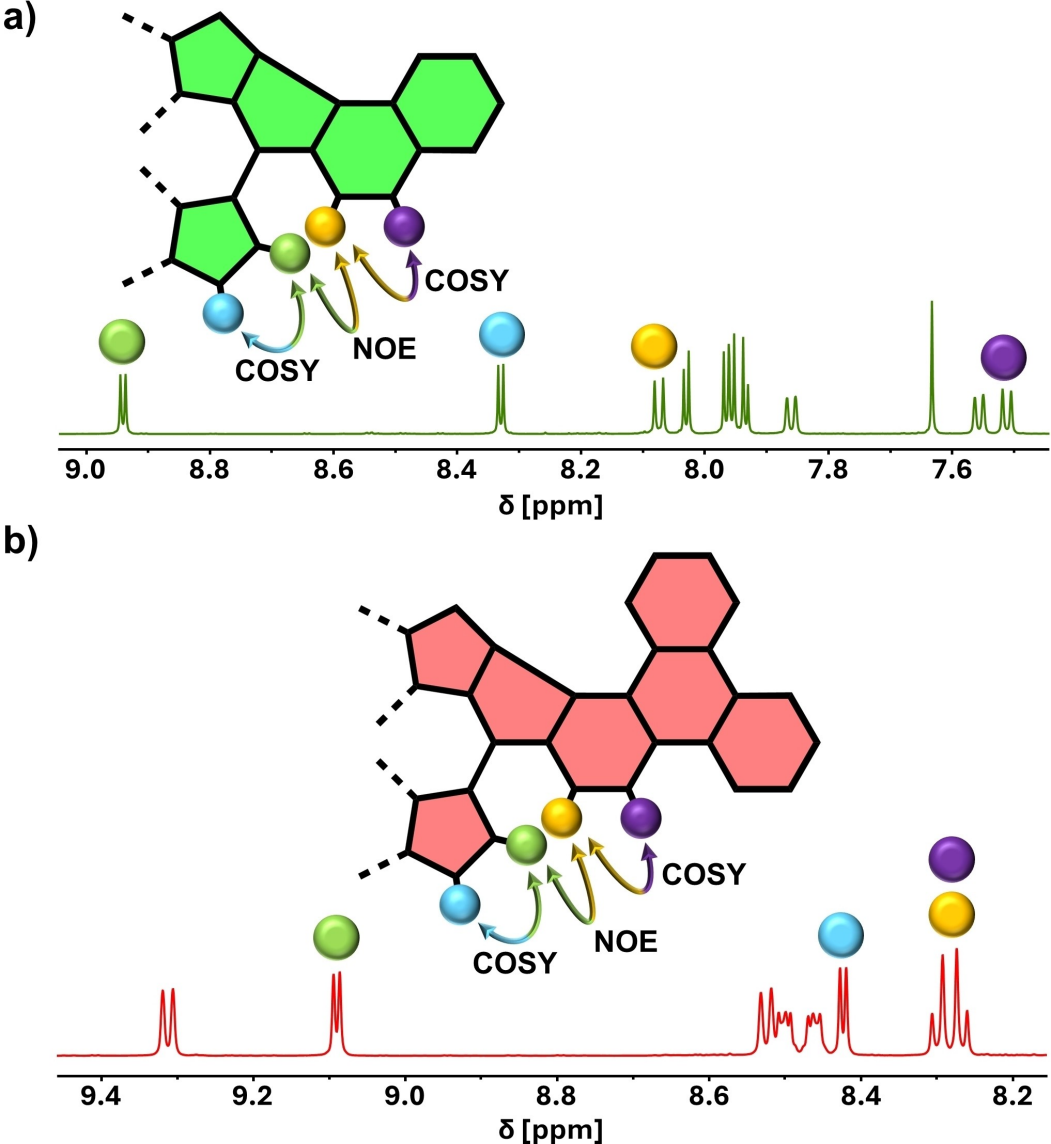
Assignment of the regiochemistry in a) **PorNaph** and b) **PorTrip** with the help of 2D NMR spectroscopy.

Additionally, in the case of **PorNaph** and **PorTrip**, a combination of selective nuclear Overhauser effect (NOE) and correlation spectroscopy (COSY) experiments unveiled the absolute position of the newly formed C−C bond (Figure 3). In both cases, the same set of highlighted hydrogens makes this assignment possible. In detail, a through‐space interaction between the *β*‐pyrrolic hydrogen atom (*green*) and naphthalene/triphenylene hydrogen atom (*yellow*) was detected. A doublet signal splitting, as well as a correlation signal in the COSY spectrum, indicated the immediate proximity of the two proton pairs (*green*/*blue* and *yellow*/*purple)*. If the bond were to be located on the opposite side, different splittings and correlations would be expected caused by the altered symmetry of the fused fragment. The most plausible explanation for the sole formation of one respective isomer is derived from the mechanism of the fusion reaction. According to reports from recent literature, this mechanism is proceeding via the formation of two radical‐cation species (one radical being located on the porphyrin and one on the *meso*‐bound aromatic fragment), which are subsequently connected by an intramolecular radical‐radical coupling reaction.[^30^, ^48^] As a result, the regioselectivity of the reaction would be governed by the spin densities in the molecules. In our case, the spin density values appear to be sufficiently different, so the coupling occurs in the 1‐position in both triphenylene and naphthalene selectively.^[20]^

**Figure 4:**
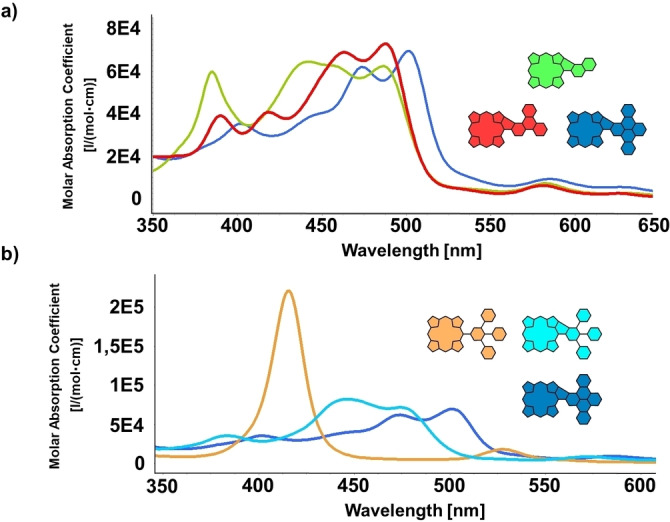
a) UV/Vis absorptions of **PorNaph**, **PorTrip**, and **PorDbtc** (CH_2_Cl_2_); b) Comparison of **10**, **11**, and **PorDbtc** (CH_2_Cl_2_).

To gain insights into the optical features of the fused conjugates, UV/Vis measurements were conducted. In Figure 4a, the spectra of **PorNaph**, **PorTrip**, and **PorDbtc** are shown stacked above each other. In general, a significant broadening and bathochromic shift of the absorptions, compared to ordinary nickel‐porphyrins, can be observed (for reference, the absorption spectrum of precursor **10** in Figure 4b can be considered). The former characteristic, distinct Soret‐ and Q‐bands disappeared, and the curves consist of an almost panchromatic, single, strong band with multiple local maxima, which dominates the visible part of the spectrum and minor absorptions in the NIR area. To no surprise, a more detailed cross‐comparison reveals that the red‐shift of absorption becomes more pronounced with an increase of the π‐system. This is in line with the general trend observed in our previous works and the literature.[^21^, ^22^, ^29^, ^42^, ^43^, ^44^] However, the specific shapes of the individual bands differ notably from each other. Standing out is hereby a strong local maximum at 385 nm as well as an increased absorption in the area of 400–450 nm for **PorNaph**. In the latter‐mentioned spectral sector, the global maximum is located at 442 nm, while the absorption intensity remains stationary up until 486 nm. For the two larger congeners, **PorTrip** and **PorDbtc**, the curves not only cover less area in this specific region but also appear shape‐wise more alike. However, **PorDbtc** absorbs significantly more light in the range from 500–550 nm, with the global maximum at 501 nm. On the other hand, the band of **PorTrip** shows the highest absolute extinction coefficient of the group at 488 nm (Figure 4a and Table 1).

**Table 1:** UV/Vis absorption data of **PorNaph**, **PorTrip**, **PorDbtc**, and **11** in CH_2_Cl_2_. The four most intense absorptions for each conjugate are listed, with the global maximum being highlighted in color.

	Abs. I	Abs. II	Abs. III	Abs. IV
**PorNaph** **ϵ** **[l/(mol⋅cm)]**	385 nm (60000)	**442 nm** **(65000)**	486 nm (62500)	582 nm (8000)
**PorTrip** **ϵ** **[l/(mol⋅cm)]**	418 nm (41000)	463 nm (69000)	**488 nm** **(73000)**	581 nm (6000)
**PorDbtc** **ϵ** **[l/(mol⋅cm)]**	402 nm (35000)	474 nm (62000)	**501 nm** **(70000)**	585 nm (9000)
**11** **ϵ** **[l/(mol⋅cm)]**	384 nm (36000)	**447 nm** **(82000)**	474 nm (71000)	573 nm (9000)

In Figure 4b, a closer look at the molecules participating in the in Scheme 2 displayed transformation of **11** to **PorDbtc** was taken. As mentioned earlier, the features of both fused species differ vastly from those of the starting material. However, the changes going from **11** to **PorDbtc** are also significant, with the global absorption maxima being separated by more than 50 nm. These differences become very apparent when comparing the macroscopic appearance of the three molecules, with **10** exhibiting a vibrant orange color, while **11** possessing a deep dark‐green and **PorDbtc** a dark‐brown color. As expected, the spectral shape of **11** is also much more reminiscent of **PorNaph** than **PorDbtc** and **PorTrip**. This can be attributed to **11** representing a type of phenyl‐fused molecules that we had also encountered in our earlier works, which we assume are electronically more similar to **PorNaph** than to the other two conjugates.^[43]^

**Figure 5:**
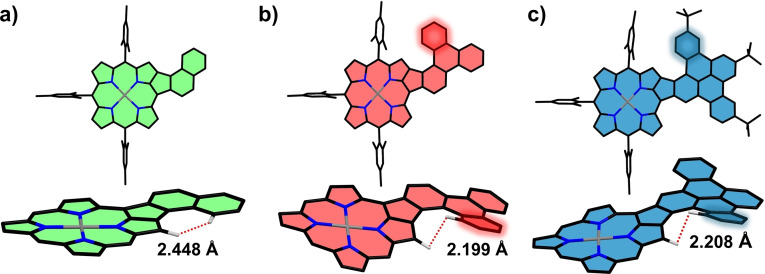
Optimized geometries of a) **PorNaph**, b) **PorTrip**, and c) **PorDbtc**. For each molecule, a front (*top*) and side view (*bottom*) are shown. In the side view, the two hydrogens that are closest to each other and the respective distances between them are indicated. For **PorTrip** and **PorDbtc**, the benzene ring responsible for the twisted structure is highlighted.

DFT calculations were performed to gain deeper insight into the atomic and electronic structure of the conjugates. The optimized three‐dimensional geometries of **PorNaph**, **PorTrip**, and **PorDbtc** are presented in Figure 5. When comparing the molecules, it is noteworthy that the highlighted benzene ring in **PorTrip** and **PorDbtc** induces a helical twist in these molecules, originating from steric repulsion between the two marked hydrogens (Figure 5b and c, *bottom*). The resulting motif is reminiscent of a [5]helicene, in which two of the six‐rings are replaced by five‐rings. However, at ambient conditions, no spectroscopic evidence for the existence of enantiomers was found. Therefore, we performed nudged elastic band (NEB) calculations to estimate the activation barrier for the conversion of the two possible enantiomers. The resulting energies, 0.2 eV and 0.3 eV for **PorTrip** and **PorDbtc**, respectively, explain the lack of experimental evidence, as this barrier is easily overcome at room temperature. As expected from the similar activation energy, the distance between the two repelling hydrogen atoms is almost equal in both fused congeners with a value of ~2.2 Å. This value is in line with the hydrogen distances in ordinary stand‐alone helicenes and slightly lower than the sum of the van‐der‐Waals radii of the individual hydrogen atoms.[^49^, ^50^] For **PorNaph**, on the other hand, the closest measured distance between two hydrogen atoms is 2.45 Å, which is too large to create a similar repulsion‐induced helicity. As a result, in contrast to **PorTrip** and **PorDbtc**, the structure of **PorNaph** is almost completely planar (Figure 5a).

**Figure 6:**
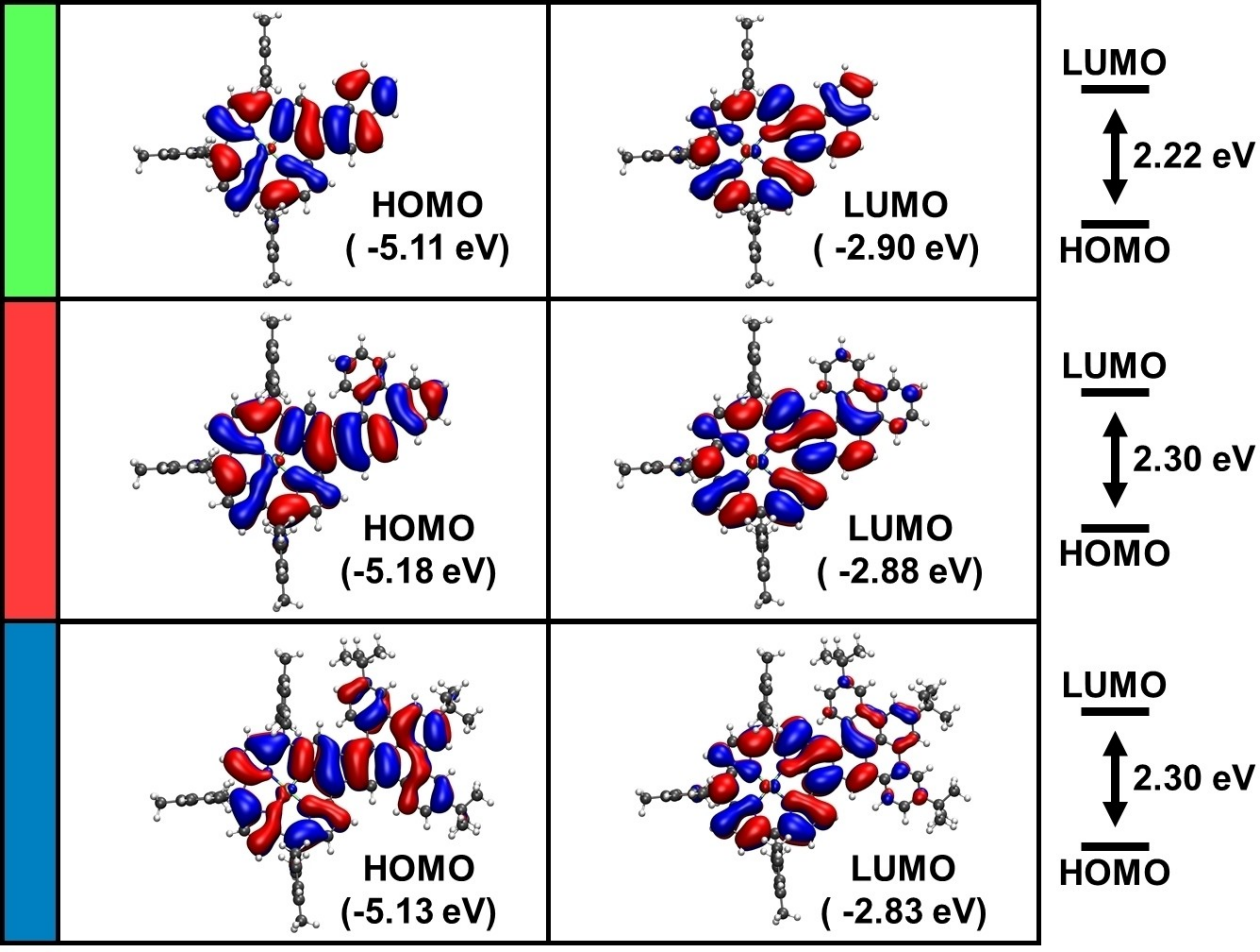
Orbital contours and eigenvalues of the HOMO and LUMO of **PorNaph**, **PorTrip**, and **PorDbtc** in the gas phase, calculated by DFT at the B3LYP/def2‐TZVPP level of theory including an implicit solvation model.

A closer look at the orbital energies of the molecules reveals some interesting electronic characteristics (Figure 6). In general, a significant decrease of around 0.7 eV in gap energy compared to the Ni‐tetramesitylporphyrin (**Por**) reference can be observed (see Figure S37 and Table S2 in the Supporting Information for orbital contours and energies of **Por**). Somewhat surprisingly, the smallest of the three molecules, **PorNaph**, shows the smallest HOMO‐LUMO gap (2.22 eV). Usually, one would expect that increasing the size of the π‐system leads to a reduction of the energy gap.[^21^, ^22^] However, the reversal of this trend is explained by the structural deformation of the two larger molecules. For the planar **PorNaph**, the HOMO and LUMO are an equal superposition of the frontier orbitals of their building blocks (see Figures S35‐S38). While this holds true for **PorTrip** and **PorDbtc** to some degree as well, the helical twist reduces the contribution of the orbitals of the fused moiety. Consequently, the frontier orbitals have a higher weight on the porphyrin and the fused five‐ring (see Figure 6). Therefore, the extension of the π‐system has a less pronounced effect, resulting in gap energies of 2.30 eV for both **PorTrip** and **PorDbtc**.

**Figure 7:**
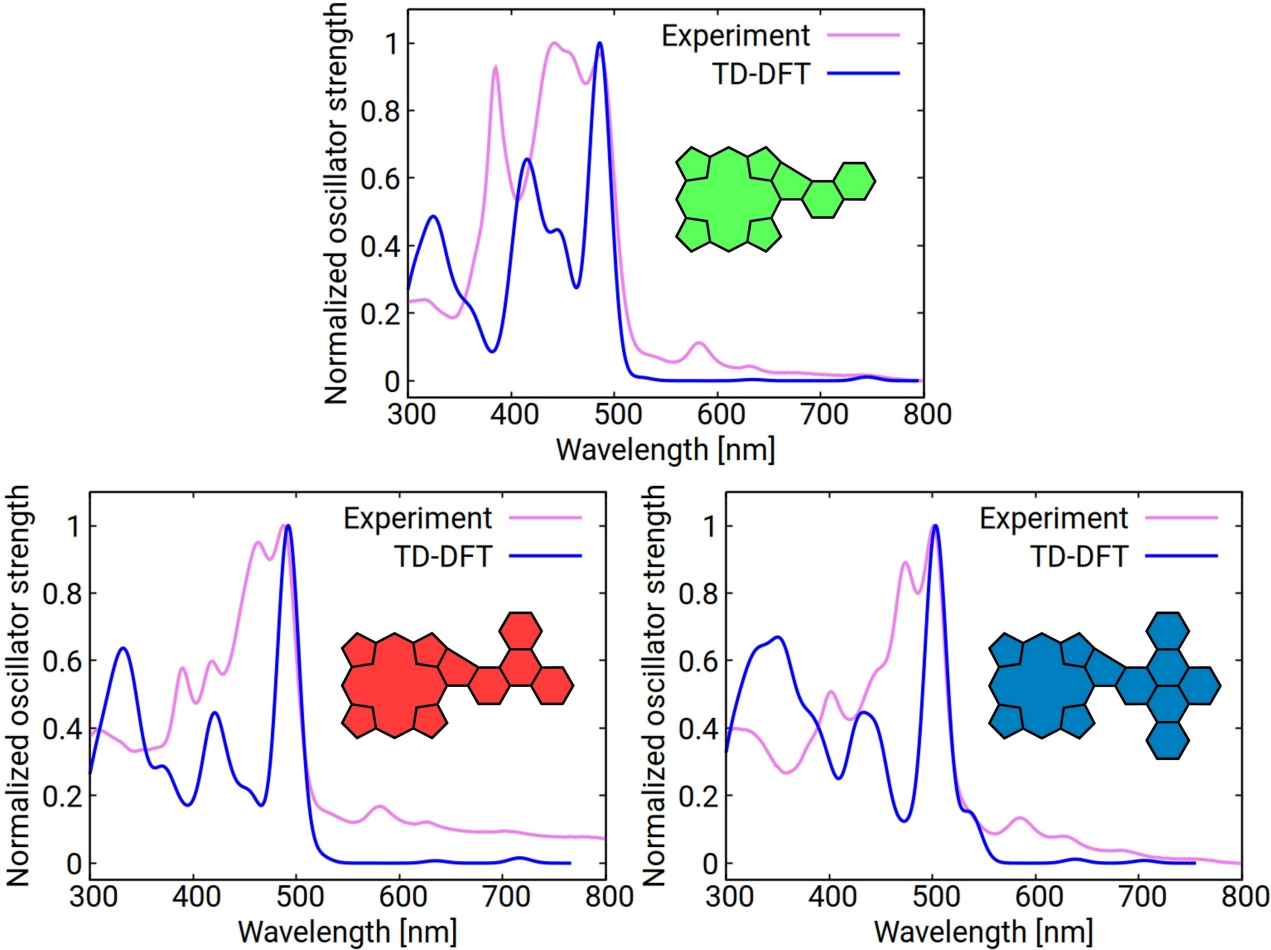
Calculated TD‐DFT absorption spectra of **PorNaph**, **PorTrip**, and **PorDbtc** (CH_2_Cl_2_). The calculated spectra were red‐shifted by 88 nm and broadened by a Gaussian function with a width of 12 nm to facilitate comparison.

Time‐dependent (TD)‐DFT calculations reveal a similar trend. The first excitation, consistently attributed to the HOMO/LUMO transition for all three molecules, shows the lowest energy in the case of **PorNaph** with 1.89 eV, while the same excitation is slightly higher with 1.97 and 2.01 eV for **PorTrip** and **PorDbtc**, respectively (see Tables S3–S8). Furthermore, the calculated spectra perfectly reproduce the bathochromic shift of the main transition observed in the absorption experiments (Figure 7).

## Conclusions

In summary, we presented the synthesis of three *β*‐*meso* five‐ring‐fused porphyrins. In detail, fragments containing two (naphthalene), four (triphenylene), and six (dibenzotetracene) benzene rings were coupled to the macrocycle, resulting in three novel “n‐doped” nanographene motifs. The presented synthetic methodology is based on introducing the aromatic building blocks via cross‐coupling reactions into the periphery of tailor‐made nickel‐porphyrins, followed by oxidative cyclodehydrogenation. Hereby, the overall protocols are kept as straightforward as possible and proceed in good to excellent yields. While for the naphthalene and triphenylene derivatives, the final planarization step runs smoothly, the fusion of the dibenzotetracene substituent gave interesting insights into the course of the reaction, indicating a step‐wise bond formation, which can be controlled by adjusting the experimental parameters. Although strongly planarized, the fused molecules retained splendid solubility in common organic solvents, and NMR spectroscopic techniques unambiguously confirmed structure of the conjugates. The optical properties were probed by absorption spectroscopy, revealing significant flattening and bathochromic shifting of the absorption curves of all conjugates. Cross‐comparison showed a progressively more intensely red‐shifted absorption that is proportional to the size of the π‐system. More information regarding the geometric conformation and electronic structure of the architectures was gained through DFT calculations on a hybrid functional level of theory. Here, we discovered sterically induced helicity in two of the conjugates, leading to a non‐planar three‐dimensional structure. Analysis of orbital energies showed a notable reduction in energy gaps compared to ordinary porphyrins, as well as influences of the structural deformation due to the induced helicity. TD‐DFT calculations affirm these results and excellently replicate the experimentally observed red‐shift with increasing π‐system size. Currently, we are working on a more sophisticated photophysical characterization of the presented structures as well as the expansion of the scope of *β*‐*meso*‐fused nanographene‐porphyrin hybrids to further elucidate the influence the five‐ring formation has on the carbon scaffold and its properties.

## Conflict of Interests

The authors declare no conflict of interest.

1

## Supporting information

As a service to our authors and readers, this journal provides supporting information supplied by the authors. Such materials are peer reviewed and may be re‐organized for online delivery, but are not copy‐edited or typeset. Technical support issues arising from supporting information (other than missing files) should be addressed to the authors.

Supporting Information

## Data Availability

The data that support the findings of this study are available in the supplementary material of this article.
