# Supplementary material for: Panchromatic PAH‐Porphyrin Hybrids with a Step‐Wise Increasing π‐System
Source: ChemistryOpen. 2025 Feb 11;14(3):e202400481. doi: 10.1002/open.202400481 (PMC11891458; doi:10.1002/open.202400481)
Supplement: Supplementary file 1 — Supporting Information [file OPEN-14-e202400481-s001.pdf]

ChemistryOpen

Supporting Information

Panchromatic PAH-Porphyrin Hybrids with a Step-Wise Increasing π-System

Christoph Oleszak, Christian L. Ritterhoff, Bernd Meyer,* and Norbert Jux*

Table of Content

1 General Information **S2**

2 Synthetic Procedures **S3**

2.1 Synthesis of trimesityl-porphyrin precursor **S3**

2.2 Synthesis of fused naphthalene-porphyrin PorNaph **S5**

2.3 Synthesis of fused triphenylene-porphyrin PorTrip **S7**

2.4 Synthesis of fused dibenzotetracene-porphyrin PorDbtc **S9**

3 Spectral Appendix **S14**

4 DFT Calculations **S48**

5 References **S62**

1 General Information

All chemicals were purchased from Sigma-Aldrich and used without any further purification. Solvents were distilled prior to usage. Dichloromethane was neutralized with K_2CO_3 before distillation. Thin layer chromatography (TLC) was performed on Merck silica gel 60 F524, detected by UV-light (254 nm, 366 nm). Column chromatography and flash column chromatography were performed on Macherey-Nagel silica gel 60 M (deactivated, 230−400 mesh, 0.04−0.063 mm). NMR spectroscopy was performed on Bruker Avance Neo Cryo-Probe DCH (^{1}H: 600 MHz, ^{13}C: 150 MHz), Bruker Avance Neo 500 (^{1}H: 500 MHz, ^{13}C: 126 MHz), and Bruker Avance 400 (^{1}H: 400 MHz, ^{13}C{^{1}H}: 101 MHz). Deuterated solvents were purchased from Sigma-Aldrich and used as received. Chemical shifts are referenced to residual protic impurities in the solvents (^{1}H: $CHCl_3$: 7.24 ppm) and (^{1}H: CH_2Cl_2: 5.32 ppm) or the deuterated solvent itself (^{13}C{^{1}H}: $CDCl_3$: 77.0 ppm) and (^{13}C{^{1}H}: CD_2Cl_2: 53.8 ppm). The resonance multiplicities are indicated as “s” (singlet), “d” (doublet), “t” (triplet), “q” (quartet), and “m” (multiplet). Signals referred to as “bs” (broad singlet) are not clearly resolved or significantly broadened. IR spectra were recorded on a Bruker FT-IR Tensor 27 spectrometer with a Pike MIRacle ATR unit. LDI/MALDI-ToF mass spectrometry was performed on a Bruker Ultraflex Extreme machine. In case of MALDI, the following matrices were used: 2,5-dihydroxybenzoic acid (DHB) or *trans*-2-[3-(4-*tert*-butylphenyl)-2-methyl-2-propenyl-idene]-malononitrile (DCTB). High-resolution mass spectrometry (MS) was performed on an ESI/APPI-ToF mass spectrometer Bruker maXis 4G UHR MS/MS spectrometer, a Bruker micrOTOF II focus TOF MS spectrometer, or on a MALDI-ToF Bruker Ultraflex Extreme spectrometer. Microwave reactions were carried out in a monomode microwave reactor Biotage Initiator+ with an external IR surface temperature sensor. The microwave-assisted reactions were carried out exclusively in the fixed hold-time mode using an external IR temperature sensor. UV/vis spectroscopy was carried out on a Varian Cary 5000 UV−vis−NIR spectrometer.

2 Synthetic Procedures

2.1 Synthesis of trimesityl-porphyrin precursor

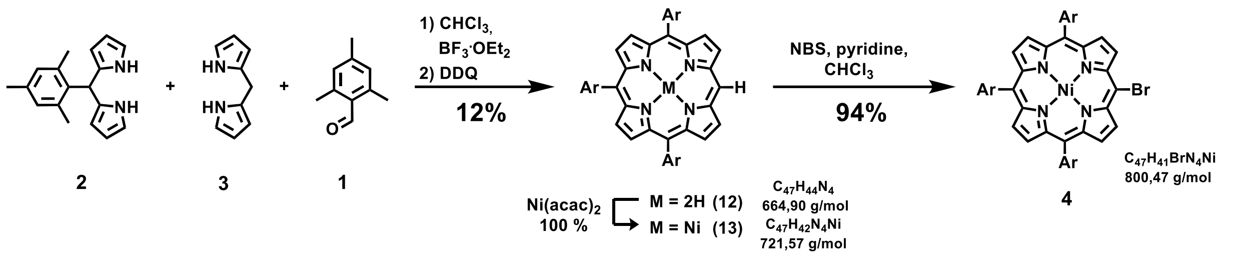

Scheme S1. Synthesis of trimesityl-porphyrin **4**. Ar = mesityl.

Nickel-5,10,15-Trimesitylporphyrin 13

Ethanol (2 mL) stabilized $CHCl_3$ (775 mL) was degassed for 20 min (bubbling N_2 through the solution). Dipyrromethane **3** (284 mg, 1.94 mmol, 1 equiv), mesitaldehyde **1** (572 µL, 3.88 mmol, 2 equiv), and mesityl-dipyrromethane **2** (513 mg, 1.94 mmol, 1 equiv) were added to the solution and the reaction was stirred for 5 min at rt. $BF_3{\cdot}OEt_2$ (316 µL, 2.57 mmol, 0.66 equiv) was added, and the solution was stirred for 1 h at rt under the exclusion of light. DDQ (1.32 g, 5.81 mmol, 1.5 equiv) was added, and the mixture was stirred for a further 45 min. The acid was quenched via the addition of NEt_3 (3.6 mL), and the solvent was removed. The crude was purified by column chromatography (SiO_2, hexanes/CH_2Cl_2, 3:1, Ø 8 x 40 cm). The first isolated fraction contained both A_4-porphyrin and the target A_3B porphyrin, which were subsequently separated by a second column (SiO_2, hexanes/toluene, 3:1, Ø 8 x 40 cm, 2nd band). The product **12** was obtained as a purple solid. Free-base porphyrin **12** (125 mg, 188 µmol, 1 equiv) and $Ni(acac)_2$ (241 mg, 940 µmol, 5 equiv.) were dissolved in toluene (30 mL) and heated to reflux (heat-on temperature: 140 °C) for 5 h. The solvent was removed under reduced pressure, the product was poured over a plug (SiO_2, CH_2Cl_2, Ø 3 x 6 cm), and afterward recrystallized from CH_2Cl_2/MeOH yielding 12% of nickel-porphyrin **13** (136 mg, 188 µmol).

^{1}H NMR (400 MHz, $CDCl_3$, rt): δ [ppm]: 9.79 (s, 1H), 9.07 (d, *J* = 4.8 Hz, 2H), 8.69 (d, *J* = 4.7 Hz, 2H), 8.57 (s, 4H), 7.22 (m, 6H), 2.56 (m, 9H), 1.79 (s, 18H).

^{13}C NMR (101 MHz, $CDCl_3$, rt): δ [ppm]: 142.93, 142.74, 142.59, 142.37, 139.11, 139.08, 137.88, 137.63, 137.59, 137.52, 137.44, 132.17, 131.24, 131.21, 131.15, 129.04, 128.23, 127.72, 127.69, 125.30, 117.32, 116.81, 104.26, 21.48, 21.39.

UV/Vis (CH_2Cl_2): *λ* [nm] (*ε* [$M^{-1}cm^{-1}$]): 407 (250000), 521 (18000), 553 (5000).

HRMS (MALDI, CH_2Cl_2) for $C_{47}H_{42}N_4Ni$ (M^+) calcd.: 720.2757, found: 720.2775.

TLC: R_f [%]: 0.74 (hexanes/CH_2Cl_2 - 2:1).

Nickel-(5-bromo)-10,15,20-trimesitylporphyrin 4

To a solution of $CHCl_3$ (11 mL), pyridine (250 µL) and porphyrin **13** (130 mg, 180 µmol, 1 equiv) NBS (32 mg, 180 µmol, 1 equiv) in $CHCl_3$ (3 mL) was added slowly at rt. The mixture was stirred for 15 min at rt before the reaction was quenched with acetone (3 mL). The solvents were removed under reduced pressure, and the crude was purified by silica plug filtration (hexanes/CH_2Cl_2 - 2:1, Ø 3 x 12 cm). The product **4** was obtained as a dark-orange solid in 94% yield (135 mg, 169 µmol).

^{1}H NMR (400 MHz, $CDCl_3$, rt): δ [ppm]: 9.46 (d, *J* = 5.0 Hz, 2H), 8.60 (d, *J* = 5.0 Hz, 2H), 8.48 (s, 4H), 7.19 - 7.17 (m, 6H), 2.55 (m, 9H), 1.79 (m, 18H).

^{13}C NMR (101 MHz, $CDCl_3$, rt): δ [ppm]: 143.04, 143.00, 142.76, 142.43, 139.00, 138.95, 137.80, 137.75, 136.94, 133.20, 132.03, 131.84, 131.80, 127.77, 117.71, 117.54, 101.91, 21.46, 21.37.

UV/Vis (CH_2Cl_2): *λ* [nm] (*ε* [$M^{-1}cm^{-1}$]): 415 (225000), 530 (17000).

HRMS (MALDI, CH_2Cl_2) for $C_{47}H_{41}BrN_4Ni$ (M^+) calcd.: 798.1863, found: 798.1853.

TLC: R_f [%]: 0.77 (hexanes/CH_2Cl_2 - 2:1).

2.2 Synthesis of fused naphthalene-porphyrin PorNaph

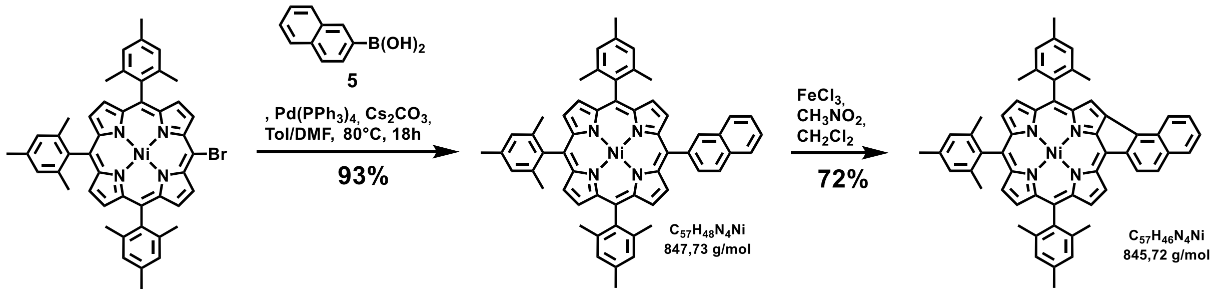

4 6 PorNaph

Scheme S2. Synthesis of fused naphthalene-porphyrin **PorNaph**.

Nickel-naphthalene-porphyrin 6

Nickel-(5-bromo)-10,15,20-trimesitylporphyrin **4** (50.0 mg, 62.5 µmol, 1 equiv), naphthalen-2-yl-boronic-acid **5** (11.3 mg, 65.5 µmol, 1.05 equiv), Cs_2CO_3 (60.5 mg, 188 µmol, 3 equiv) and $Pd(PPh_3)_4$ (14.5 mg, 12.5 µmol, 0.2 equiv) were dissolved in toluene (5 mL) and DMF (2.5 mL) and were degassed. The reaction was heated with an oil bath to 80 °C for 18 h. The solvent was removed, and the crude was purified by silica plug filtration (SiO_2, hexanes/CH_2Cl_2 - 1:1, Ø 3 cm x 8 cm). Further purification was achieved by column chromatography (SiO_2, hexanes/CH_2Cl_2 - 4:1, Ø 7 cm x 40 cm). After recrystallization from CH_2Cl_2/MeOH the product **6** was obtained as a red crystalline solid in 93% yield (49.2 mg, 58.0 µmol).

^{1}H NMR (400 MHz, $CDCl_3$, rt): δ [ppm]: 8.69 (d, *J* = 4.9 Hz, 2H), 8.59-8.54 (m, 6H), 8.48 (s, 1H), 8.23-8.20 (m, 1H), 8.12-8.09 (m, 2H), 8.02-8.00 (m, 1H), 7.68-7.63 (m, 2H), 7.20 (s, 6H), 2.56 (m, 9H), 1.82 (m, 18H).

^{13}C NMR (101 MHz, $CDCl_3$, rt): δ [ppm]: 142.93, 142.65, 142.62, 142.56, 139.09, 138.86, 137.63, 137.41, 137.35, 132.78, 132.68, 132.26, 132.24, 131.90, 131.42, 130.96, 128.41, 127.96, 127.76, 127.74, 126.80, 126.47, 125.96, 118.24, 117.24, 116.95, 21.53, 21.43.

UV/Vis (CH_2Cl_2): *λ* [nm] (*ε* [$M^{-1}cm^{-1}$]): 415 (240000), 527 (18000).

HRMS (MALDI, CH_2Cl_2) for $C_{57}H_{48}N_4Ni$ (M^+) calcd.: 846.3227, found: 846.3239.

TLC: R_f [%]: 0.65 (hexanes/CH_2Cl_2 - 3:1).

Fused nickel-naphthalene-porphyrin PorNaph

A 20 mL vial was filled with a solution of nickel-naphthalene-porphyrin **6** (16.0 mg, 18.9 µmol, 1 equiv) in CH_2Cl_2 (10 mL) and cooled with an ice bath. The solution was degassed (bubbling N_2 through the solution for 15 min). The N_2 flow through the solution was increased, and a solution of dry $FeCl_3$ (49.0 mg, 302 µmol, 16 equiv) in CH_3NO_2 (0.2 mL) was added. The N_2 bubbling through the solution was stopped 15 min after $FeCl_3$ was added, and the solution was stirred under slow warming for 3 h. MeOH (10 mL) was added to quench the reaction. After adding NEt_3 (1 mL), the solvent was removed, and the crude was purified by a silica plug (hexanes/ CH_2Cl_2 - 1:1, Ø 3 x 10 cm). Further purification was achieved by column chromatography (SiO_2, hexanes/CH_2Cl_2 - 4:1, Ø 7 cm x 30 cm). The product **PorNaph** was obtained as a dark-brown solid in 72% yield (11.5 mg, 13.6 µmol).

^{1}H NMR (601 MHz, CD_2Cl_2, rt): δ [ppm]: 8.94 (d, *J* = 4.9 Hz, 1H), 8.33 (d, *J* = 4.9 Hz, 1H), 8.07 (d, *J* = 8.3 Hz, 1H), 8.03 (d, *J* = 4.8 Hz, 1H), 7.98-7.92 (m, 3H), 7.86 (d, *J* = 8.2 Hz, 1H), 7.63 (s, 1H), 7.57-7.54 (m, 1H), 7.51 (d, *J* = 8.3 Hz, 1H), 7.30-7.27 (m, 1H), 7.23-7.18 (m, 5H), 7.15 (s, 2H), 2.55 (s, 3H), 2.54 (s, 3H), 2.50 (s, 3H), 1.98 (s, 6H), 1.87 (s, 6H), 1.85 (s, 6H).

^{13}C NMR (151 MHz, CD_2Cl_2, rt): δ [ppm]: 156.35, 148.23, 147.40, 147.07, 145.41, 144.98, 144.07, 143.85, 142.22, 139.08, 138.85, 138.82, 138.27, 138.14, 137.24, 136.96, 135.78, 135.24, 134.74, 134.19, 132.38, 130.82, 130.49, 129.98, 129.57, 129.49, 129.20, 128.13, 128.10, 127.58, 127.03, 126.17, 125.25, 123.77, 123.42, 123.00, 122.27, 117.82, 113.01, 21.50, 21.47, 21.44, 21.40, 21.31, 21.16.

UV/Vis (CH_2Cl_2): *λ* [nm] (*ε* [$M^{-1}cm^{-1}$]): 385 (60000). 442 (65000), 486 (62500), 582 (7000), 631 (3000).

HRMS (MALDI, CH_2Cl_2) for $C_{57}H_{46}N_4Ni$ (M^+) calcd.: 844.3070, found: 844.3079.

TLC: R_f [%]: 0.75 (hexanes/CH_2Cl_2 - 2:1).

2.3 Synthesis of fused triphenylene-porphyrin PorTrip

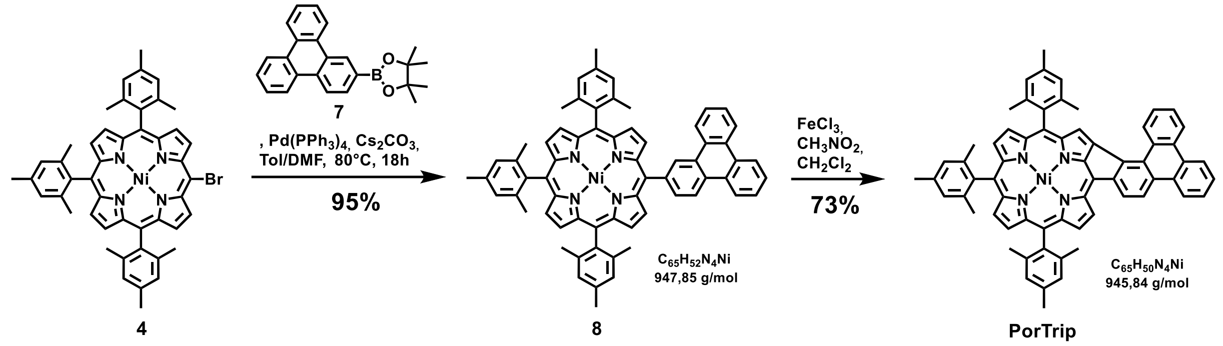

Scheme S3. Synthesis of fused triphenylene-porphyrin **PorTrip**.

Nickel-Triphenlyene-Porphyrin 8

Nickel-(5-bromo)-10,15,20-trimesitylporphyrin **4** (40.0 mg, 40.0 µmol, 1 equiv), 4,4,5,5-tetramethyl-2-(triphenylen-2-yl)-1,3,2-dioxaborolane **7** (19.5 mg, 55.0 µmol, 1.1 equiv), Cs_2CO_3 (48.4 mg, 150 µmol, 3 equiv) and $Pd(PPh_3)_4$ (11.6 mg, 10.0 µmol, 0.2 equiv) were dissolved in toluene (4 mL) and DMF (2 mL) and were degassed. The reaction was heated with an oil bath to 80 °C for 18 h. The solvent was removed, and the crude was purified by silica plug filtration (SiO_2, hexanes/CH_2Cl_2 - 1:1, Ø 3 cm x 8 cm). After recrystallization from CH_2Cl_2/MeOH the product **8** was obtained as a red crystalline solid in 95% yield (45.0 mg, 47.5 µmol).

^{1}H NMR (400 MHz, $CDCl_3$, rt): δ [ppm]: 9.32 (d, *J* = 1.8 Hz, 1H), 8.93 (d, *J* = 8.5 Hz, 1H), 8.90-8.85 (m, 1H), 8.78 – 8.72 (m, 4H), 8.63-8.61 (m, 1H), 8.58 (d, *J* = 4.9 Hz, 2H), 8.56 (s, 4H), 8.36 (dd, *J* = 8.3, 1.7 Hz, 1H), 7.79-7.73 (m, 2H), 7.69-7.65 (m, 1H), 7.57-7.53 (m, 1H), 7.20-7.19 (m, 6H), 2.56 (s, 3H), 2.54 (s, 6H), 1.84-1.83 (m, 18H).

^{13}C NMR (101 MHz, $CDCl_3$, rt): δ [ppm]: 142.87, 142.67, 142.63, 142.59, 140.20, 139.09, 139.07, 137.65, 137.36, 137.32, 132.68, 132.27, 131.48, 131.12, 130.42, 130.14, 129.88, 129.80, 129.15, 128.65, 128.41, 127.76, 127.75, 127.54, 127.50, 127.39, 123.63, 123.53, 123.46, 121.70, 118.18, 117.30, 116.99, 21.54, 21.44, 21.41.

UV/Vis (CH_2Cl_2): *λ* [nm] (*ε* [$M^{-1}cm^{-1}$]): 416 (280000), 528 (25000).

HRMS (MALDI, CH_2Cl_2) for $C_{65}H_{52}N_4Ni$ (M^+) calcd.: 946.3540, found: 946.3539.

TLC: R_f [%]: 0.45 (hexanes/CH_2Cl_2 - 3:1).

Fused Nickel-Triphenylene-Porphyrin PorTrip

A 20 mL vial was filled with a solution of nickel-triphenylene-porphyrin **8** (20.0 mg, 21.1 µmol, 1 equiv) in CH_2Cl_2 (10 mL) and cooled with an ice bath. The solution was degassed (bubbling N_2 through the solution for 15 min). The N_2 flow through the solution was increased, and a solution of dry $FeCl_3$ (55.0 mg, 338 µmol, 16 equiv) in CH_3NO_2 (0.2 mL) was added. The N_2 bubbling through the solution was stopped 15 min after $FeCl_3$ was added, and the solution was stirred under slow warming for 24 h. MeOH (10 mL) was added to quench the reaction. After adding NEt_3 (1 mL), the solvent was removed, and the crude was purified by a silica plug (hexanes/ CH_2Cl_2 - 1:1, Ø 3 x 10 cm). Further purification was achieved by column chromatography (SiO_2, hexanes/CH_2Cl_2 - 4:1, Ø 7 cm x 35 cm). The product **PorTrip** was obtained as a dark-brown solid in 73% yield (14.6 mg, 15.4 µmol).

^{1}H NMR (601 MHz, CD_2Cl_2, rt): δ [ppm]: 9.31 (d, J = 8.0 Hz, 1H), 9.09 (d, J = 4.9 Hz, 1H), 8.55-8.44 (m, 3H), 8.42 (d, J = 4.9 Hz, 1H), 8.32-8.24 (m, 2H), 8.15-7.99 (m, 5H), 7.68-7.63 (m, 1H), 7.58-7.56 (m, 2H), 7.49-7.46 (m, 1H), 7.21-7.17 (m, 6H), 2.55 (m, 6H), 2.52 (s, 3H), 1.98 (s, 6H), 1.87-1.86 (m, 12H).

^{13}C NMR (151 MHz, CD_2Cl_2, rt): δ [ppm]: 154.94, 150.20, 149.13, 146.50, 145.91, 144.99, 144.88, 144.17, 144.00, 141.73, 139.11, 138.89, 138.84, 138.41, 138.20, 137.28, 136.99, 135.61, 135.52, 134.12, 132.31, 131.99, 131.40, 131.11, 130.53, 130.39, 130.14, 129.96, 128.47, 128.13, 128.11, 128.03, 127.98, 127.67, 127.35, 127.02, 126.18, 126.15, 124.46, 123.85, 123.68, 123.58, 123.50, 122.99, 121.73, 118.27, 112.14, 21.52, 21.47, 21.45, 21.41, 21.36, 21.23.

UV/Vis (CH_2Cl_2): λ [nm] (ε [$M^{-1}cm^{-1}$]): 418 (40000), 463 (69000), 488 (73000), 581 (6000), 626 (3000).

HRMS (MALDI, CH_2Cl_2) for $C_{65}H_{50}N_4Ni$ (M^+) calcd.: 944.3383, found: 944.3392.

TLC: R_f [%]: 0.40 (hexanes/CH_2Cl_2 - 3:1).

2.4 Synthesis of fused dibenzotetracene-porphyrin PorDbtc

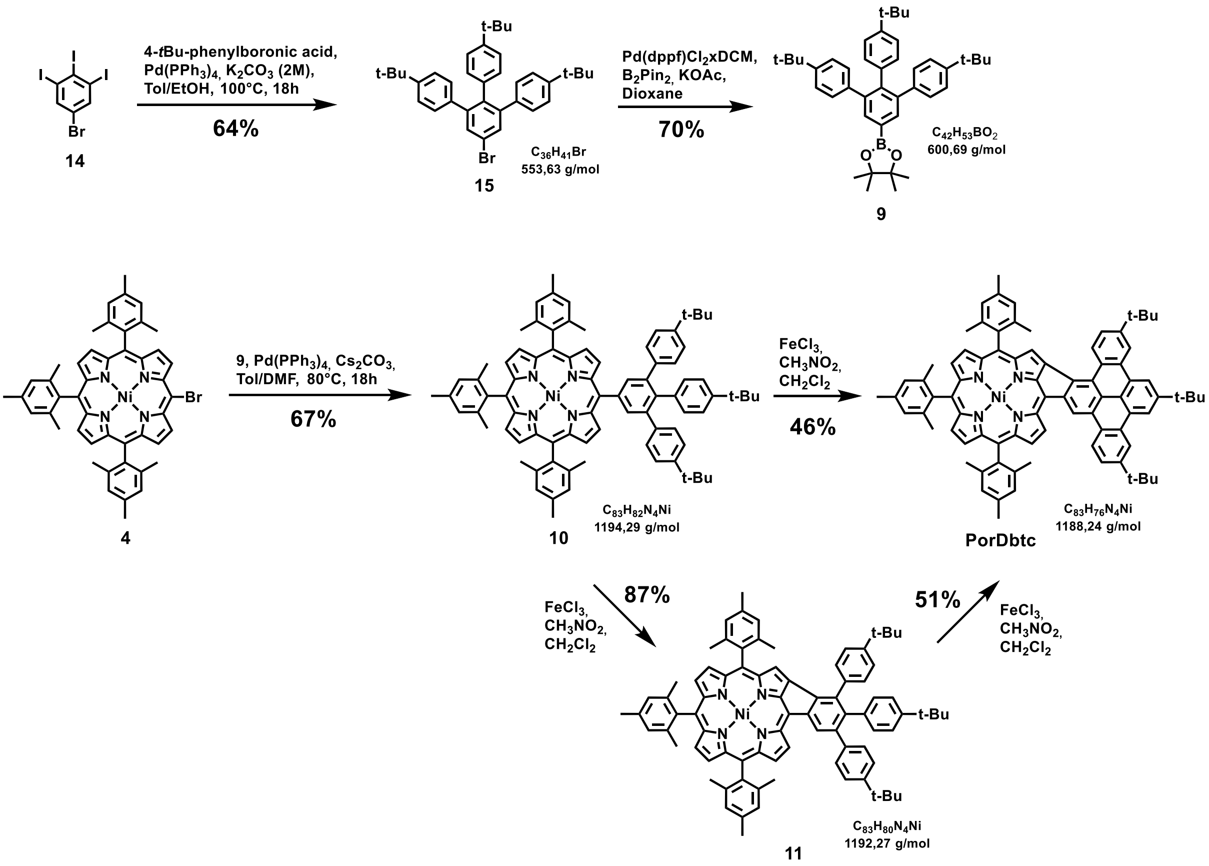

Scheme S4. Synthesis of fused dibenzotetracene-porphyrin **PorDbtc**.

3,4,5-Tri-(4-*tert*-butylphenyl)-bromobenzene 15

3,4,5-Tri-iodo-bromobenzene **14*** (352 mg, 0.66 mmol, 1 equiv), 4-*t*Bu-phenyl-boronic-acid (352 mg, 1.98 mmol, 3 equiv), K_2CO_3 (2M in H_2O, 1.4 mL) and $Pd(PPh_3)_4$ (92 mg, 0.08 µmol, 0.12 equiv) were dissolved in toluene (4 mL) and EtOH (0.4 mL) and were degassed. The reaction was heated with an oil bath to 100 °C for 18 h. The aqueous phase was extracted with EtOAc (2 x 50 mL), and the combined organic layers were washed with brine (100 mL). After drying over Na_2SO_4, the solvent was removed, and the crude was purified by column chromatography (SiO_2, hexanes/CH_2Cl_2 - 4:1, Ø 5 cm x 30 cm). After recrystallization from CH_2Cl_2/MeOH the product **15** was obtained as a colorless crystalline solid in 64% yield (233 mg, 421 µmol).

^{1}H NMR (400 MHz, $CDCl_3$, rt): δ [ppm]: 7.56 (s, 2H), 7.16-7.11 (m, 4H), 6.99-6.92 (m, 6H), 6.70-6.64 (m, 2H), 1.25 (s, 18H), 1.17 (s, 9H).

^{13}C NMR (101 MHz, $CDCl_3$, rt): δ [ppm]: 149.34, 148.85, 143.80, 138.47, 137.73, 135.66, 131.75, 131.04, 129.35, 124.37, 123.89, 120.69, 34.34, 34.23, 31.30, 31.26, 31.22.

HRMS (APPI) for $C_{36}H_{42}Br$ (M^+) calcd.: 553.2454, found: 553.2460.

* Synthesized adapting [1]

3,4,5-Tri-(4-*tert*-butylphenyl)-benzene-boronic-ester 9

3,4,5-Tri-(4-*tert*-butylphenyl)-bromobenzene **15** (208 mg, 376 µmol, 1 equiv), bis-(pinacolato)-diboron (105 mg, 414 µmol, 1.1 equiv), KOAc (111 mg, 1.13 mmol, 3 equiv) and $Pd(dppf)Cl_2$ x CH_2Cl_2 (15.4 mg, 19.0 µmol, 0.05 equiv) were dissolved in dioxane (3 mL) and were degassed. The reaction was heated with an oil bath to 80 °C for 18 h. The crude was purified by filtration through celite (CH_2Cl_2, Ø 3cm x 8 cm). After recrystallization from CH_2Cl_2/MeOH the product **9** was obtained as a colorless crystalline solid in 70% yield (158 mg, 263 µmol).

^{1}H NMR (400 MHz, $CDCl_3$, rt): δ [ppm]: 7.87 (s, 2H), 7.14-7.09 (m, 4H), 7.03-6.99 (m, 4H), 6.97-6.92 (m, 2H), 6.74-6.69 (m, 2H), 1.33 (s, 12H), 1.24 (s, 18H), 1.17 (s, 9H).

^{13}C NMR (101 MHz, $CDCl_3$, rt): δ [ppm]: 148.63, 148.59, 142.17, 141.34, 138.99, 136.65, 135.70, 131.04, 129.57, 124.12, 123.74, 83.75, 34.28, 34.21, 31.30, 31.23, 24.86.

HRMS (APPI) for $C_{42}H_{53}BO_2$ (M^+) calcd.: 600.4133, found: 600.4145.

Nickel-3,4,5-Tri-(4-*tert*-butylphenyl)-benzene-Porphyrin 10

Nickel-(5-bromo)-10,15,20-trimesitylporphyrin **4** (80.0 mg, 100 µmol, 1 equiv), 3,4,5-tri-(4-*tert*-butylphenyl)-benzene-boronic-ester **9** (63.2 mg, 105 µmol, 1.05 equiv), Cs_2CO_3 (97.6 mg, 300 µmol, 3 equiv) and $Pd(PPh_3)_4$ (23.2 mg, 20.0 µmol, 0.2 equiv) were dissolved in toluene (4 mL) and DMF (2 mL) and were degassed. The reaction was heated with an oil bath to 80 °C for 18 h. The solvent was removed, and the crude

was purified by silica plug filtration (SiO_2, hexanes/CH_2Cl_2 - 1:1, Ø 3 cm x 8 cm). Further purification was achieved by size exclusion chromatography (Biobeads SX1, toluene, Ø 5 cm x 130 cm). After recrystallization from CH_2Cl_2/MeOH the product **10** was obtained as a red crystalline solid in 67% yield (113 mg, 66.9 µmol).

^{1}H NMR (400 MHz, CD_2Cl_2, rt): δ [ppm]: 9.04 (d, J = 4.9 Hz, 2H), 8.62 (d, J = 4.9 Hz, 2H), 8.55 (s, 4H), 8.09 (s, 2H), 7.25-7.16 (m, 16H), 7.09-7.02 (m, 2H), 2.57-2.55 (m, 9H), 1.82 (s, 18H), 1.28 (s, 9H), 1.24 (s, 18H).

^{13}C NMR (101 MHz, CD_2Cl_2, rt): δ [ppm]: 149.49, 149.47, 143.20, 142.86, 140.81, 140.14, 139.41, 139.28, 138.93, 138.16, 137.57, 137.51, 137.29, 135.17, 132.88, 131.88, 131.75, 131.71, 131.26, 130.17, 129.34, 128.53, 128.08, 124.77, 124.44, 118.63, 117.50, 117.29, 34.59, 31.44, 31.38, 30.07, 21.49, 21.47, 21.42.

UV/Vis (CH_2Cl_2): *λ* [nm] (*ε* [$M^{-1}cm^{-1}$]): 416 (220000), 528 (18000).

HRMS (MALDI, CH_2Cl_2) for $C_{83}H_{82}N_4Ni$ (M^+) calcd.: 1192.5887, found: 1192.5919.

TLC: R_f [%]: 0.45 (hexanes/CH_2Cl_2 - 3:1).

Fused nickel-dibenzotetracene-porphyrin PorDbtc

A 20 mL vial was filled with a solution of **10** (20.0 mg, 16.7 µmol, 1 equiv) in CH_2Cl_2 (10 mL) and cooled with an ice bath. The solution was degassed (bubbling N_2 through the solution for 15 min). The N_2 flow through the solution was increased, and a solution of dry $FeCl_3$ (43.4 mg, 268 µmol, 16 equiv) in CH_3NO_2 (0.2 mL) was added. The N_2 bubbling through the solution was stopped 15 min after $FeCl_3$ was added, and the solution was stirred under slow warming for 24 h. MeOH (10 mL) was added to quench the reaction. After adding NEt_3 (1 mL), the solvent was removed, and the crude was purified by a silica plug (hexanes/ CH_2Cl_2 - 1:1, Ø 3 x 10 cm). Further purification was achieved by column chromatography (SiO_2, hexanes/CH_2Cl_2 - 4:1, Ø 7 cm x 35 cm). The product **PorDbtc** was obtained as a dark-brown solid in 46% yield (9.12 mg, 7.68 µmol).

Fused Nickel-Dibenzotetracene-Porphyrin PorDbtc (via 11)

A 20 mL vial was filled with a solution of **10** (20.0 mg, 16.7 µmol, 1 equiv) in CH_2Cl_2 (10 mL) and cooled with an ice bath. The solution was degassed (bubbling N_2 through the solution for 15 min). The N_2 flow through the solution was increased, and a solution of dry $FeCl_3$ (43.4 mg, 268 µmol, 16 equiv) in CH_3NO_2 (0.2 mL) was added. The N_2 bubbling through the solution was stopped 15 min after $FeCl_3$ was added, and the solution was stirred under slow warming for 24 h. MeOH (10 mL) was added to quench the reaction. After adding NEt_3 (1 mL), the solvent was removed, and the crude was purified by a silica plug (hexanes/ CH_2Cl_2 - 1:1, Ø 3 x 10 cm). **11** was obtained as a dark-green solid in 87% yield (17.3 mg, 7.68 µmol). **11** was transferred to a 20 mL vial and dissolved in CH_2Cl_2 (10 mL) and cooled with an ice bath. The solution was degassed (bubbling N_2 through the solution for 15 min). The N_2 flow through the solution was increased, and a solution of dry $FeCl_3$ (43.4 mg, 268 µmol, 16 equiv) in CH_3NO_2 (0.2 mL) was added. The N_2 bubbling through the solution was stopped 15 min after $FeCl_3$ was added, and the solution was stirred under slow warming for 24 h. MeOH (10 mL) was added to quench the reaction. After adding NEt_3 (1 mL), the solvent was removed, and the crude was purified by a silica plug (hexanes/ CH_2Cl_2 - 1:1, Ø 3 x 10 cm). Further purification was achieved by column chromatography (SiO_2, hexanes/CH_2Cl_2 - 4:1, Ø 7 cm x 35 cm). The product was obtained as a dark-brown solid in 51% yield (with respect to **11**) (8.79 mg, 7.40 µmol).

Incompletely fused porphyrin 11

^{1}H NMR (400 MHz, $CDCl_3$, rt): δ [ppm]: 9.02 (d, *J* = 5.0 Hz, 1H), 8.41 (d, *J* = 4.9 Hz, 1H), 8.15-8.01 (m, 5H), 7.24-7.16 (m, 10H), 7.13 (s, 2H), 7.02 (s, 2H), 7.00-6.95 (m, 2H), 6.85-6.80 (m, 2H), 6.70 (s, 1H), 2.54 (s, 3H), 2.51 (s, 3H), 2.42 (s, 3H), 1.82-1.79 (m, 18H), 1.29 (s, 9H), 1.26 (s, 9H), 1.18 (s, 9H).

^{13}C NMR (101 MHz, $CDCl_3$, rt): δ [ppm]: 154.89, 149.51, 149.08, 148.51, 148.19, 147.08, 146.41, 145.50, 144.42, 144.13, 143.20, 143.18, 142.76, 141.34, 139.63, 139.48, 138.94, 138.78, 138.72, 138.37, 137.56, 137.53, 137.36, 137.33, 137.27, 137.05, 136.29, 136.18, 135.50, 133.45, 131.54, 131.05, 130.56, 129.77, 129.48, 129.23, 127.74, 127.70, 127.50, 126.61, 125.62, 124.41, 124.39, 123.73, 123.27,

121.62, 120.70, 117.64, 111.75, 34.42, 34.39, 34.24, 31.34, 31.25, 29.70, 21.40, 21.38, 21.34, 21.24.

UV/Vis (CH_2Cl_2): *λ* [nm] (*ε* [$M^{-1}cm^{-1}$]): 384 (36000), 447 (82000), 474 (71000), 573 (9000).

HRMS (MALDI, CH_2Cl_2) for $C_{83}H_{80}N_4Ni$ (M^+) calcd.: 1190.5731, found: 1190.5731.

TLC: R_f [%]: 0.40 (hexanes/CH_2Cl_2 - 3:1).

Fused dibenzotetracene-porphyrin PorDbtc

^{1}H NMR (601 MHz, CD_2Cl_2, rt): δ [ppm]: 9.47-9.41 (m, 2H), 9.33 (d, *J* = 8.4 Hz, 1H), 8.89-8.88 (m, 2H), 8.83 (s, 1H), 8.79 (s, 1H), 8.69 (s, 1H), 8.58-8.56 (m, 1H), 8.24-8.23 (m, 1H), 8.18-8.08 (m, 4H), 7.92-7.90 (m, 1H), 7.61-7.59 (m, 1H), 7.24-7.19 m, 6H), 2.58 (s, 3H), 2.56 (s, 3H), 2.53 (s, 3H), 2.00 (s, 6H), 1.88 (s, 6H), 1.69 (s, 6H), 1.59 (s, 18H), 1.26 (s, 9H).

^{13}C NMR (151 MHz, CD_2Cl_2, rt): δ [ppm]: 155.47, 151.75, 150.75, 149.49, 148.95, 148.89, 146.55, 146.05, 145.02, 144.57, 144.16, 144.05, 141.62, 139.17, 139.05, 138.96, 138.42, 138.21, 138.19, 137.41, 137.10, 135.86, 134.07, 132.72, 132.12, 131.66, 131.20, 130.48, 130.41, 130.27, 130.24, 130.18, 130.11, 129.08, 128.53, 128.23, 128.14, 128.10, 127.96, 127.07, 126.11, 126.05, 125.22, 124.16, 123.76, 123.49, 122.26, 121.28, 119.89, 119.76, 119.32, 118.83, 118.51, 118.08, 112.56, 31.84, 31.54, 31.46, 30.05, 21.57, 21.48, 21.42, 21.39, 21.34, 21.27.

UV/Vis (CH_2Cl_2): *λ* [nm] (*ε* [$M^{-1}cm^{-1}$]): 402 (35000), 474 (62000), 501 (70000), 585 (9000).

HRMS (MALDI, CH_2Cl_2) for $C_{83}H_{76}N_4Ni$ (M^+) calcd.: 1186.5418, found: 1186.5414.

TLC: R_f [%]: 0.40 (hexanes/CH_2Cl_2 - 3:1).

3 Spectral Appendix

^{1}H NMR (400 MHz, $CDCl_3$, rt)

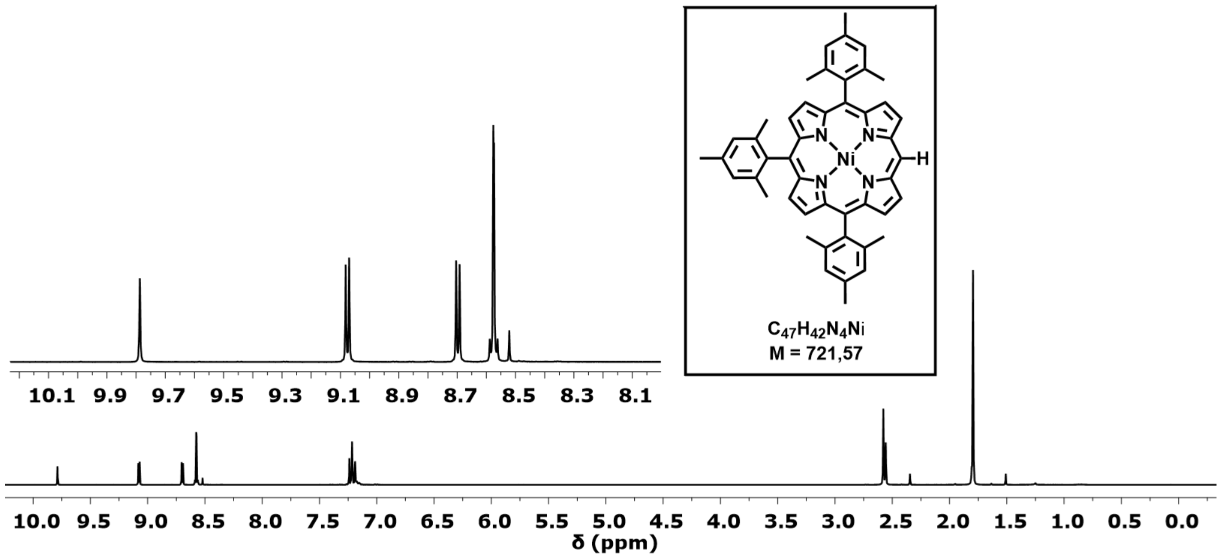

^{13}C NMR (101 MHz, $CDCl_3$, rt)

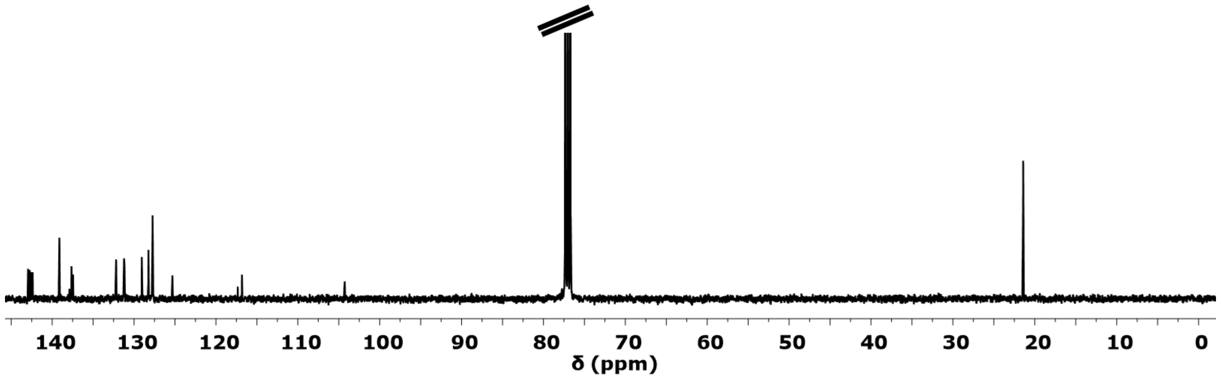

Figure S1. ^{1}H and ^{13}C NMR of **13**.

MS (MALDI)

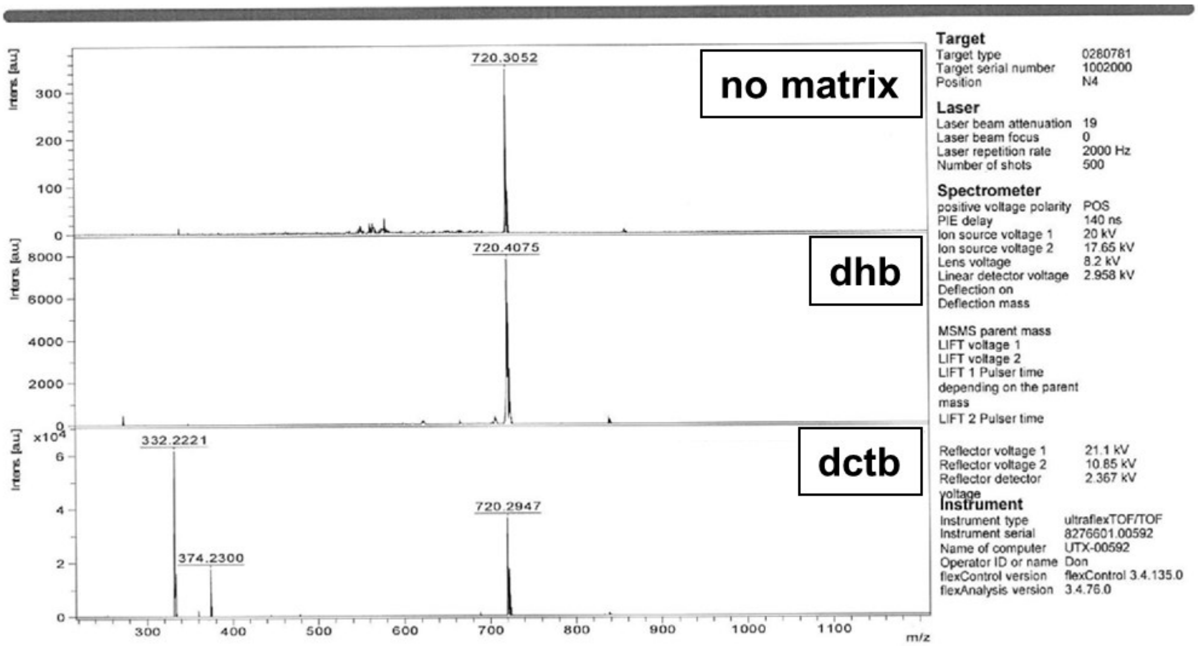

HRMS (MALDI)

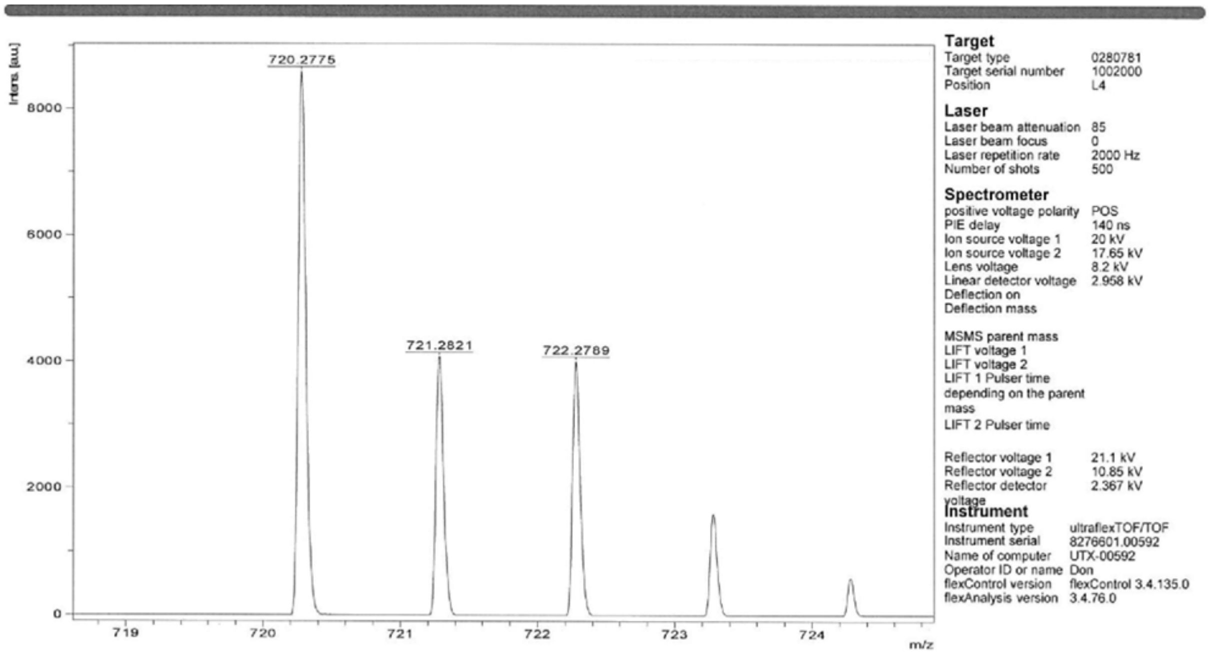

SmartFormula

Formula	Mass	Error	mSigma	DblEq	N rule	Electron Configuration
C 47 H 42 N 4 Ni	720.2757	2.3975	100.6242	29.00	ok	odd

Figure S2. MS/HRMS (MALDI) of **13**.

^{1}H NMR (400 MHz, $CDCl_3$, rt)

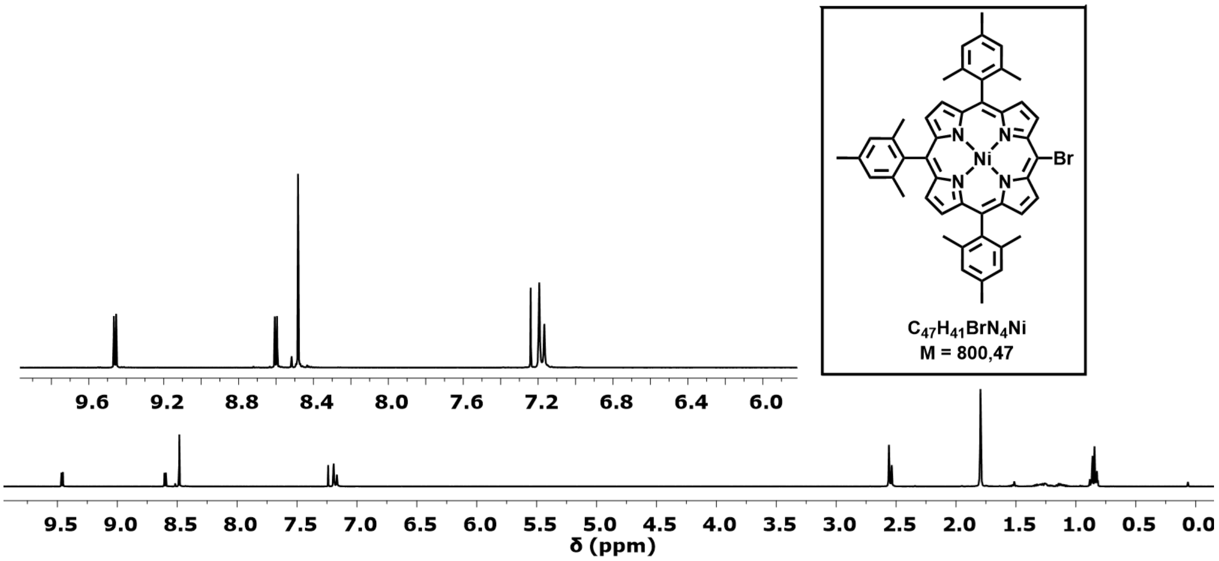

^{13}C NMR (101 MHz, $CDCl_3$, rt)

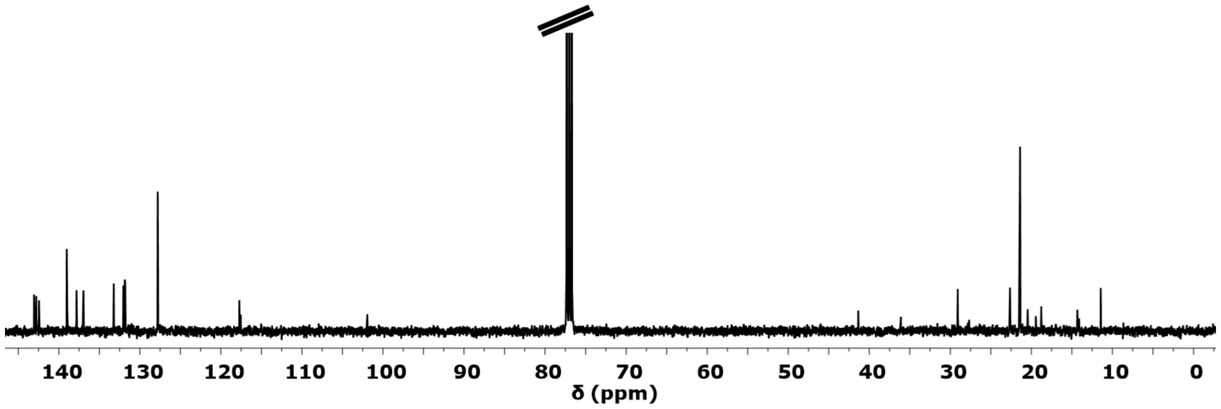

Figure S3. ^{1}H and ^{13}C NMR of **4**.

MS (MALDI)

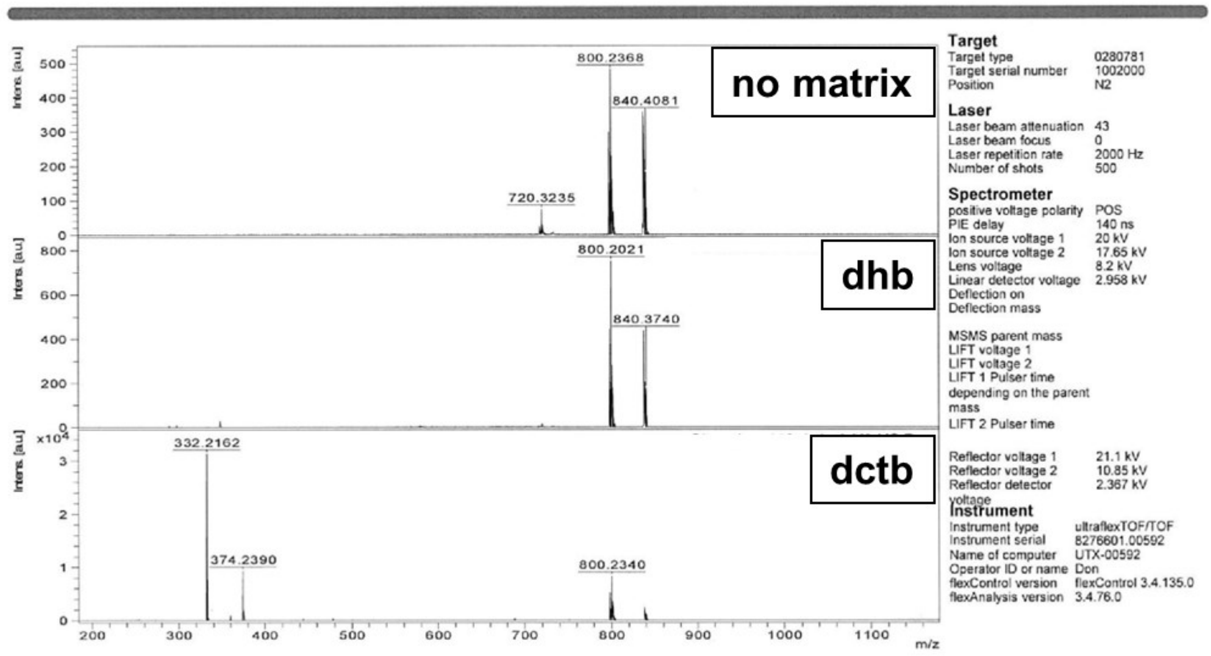

HRMS (MALDI)

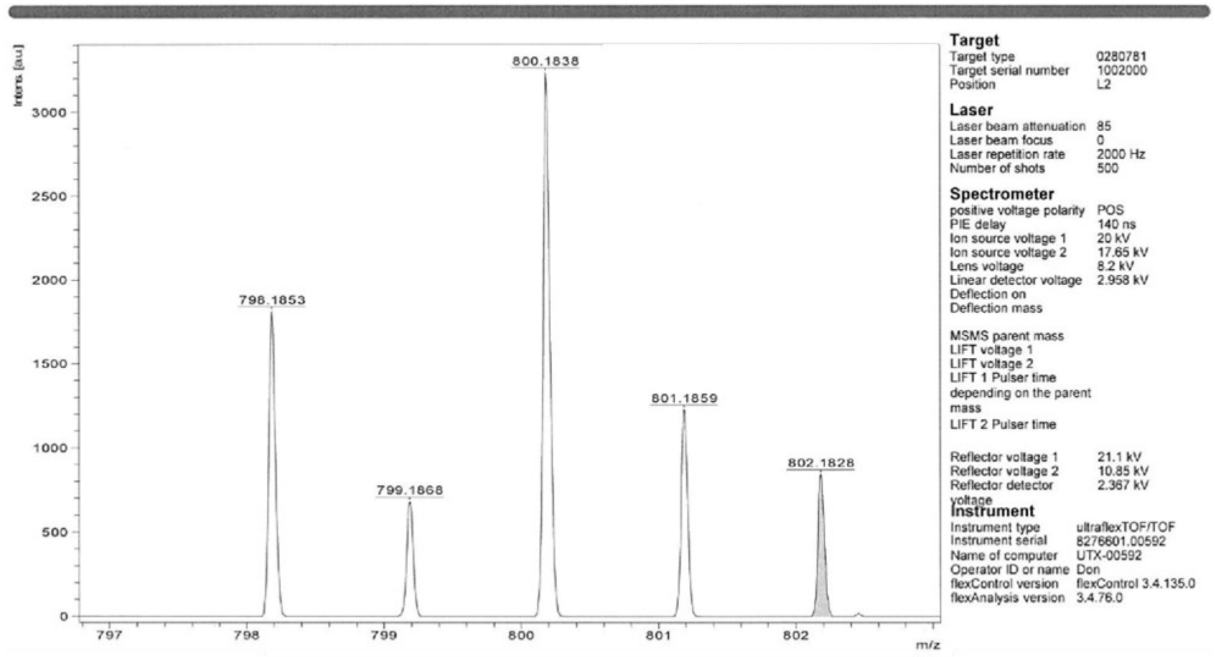

SmartFormula

Formula	Mass	Error	mSigma	DblEq	N rule	Electron Configuration
C 47 H 41 Br N 4 Ni	798.1863	1.1747	247.2498	29.00	ok	odd

Figure S4. MS/HRMS (MALDI) of **4**.

^{1}H NMR (400 MHz, $CDCl_3$, rt)

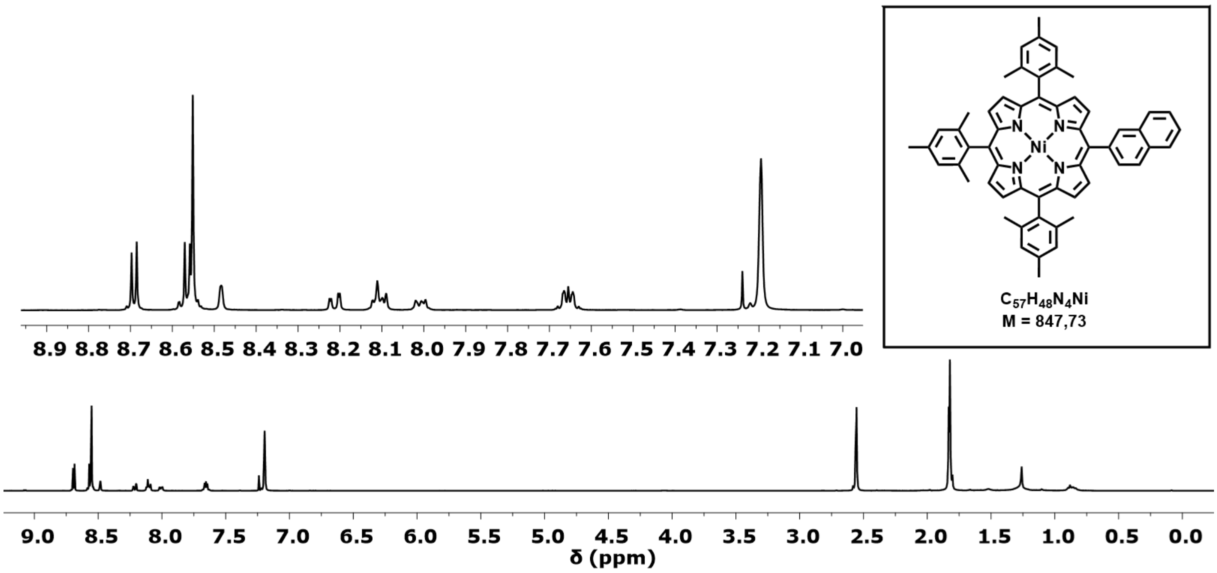

^{13}C NMR (101 MHz, $CDCl_3$, rt)

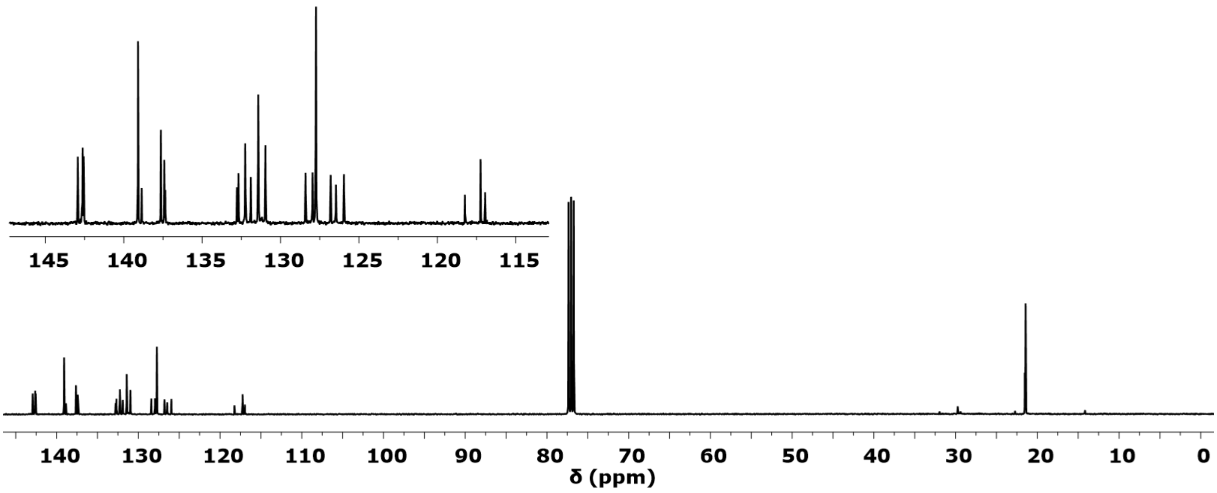

Figure S5. ^{1}H and ^{13}C NMR of **6**.

MS (MALDI)

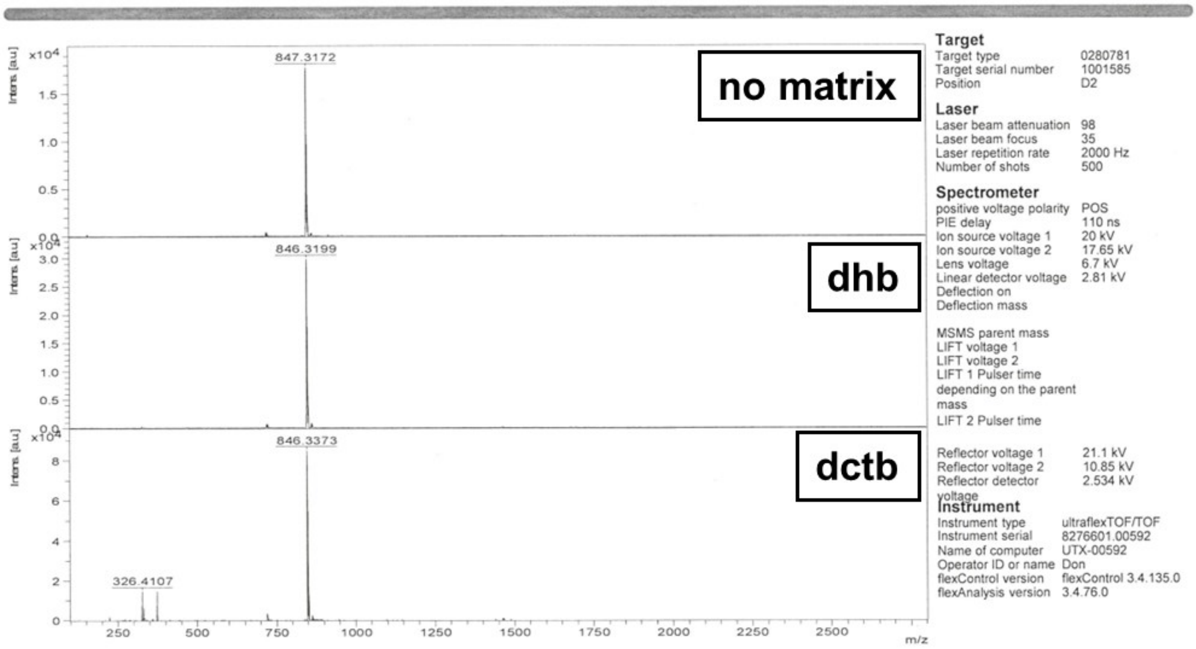

HRMS (MALDI)

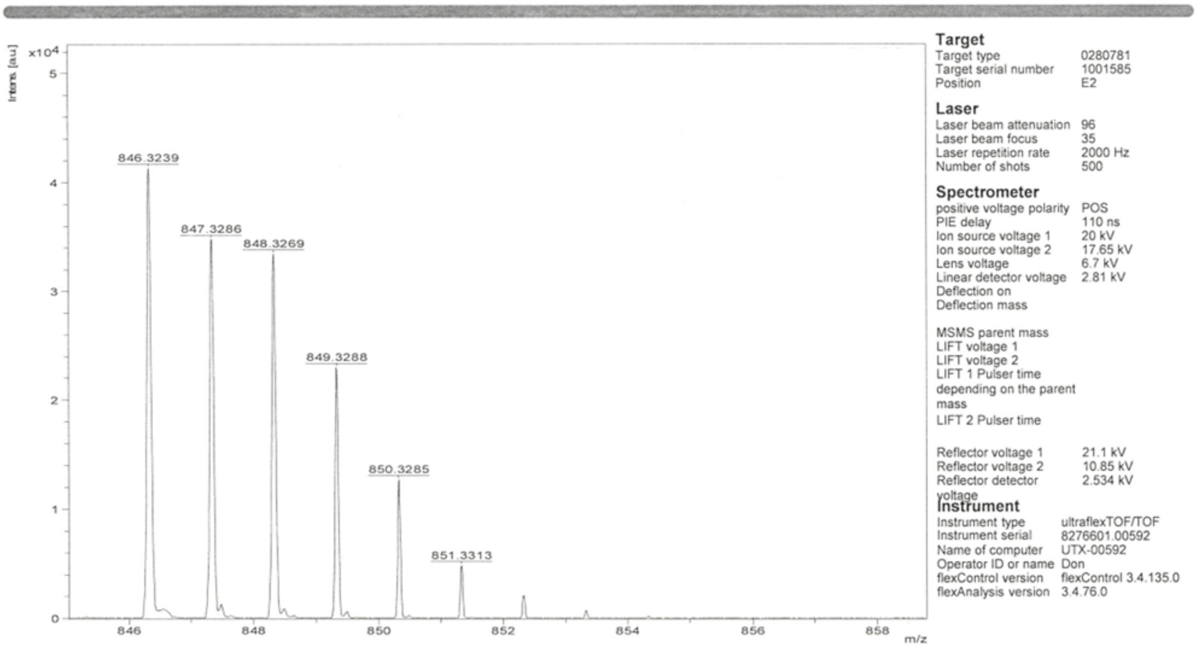

SmartFormula

Formula	Mass	Error	mSigma	DblEq	N rule	Electron Configuration
C 57 H 48 N 4 Ni	846.3227	1.4500	153.3183	36.00	ok	odd

Figure S6. MS/HRMS (MALDI) of **6**.

^{1}H NMR (601 MHz, CD_2Cl_2, rt)

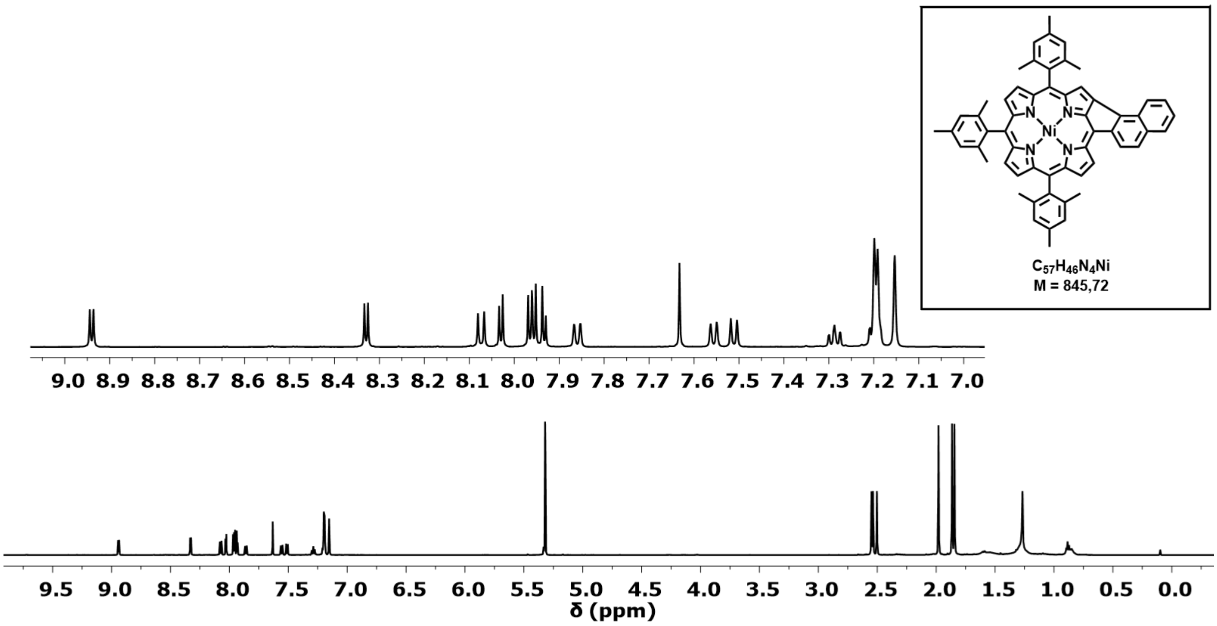

^{13}C NMR - DEPTQ135 (151 MHz, CD_2Cl_2, rt)

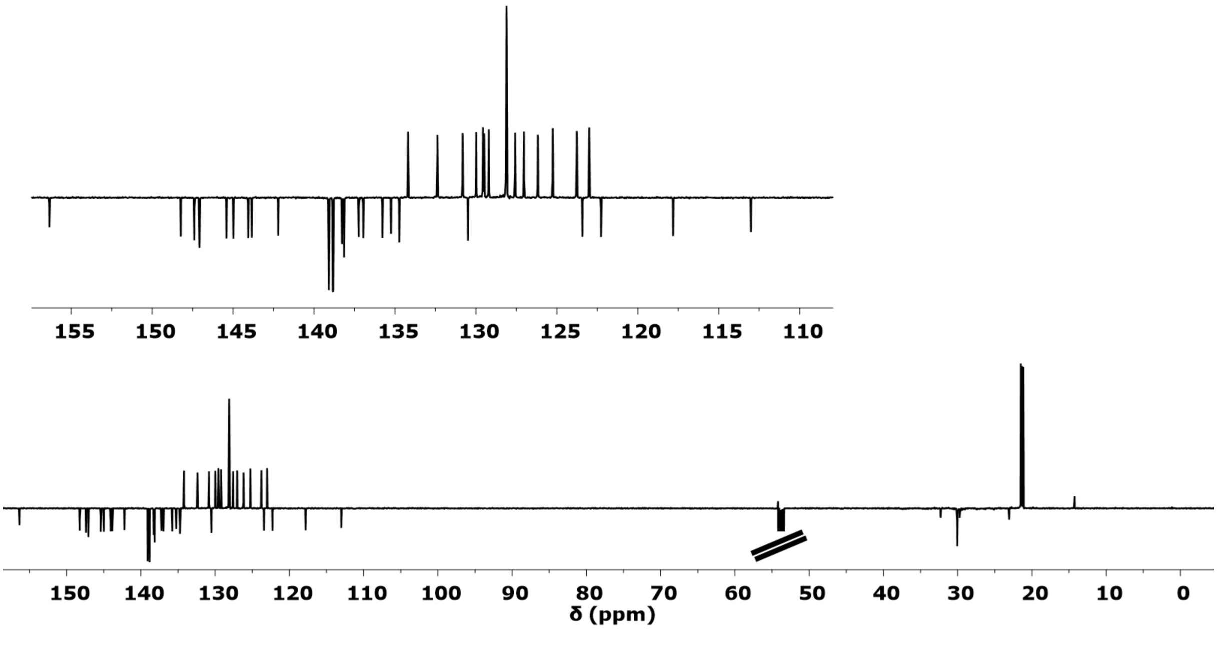

Figure S7. ^{1}H and ^{13}C NMR (DEPTQ135) of **PorNaph**.

^{1}H- ^{1}H COSY (601 MHz, CD_2Cl_2, rt)

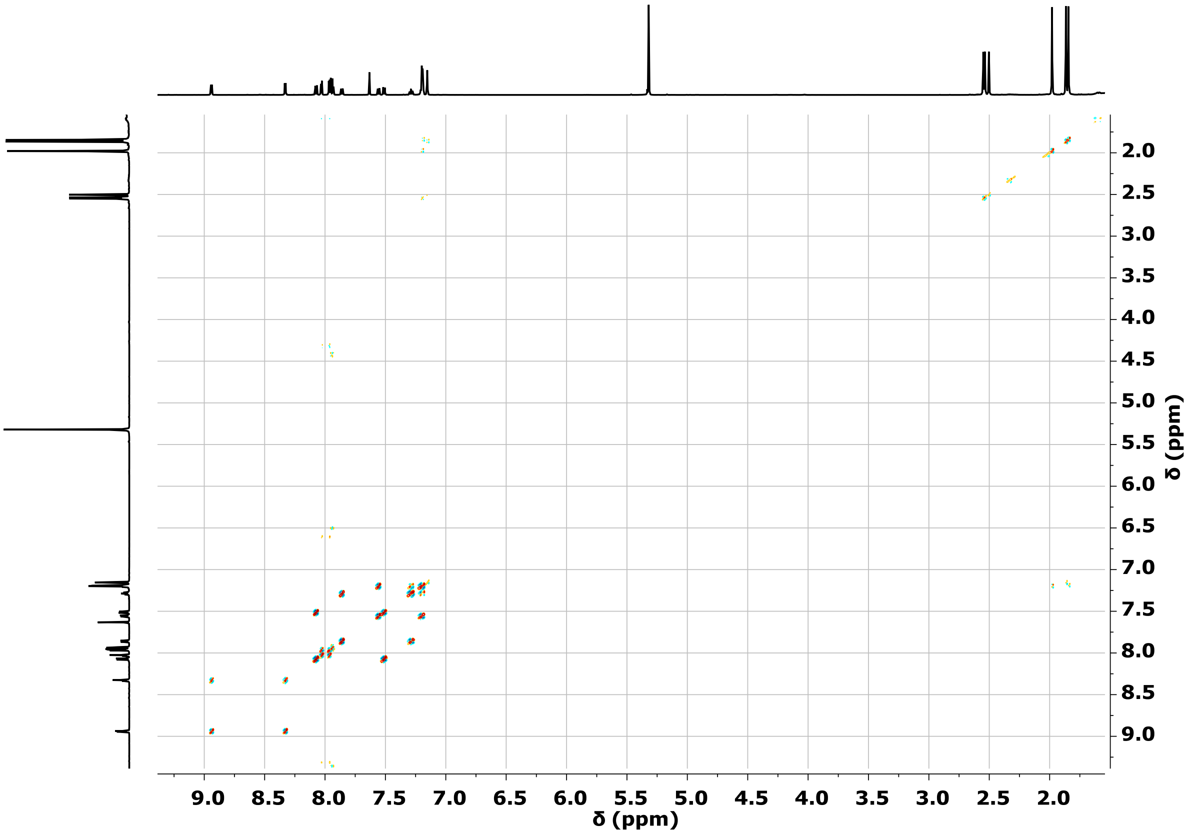

Figure S8. ^{1}H- ^{1}H COSY of **PorNaph**.

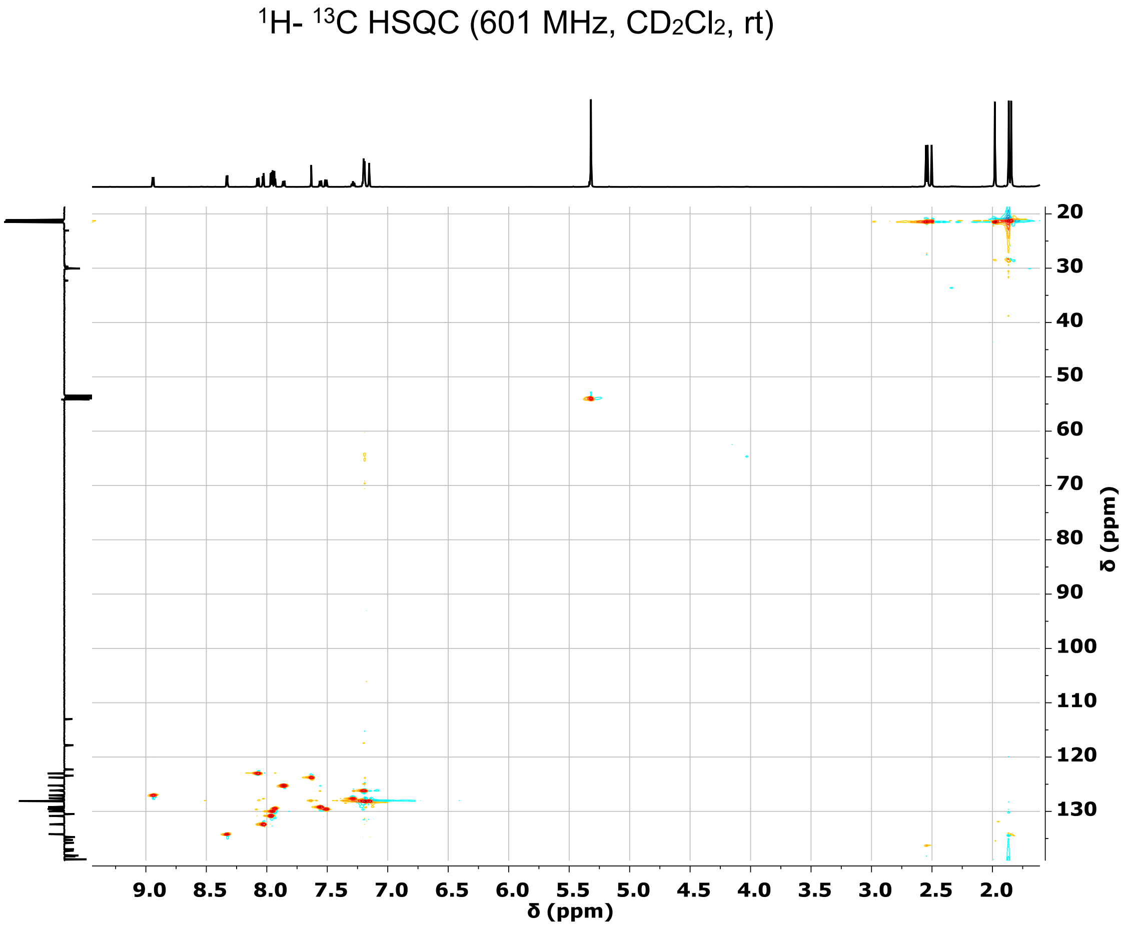

Figure S9. ^{1}H- ^{13}C HSQC of **PorNaph**.

^1H- ^{13}C HMBC (601 MHz, CD_2Cl_2, rt)

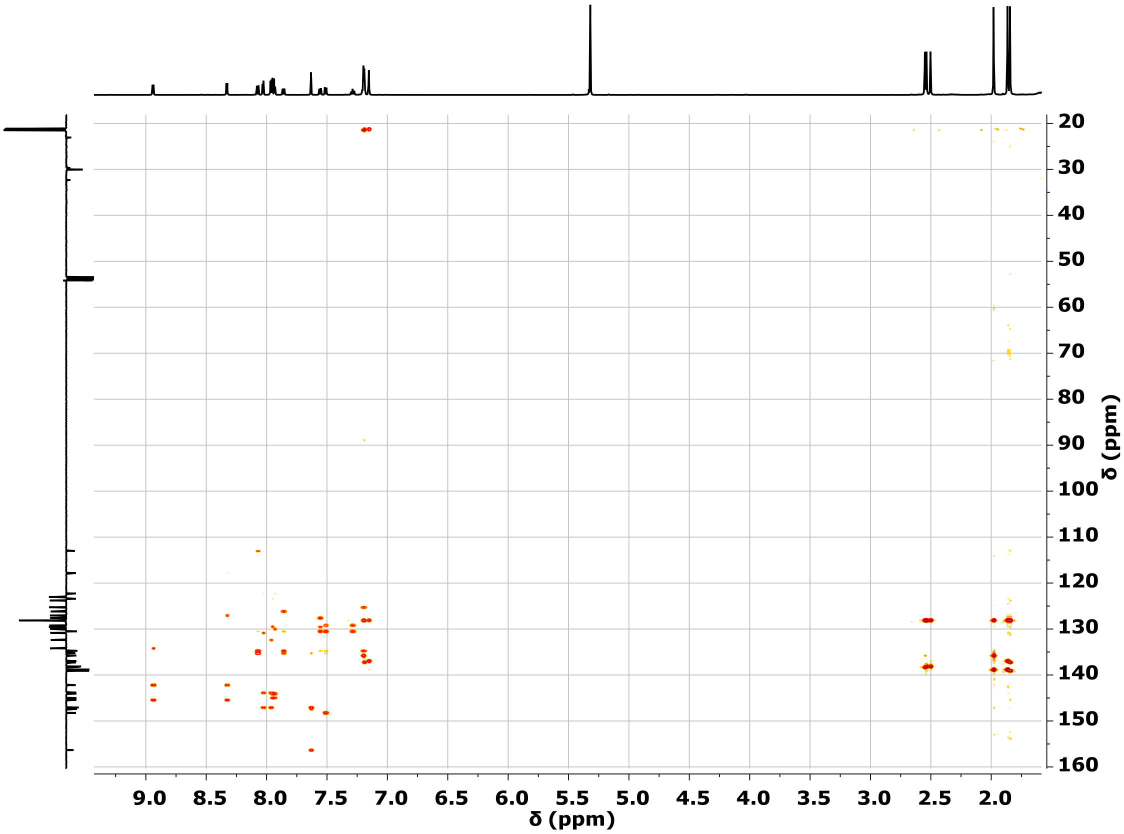

Figure S10. ^1H- ^{13}C HMBC of **PorNaph**.

MS (MALDI)

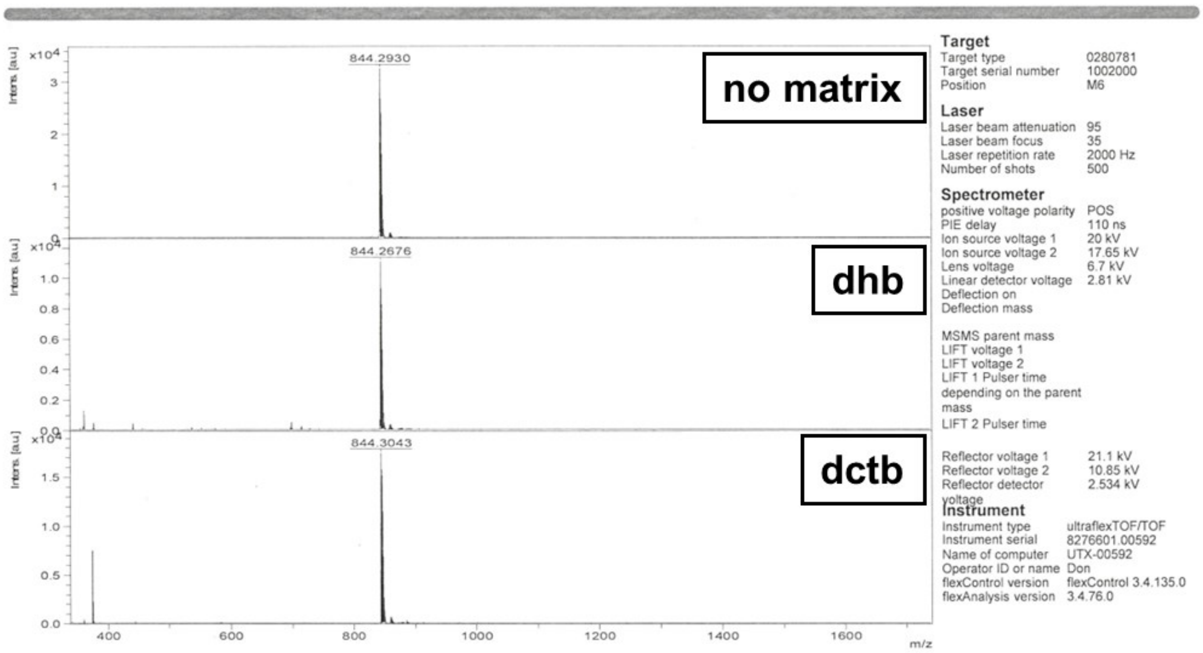

HRMS (MALDI)

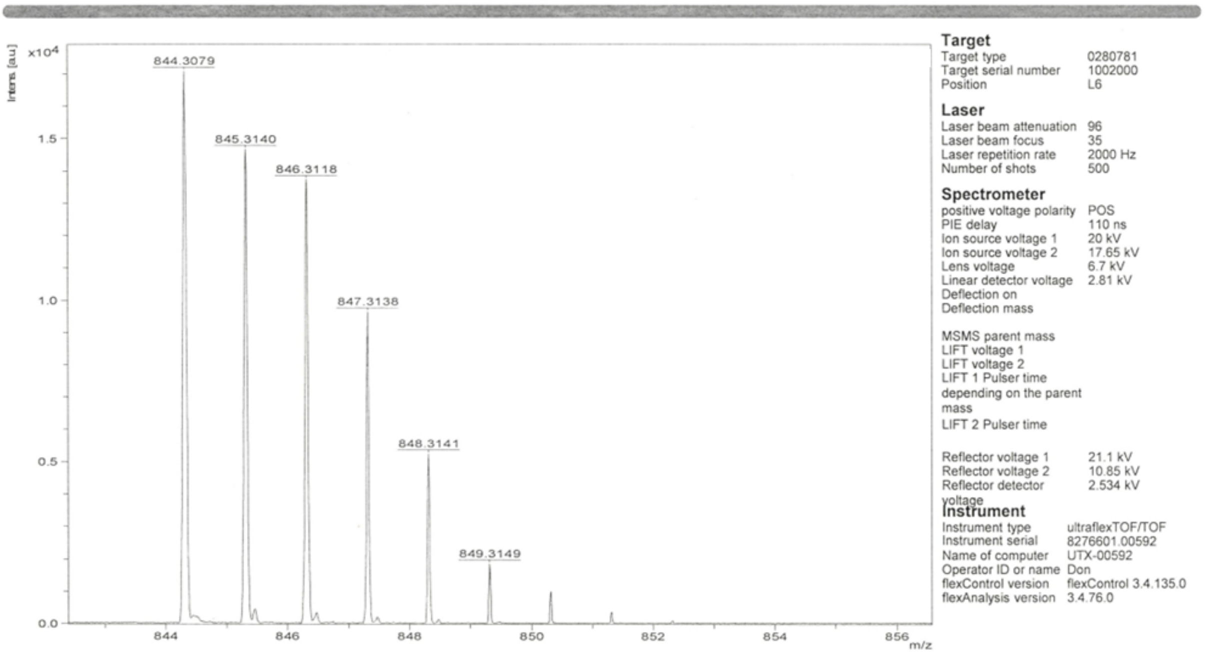

SmartFormula

Formula	Mass	Error	mSigma	DblEq	N rule	Electron Configuration
C 57 H 46 N 4 Ni	844.3070	0.9743	156.5882	37.00	ok	odd

Figure S11. MS/HRMS (MALDI) of **PorNaph**.

^{1}H NMR (400 MHz, $CDCl_3$, rt)

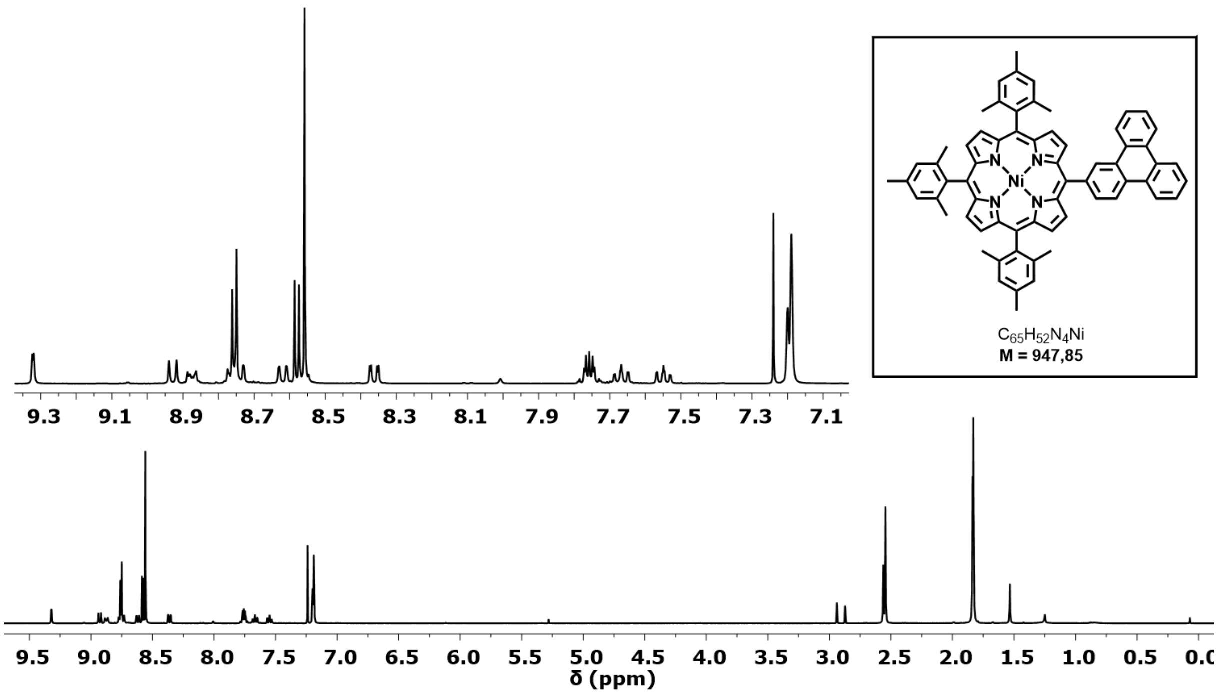

^{13}C NMR (101 MHz, $CDCl_3$, rt)

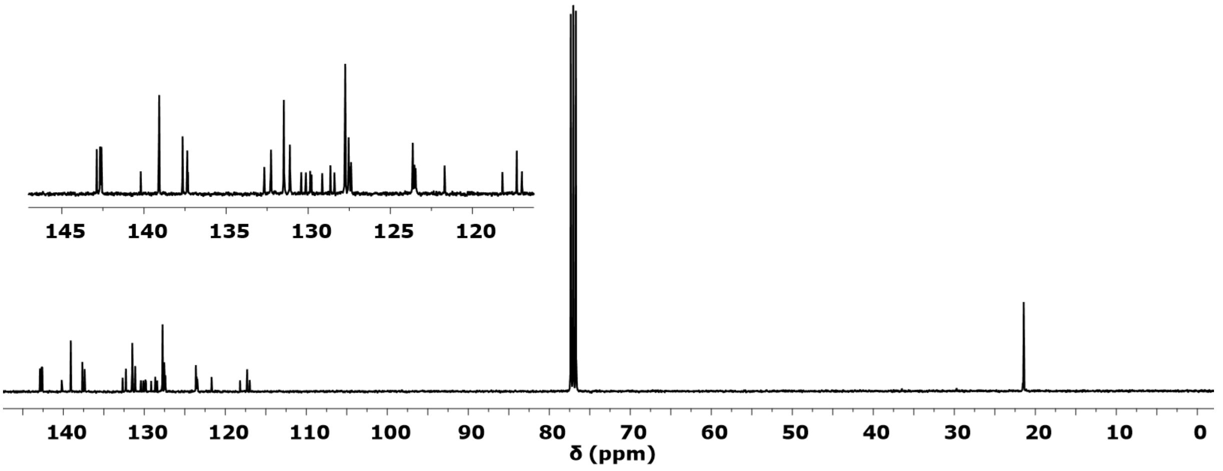

Figure S12. ^{1}H and ^{13}C NMR of **8**.

MS (MALDI)

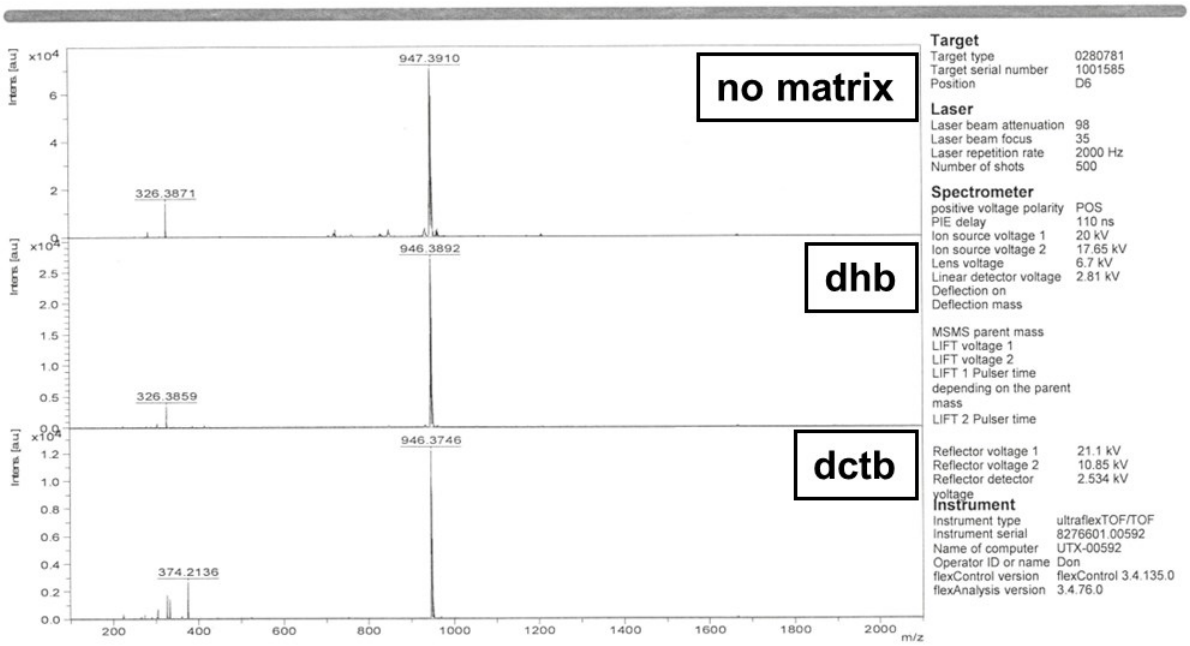

HRMS (MALDI)

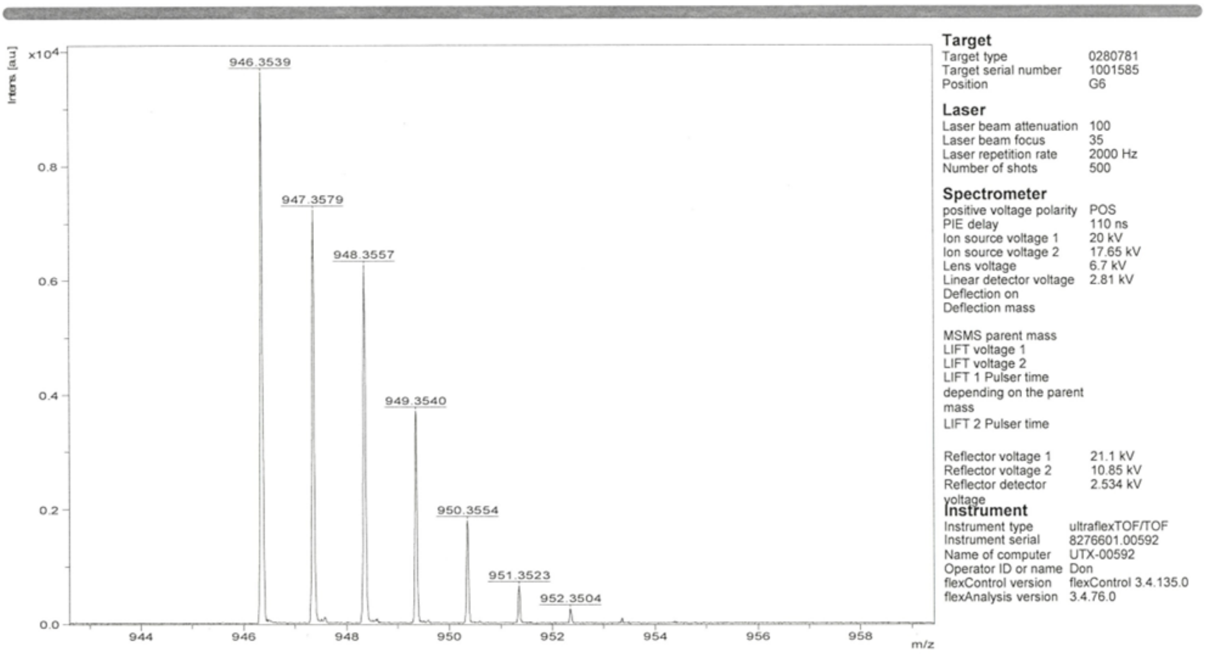

SmartFormula

Formula	Mass	Error	mSigma	DblEq	N rule	Electron Configuration
C 65 H 52 N 4 Ni	946.3540	0.0928	29.2681	42.00	ok	odd

Figure S13. MS/HRMS (MALDI) of **8**.

^{1}H NMR (601 MHz, CD_2Cl_2, rt)

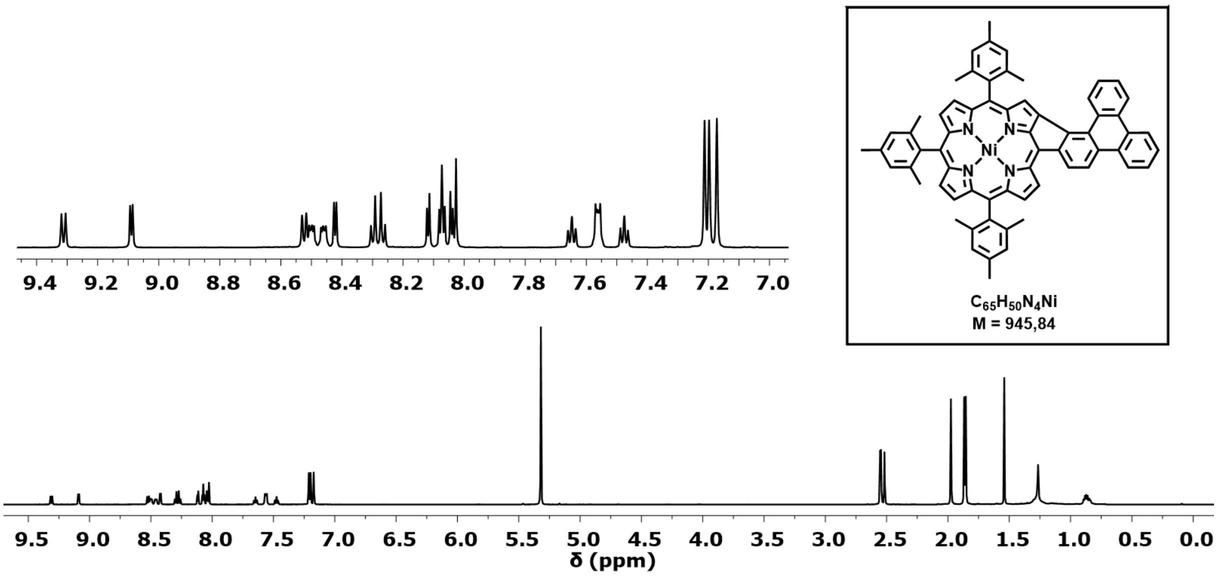

^{13}C NMR - DEPTQ135 (151 MHz, CD_2Cl_2, rt)

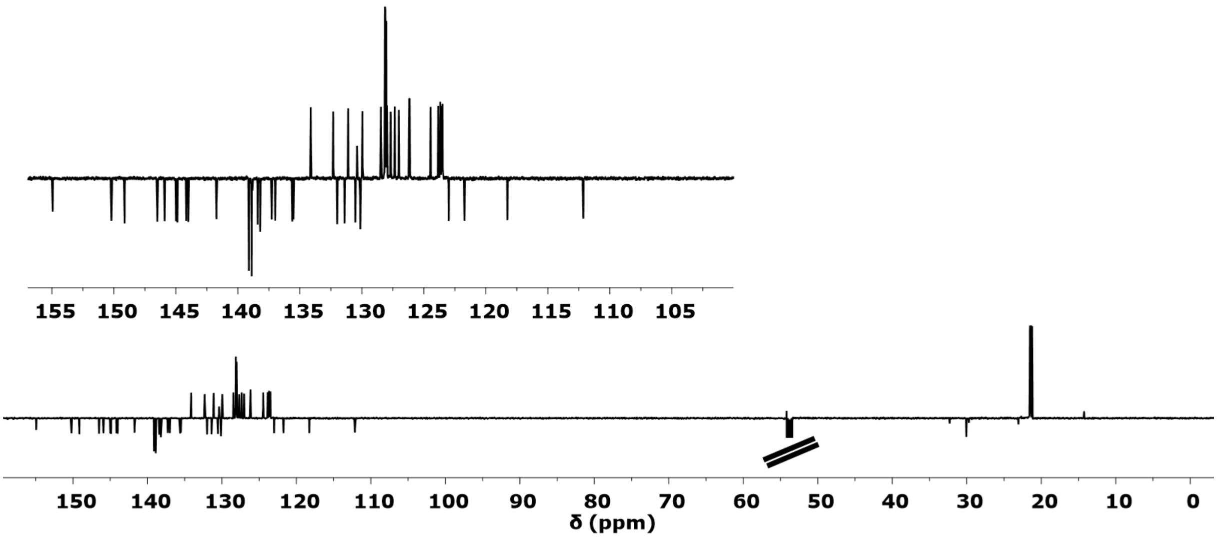

Figure S14. ^{1}H and ^{13}C NMR (DEPTQ135) of **PorTrip**.

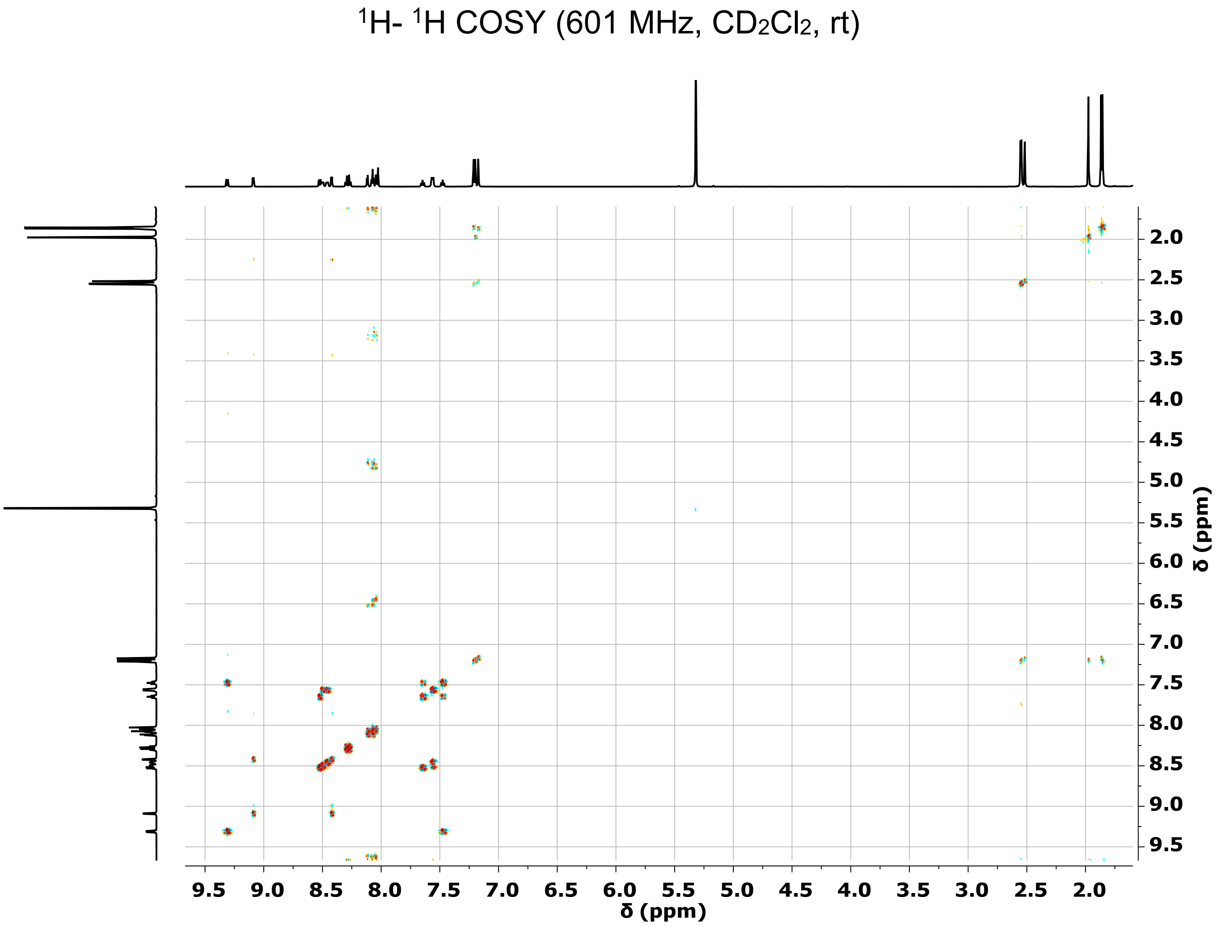

Figure S15. ^{1}H- ^{1}H COSY of **PorTrip**.

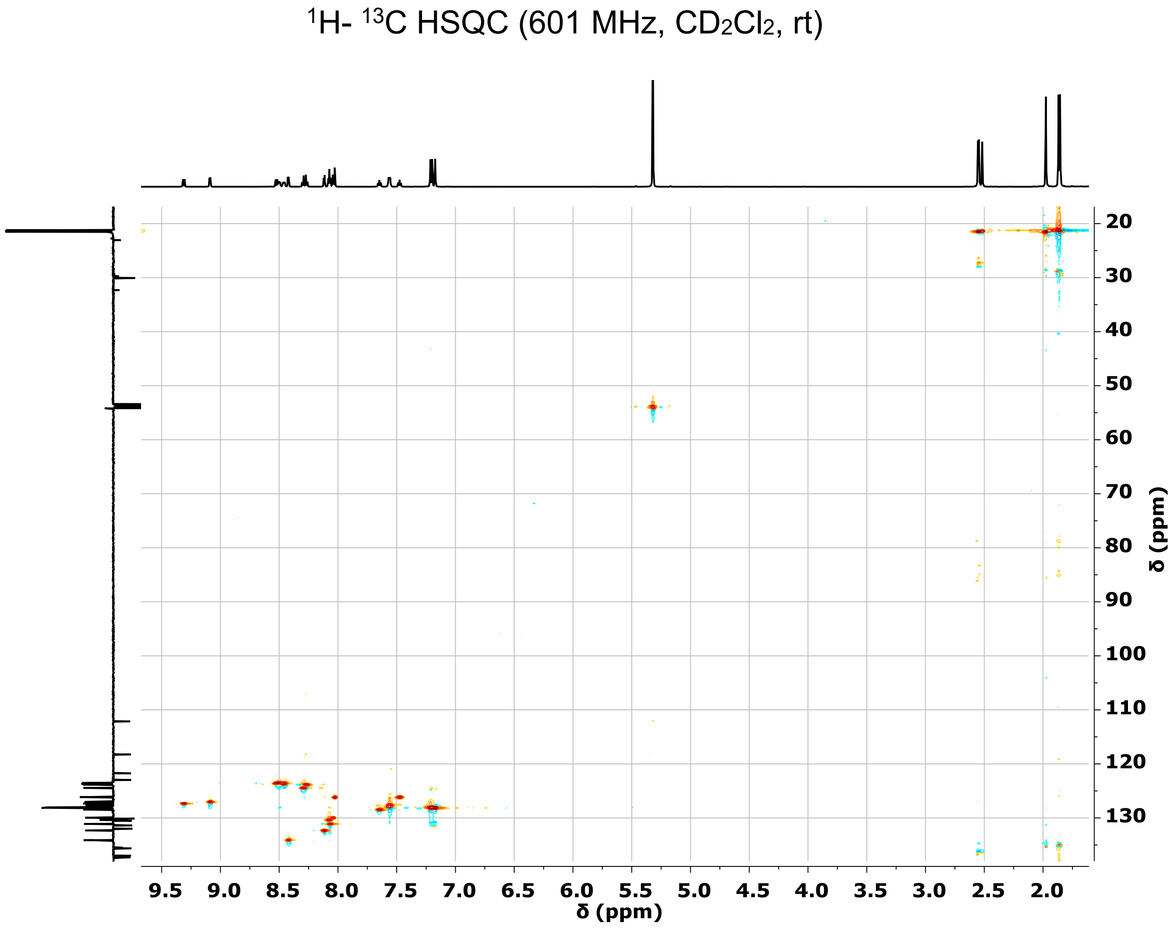

Figure S16. ^{1}H- ^{13}C HSQC of **PorTrip**.

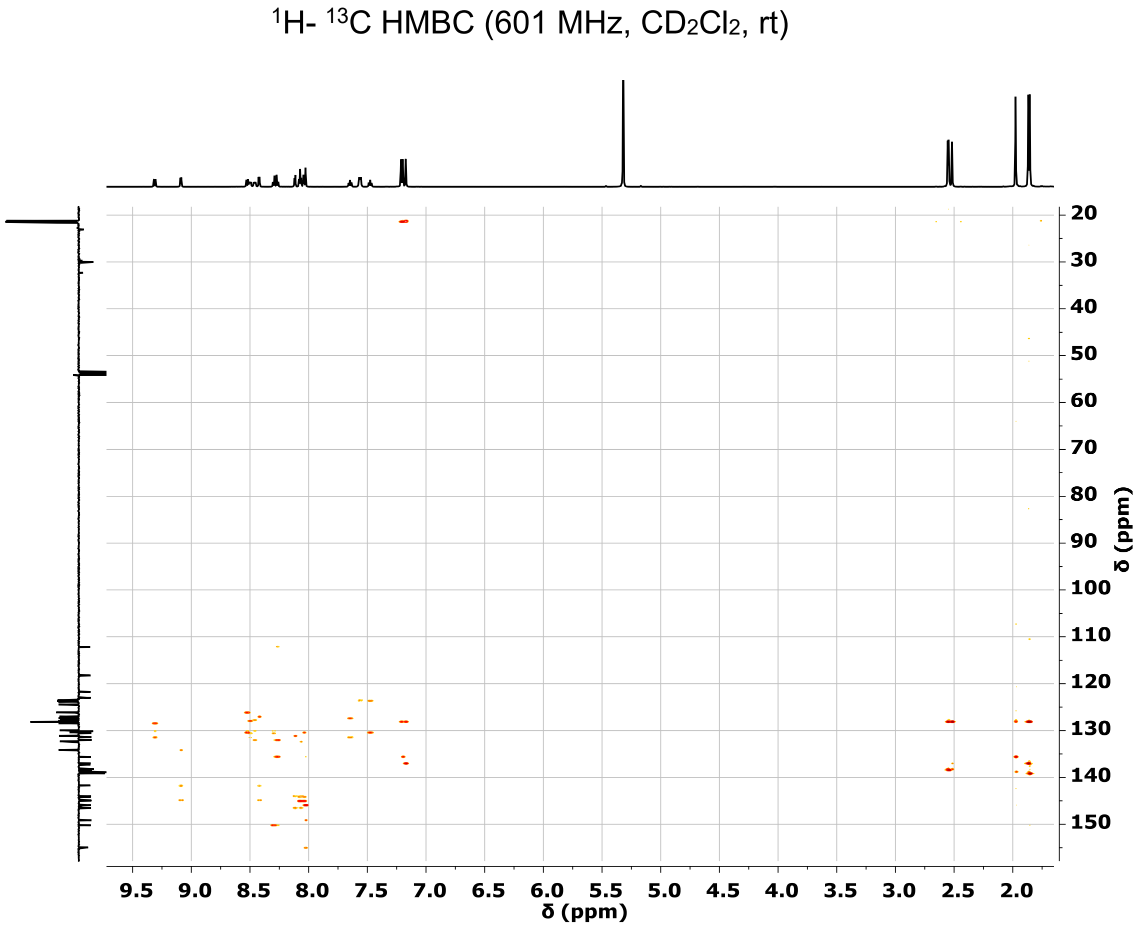

Figure S17. ^{1}H- ^{13}C HMBC of **PorTrip**.

MS (MALDI)

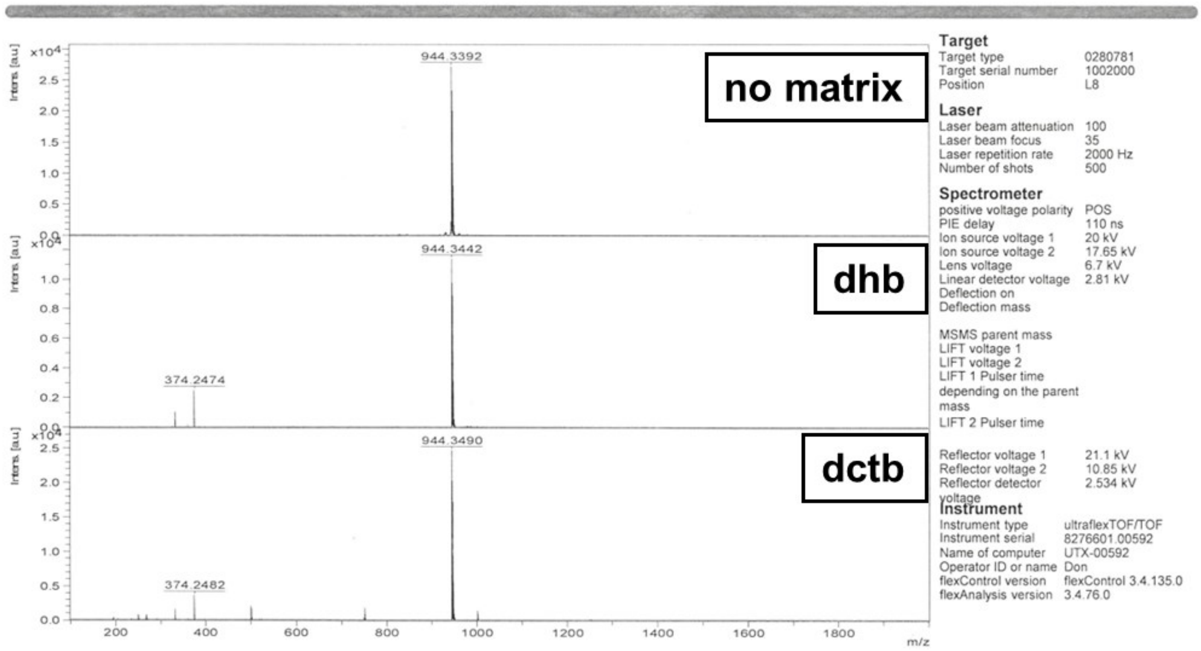

HRMS (MALDI)

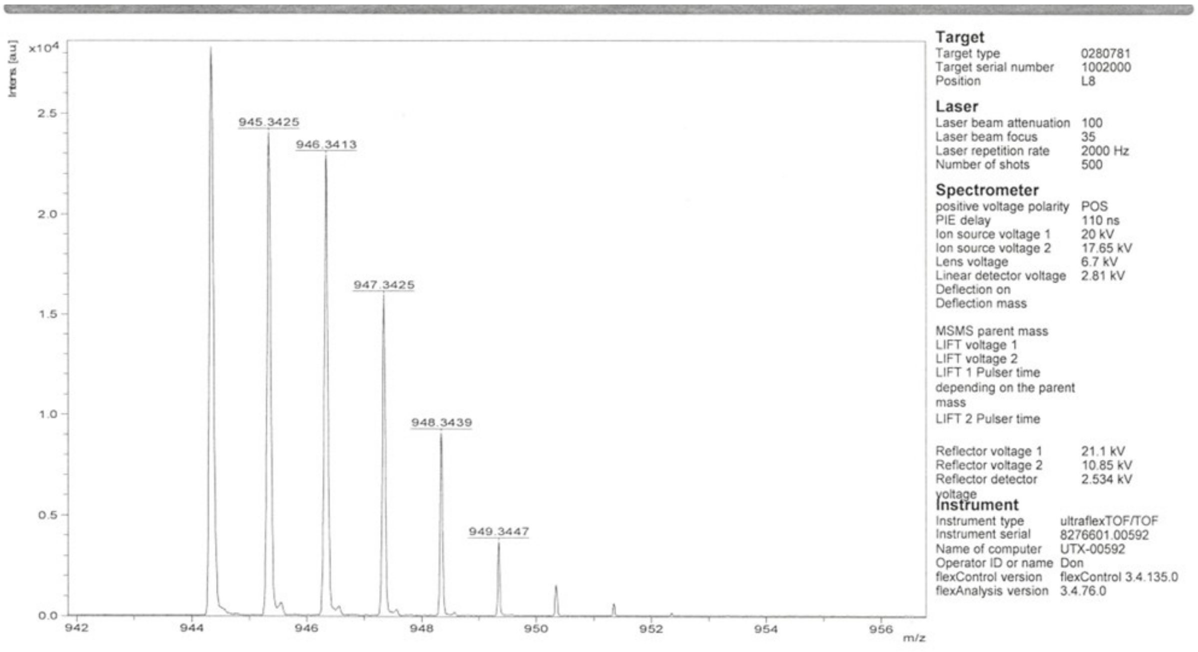

SmartFormula

Formula	Mass	Error	mSigma	DblEq	N rule	Electron Configuration
C 65 H 50 N 4 Ni	944.3383	0.1704	112.6625	43.00	ok	odd

Figure S18. MS/HRMS (MALDI) of **PorTrip**.

^{1}H NMR (400 MHz, $CDCl_3$, rt)

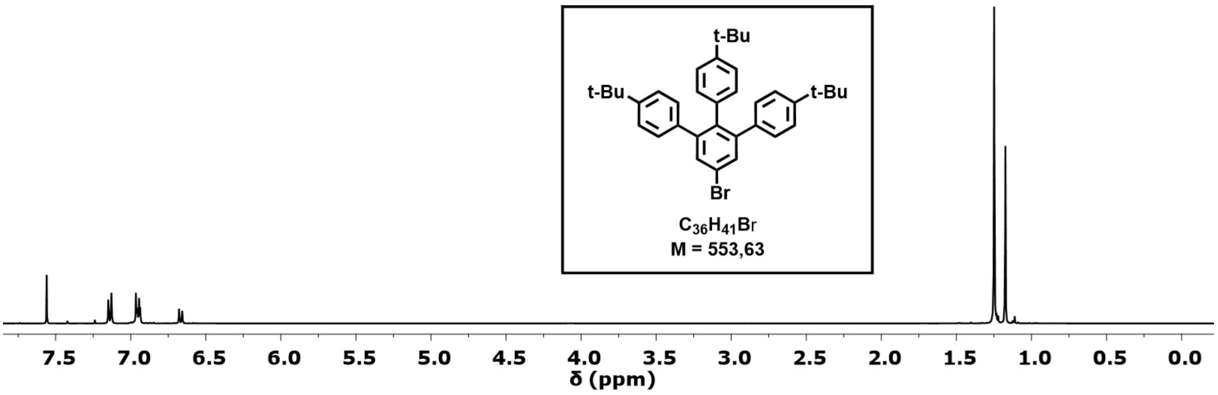

^{13}C NMR (101 MHz, $CDCl_3$, rt)

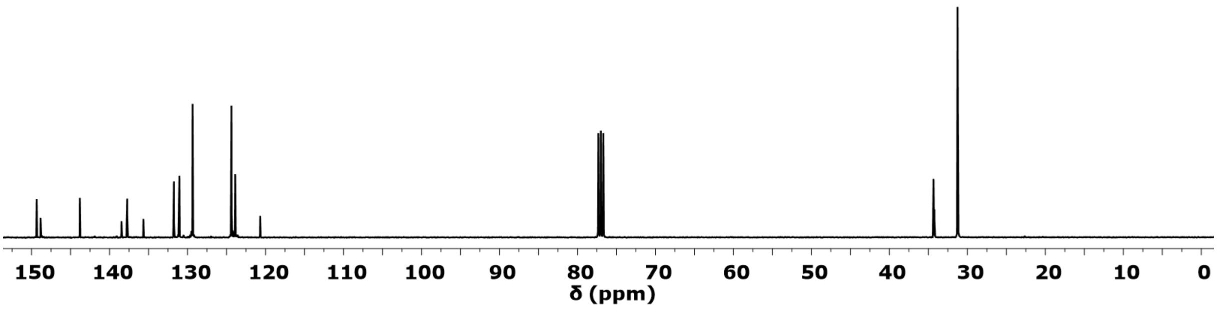

Figure S19. ^{1}H and ^{13}C NMR of **15**.

MS (APPI)

Acquisition Parameter

Source Type	APPI	Ion Polarity	Positive	Set Nebulizer	5.2 Bar		
Focus	Not active	Set Capillary	700 V	Set Dry Heater	220 °C		
Scan Begin	80 m/z	Set End Plate Offset	-500 V	Set Dry Gas	1.2 l/min		
Scan End	1550 m/z	Set Charging Voltage	0 V	Set Divert Valve	Waste		
		Set Corona	0 nA	Set APCI Heater	250 °C		

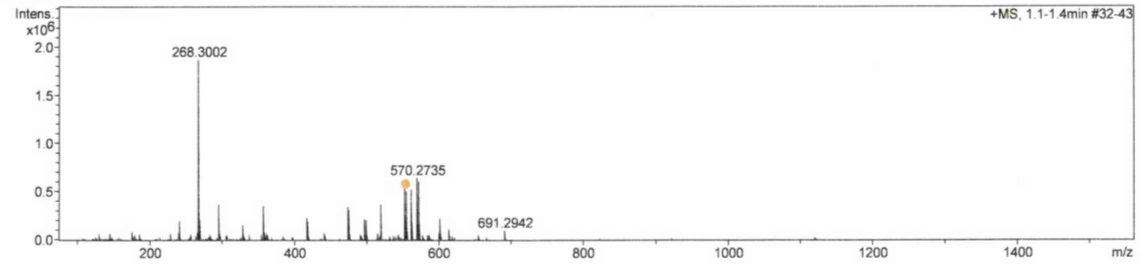

HRMS (APPI)

Acquisition Parameter

Source Type	APPI	Ion Polarity	Positive	Set Nebulizer	5.2 Bar
Focus	Not active	Set Capillary	700 V	Set Dry Heater	220 °C
Scan Begin	80 m/z	Set End Plate Offset	-500 V	Set Dry Gas	1.2 l/min
Scan End	1550 m/z	Set Charging Voltage	0 V	Set Divert Valve	Waste
		Set Corona	0 nA	Set APCI Heater	250 °C

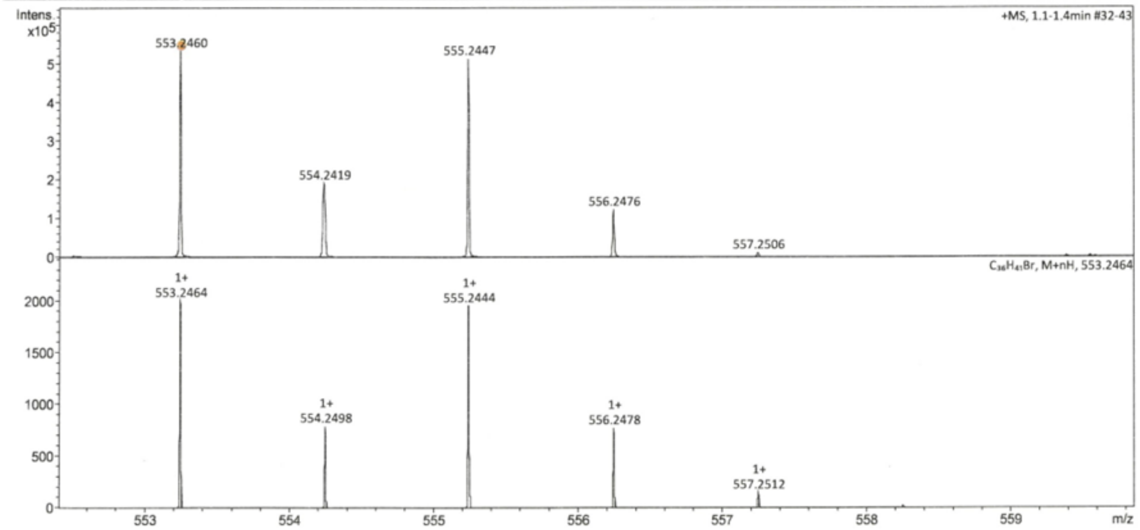

Meas. m/z	#	Ion Formula	m/z	err [ppm]	mSigma	# mSigma	Score	rdb	e⁻ Conf	N-Rule
553.2460	1	C36H42Br	553.2464	0.8	73.7	1	100.00	15.5	even	ok

Figure S20. MS/HRMS (APPI) of **15**.

^{1}H NMR (400 MHz, $CDCl_3$, rt)

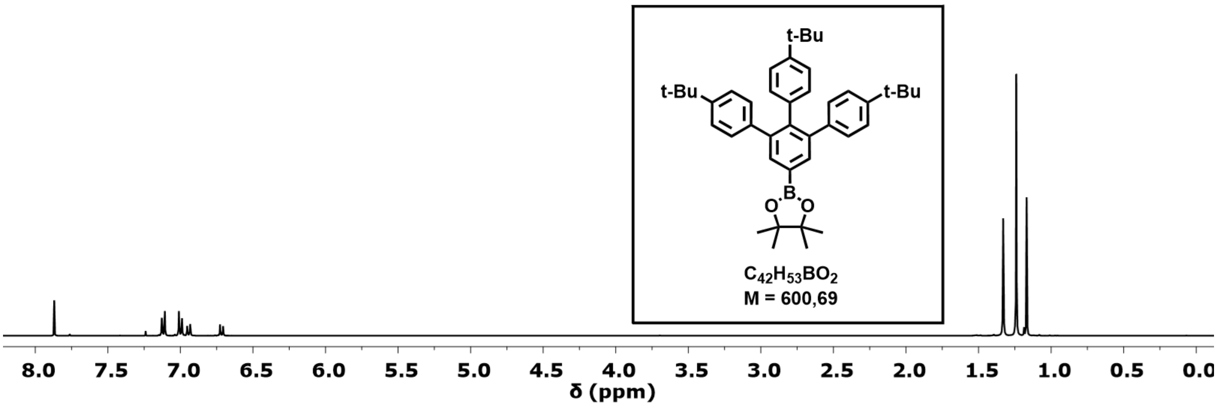

^{13}C NMR (101 MHz, $CDCl_3$, rt)

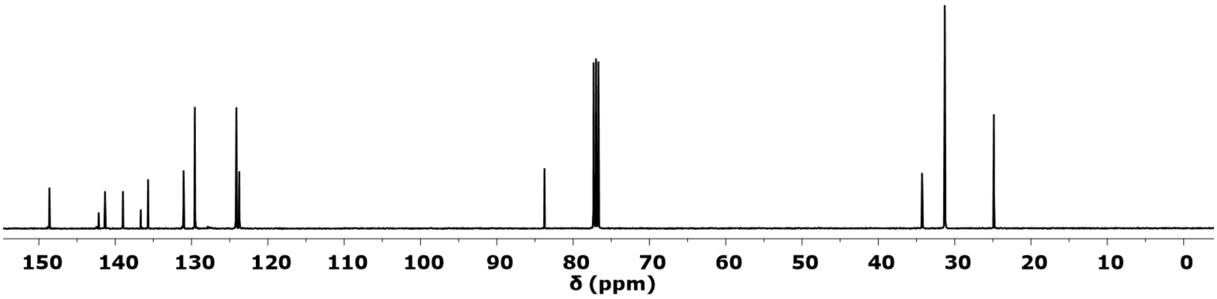

Figure S21. ^{1}H and ^{13}C NMR of **9**.

MS (APPI)

Acquisition Parameter

Source Type	APPI	Ion Polarity	Positive	Set Nebulizer	5.2 Bar
Focus	Not active	Set Capillary	700 V	Set Dry Heater	220 °C
Scan Begin	80 m/z	Set End Plate Offset	-500 V	Set Dry Gas	1.2 l/min
Scan End	1550 m/z	Set Charging Voltage	0 V	Set Divert Valve	Waste
		Set Corona	0 nA	Set APCI Heater	250 °C

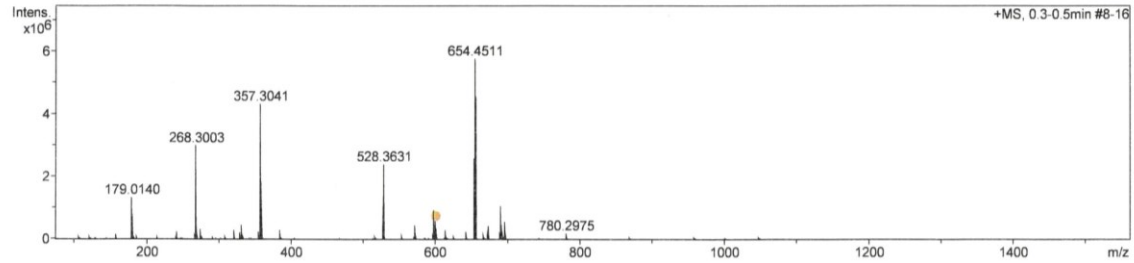

HRMS (APPI)

Acquisition Parameter

Source Type	APPI	Ion Polarity	Positive	Set Nebulizer	5.2 Bar
Focus	Not active	Set Capillary	700 V	Set Dry Heater	220 °C
Scan Begin	80 m/z	Set End Plate Offset	-500 V	Set Dry Gas	1.2 l/min
Scan End	1550 m/z	Set Charging Voltage	0 V	Set Divert Valve	Waste
		Set Corona	0 nA	Set APCI Heater	250 °C

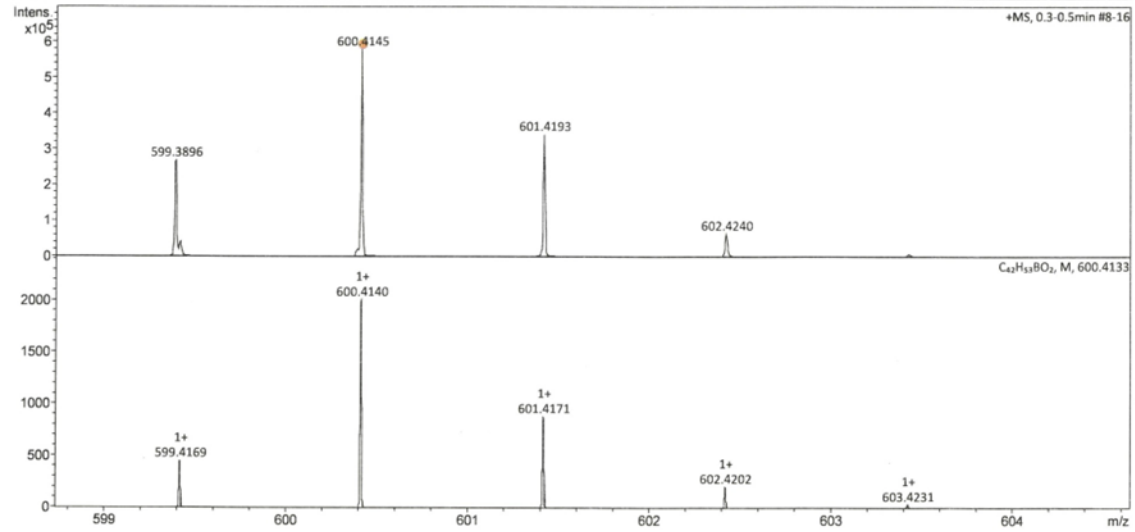

Meas. m/z	#	Ion Formula	m/z	err [ppm]	mSigma	# mSigma	Score	rdb	e⁻ Conf	N-Rule
600.4145	1	C42H53BO2	600.4133	-0.8	121.0	1	100.00	17.0	odd	ok

Figure S22. MS/HRMS (APPI) of **9**.

^{1}H NMR (400 MHz, CD_2Cl_2, rt)

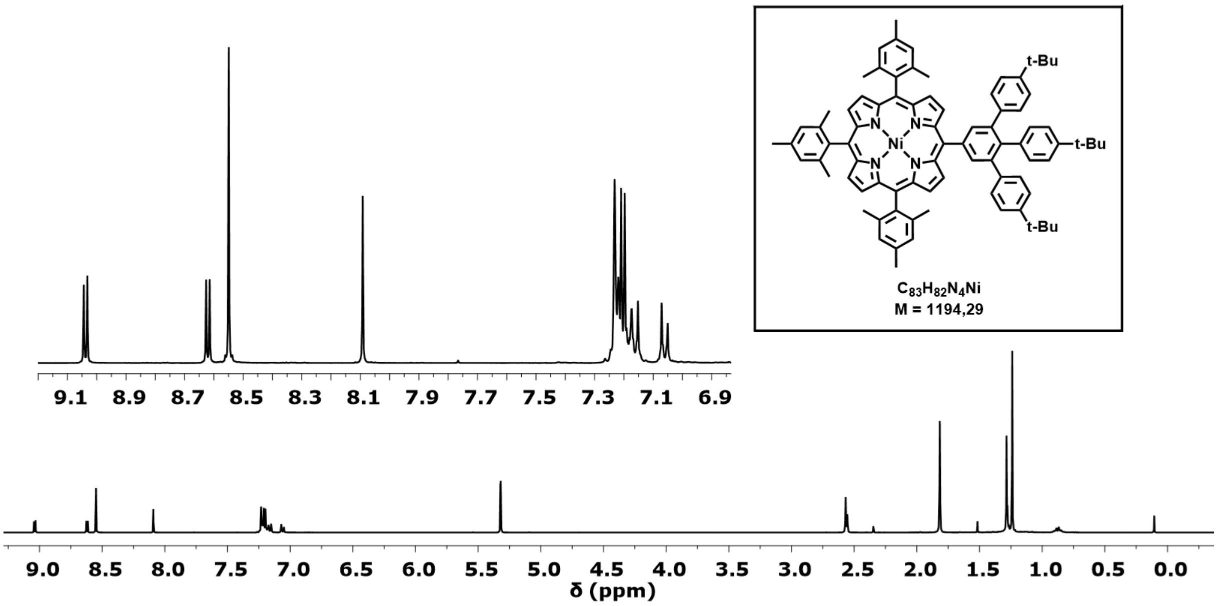

^{13}C NMR (101 MHz, CD_2Cl_2, rt)

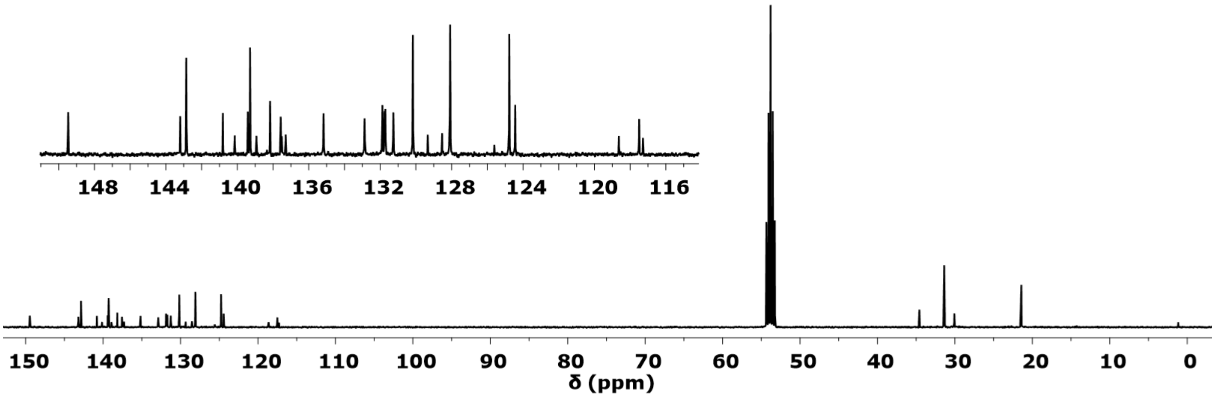

Figure S23. ^{1}H and ^{13}C NMR of **10**.

MS (MALDI)

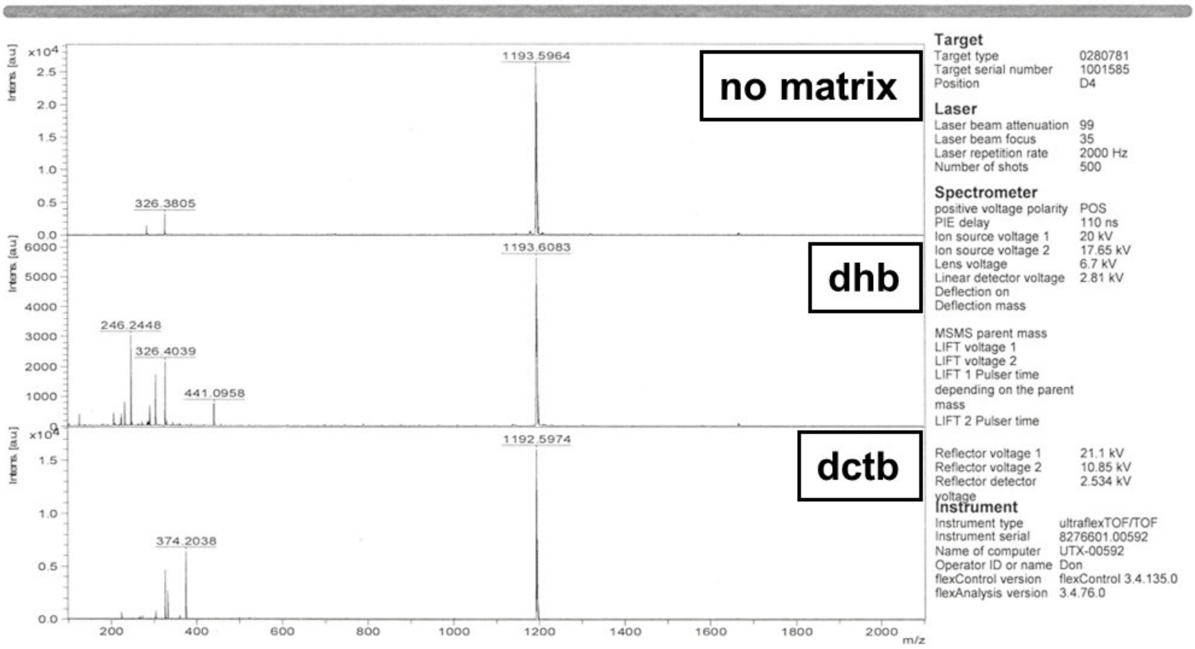

HRMS (MALDI)

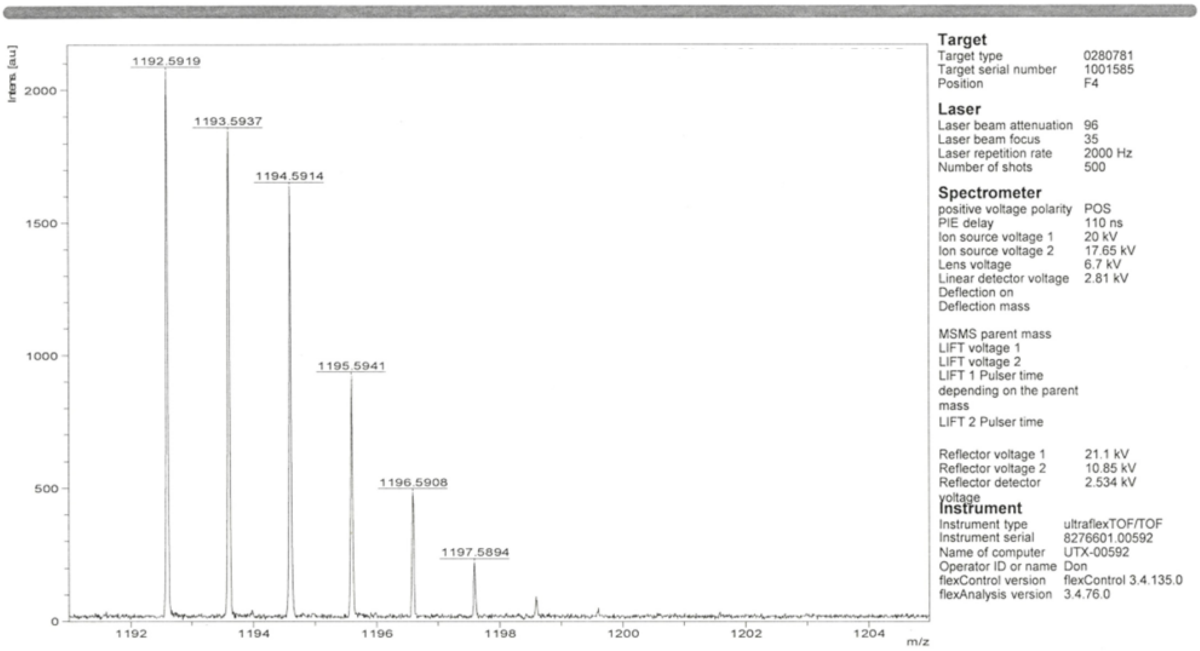

SmartFormula

Formula	Mass	Error	mSigma	DblEq	N rule	Electron Configuration
C 83 H 82 N 4 Ni	1,192.5887	2.6147	29.1103	45.00	ok	odd

Figure S24. MS/HRMS (MALDI) of **10**.

^{1}H NMR (400 MHz, $CDCl_3$, rt)

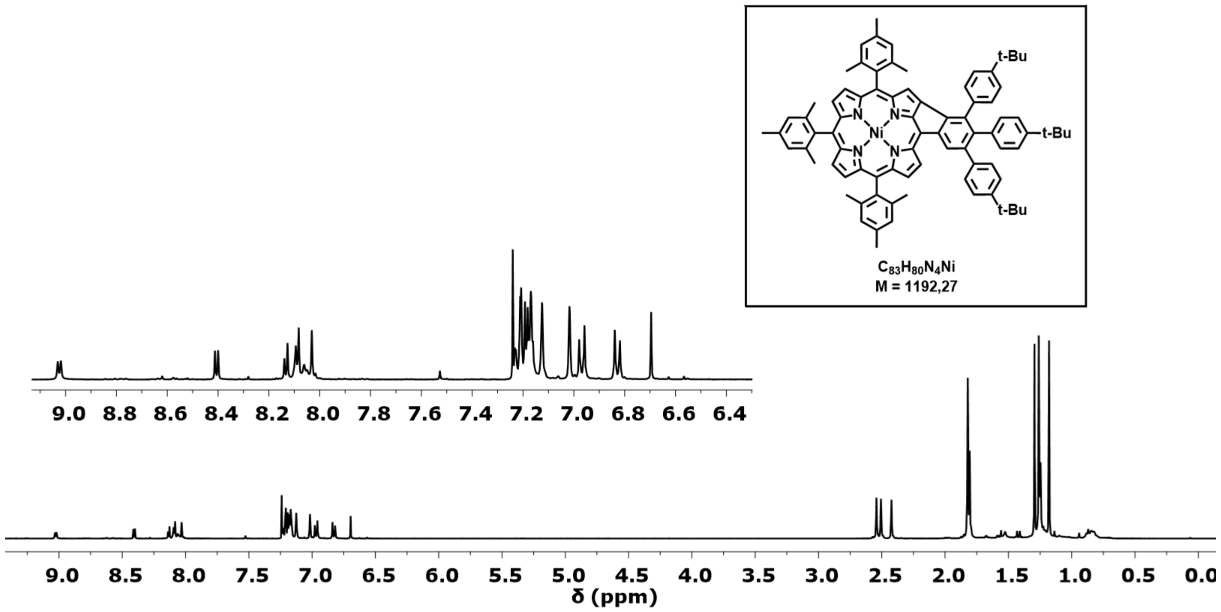

^{13}C NMR (101 MHz, $CDCl_3$, rt)

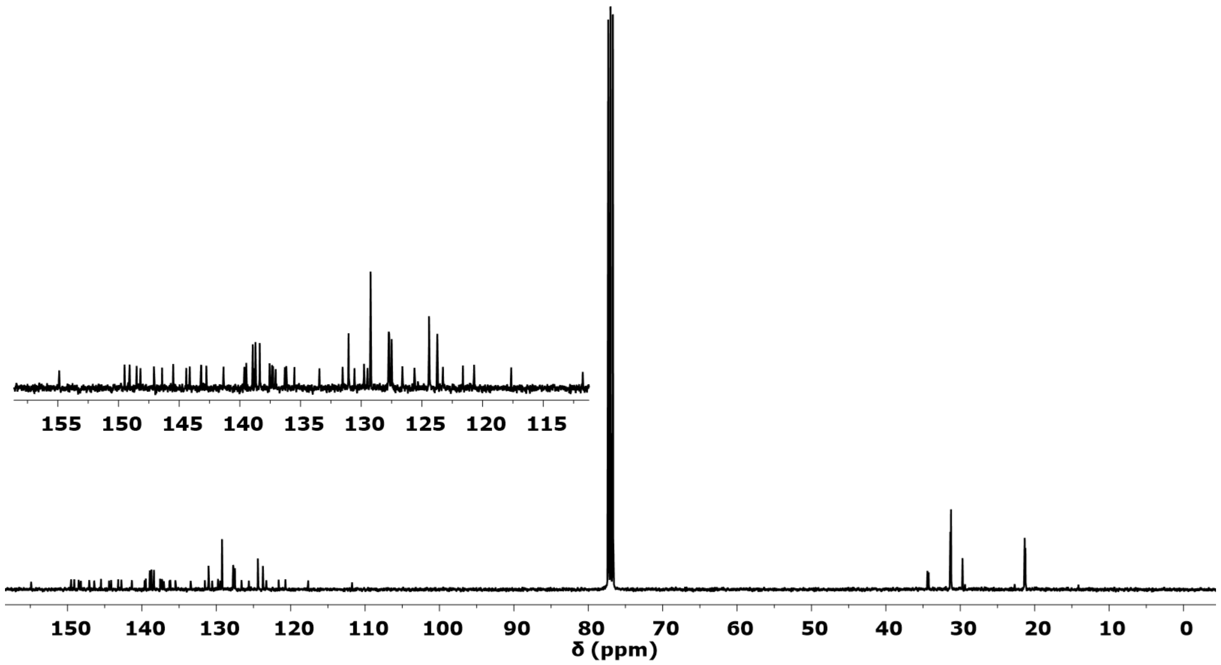

Figure S25. ^{1}H and ^{13}C NMR of **11**.

MS (MALDI)

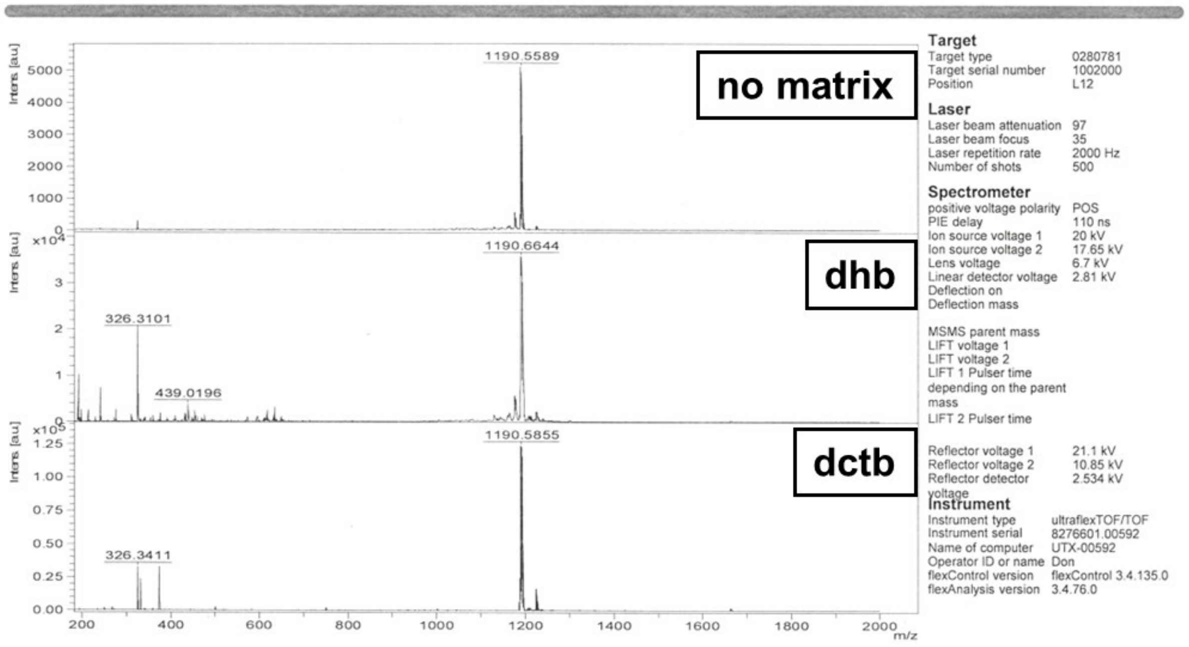

HRMS (MALDI)

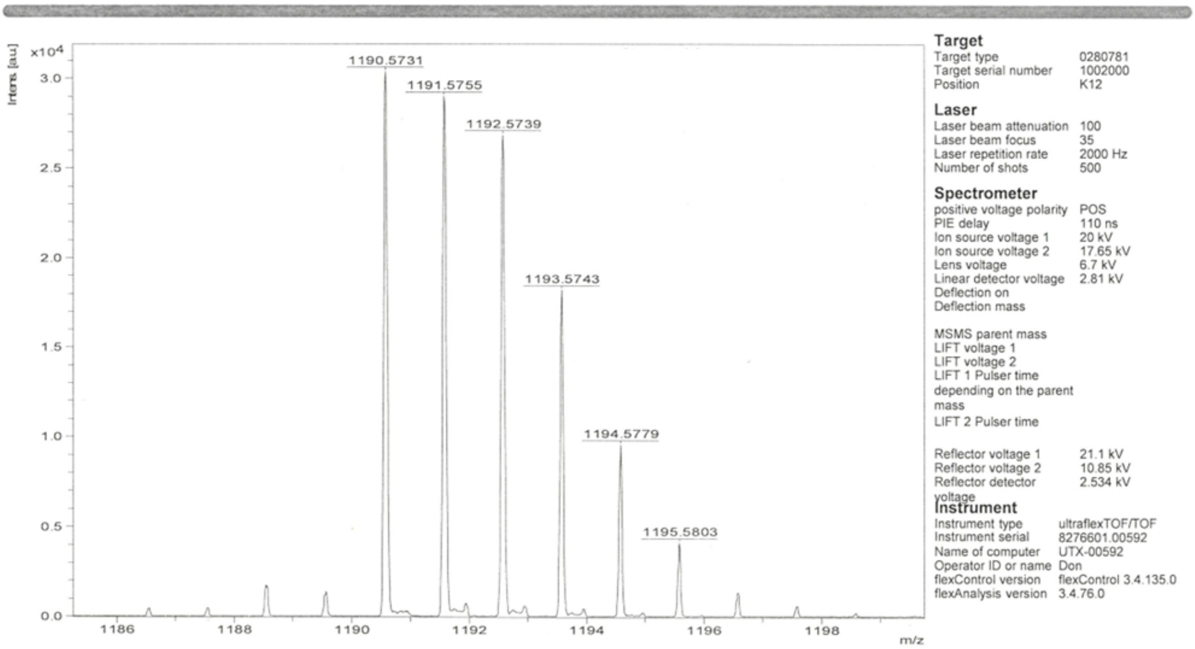

SmartFormula

Formula	Mass	Error	mSigma	DblEq	N rule	Electron Configuration
C 83 H 80 N 4 Ni	1,190.5731	0.0259	52.0968	46.00	ok	odd

Figure S26. MS/HRMS (MALDI) of **11**.

^{1}H NMR (601 MHz, CD_2Cl_2, rt)

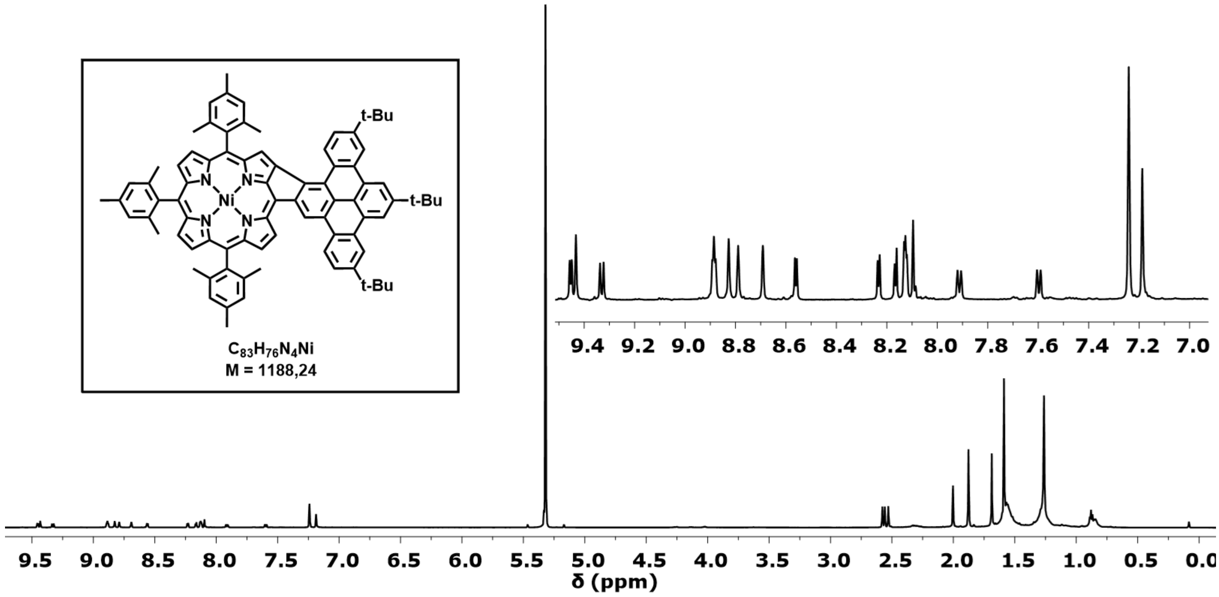

^{13}C NMR - DEPTQ135 (151 MHz, CD_2Cl_2, rt)

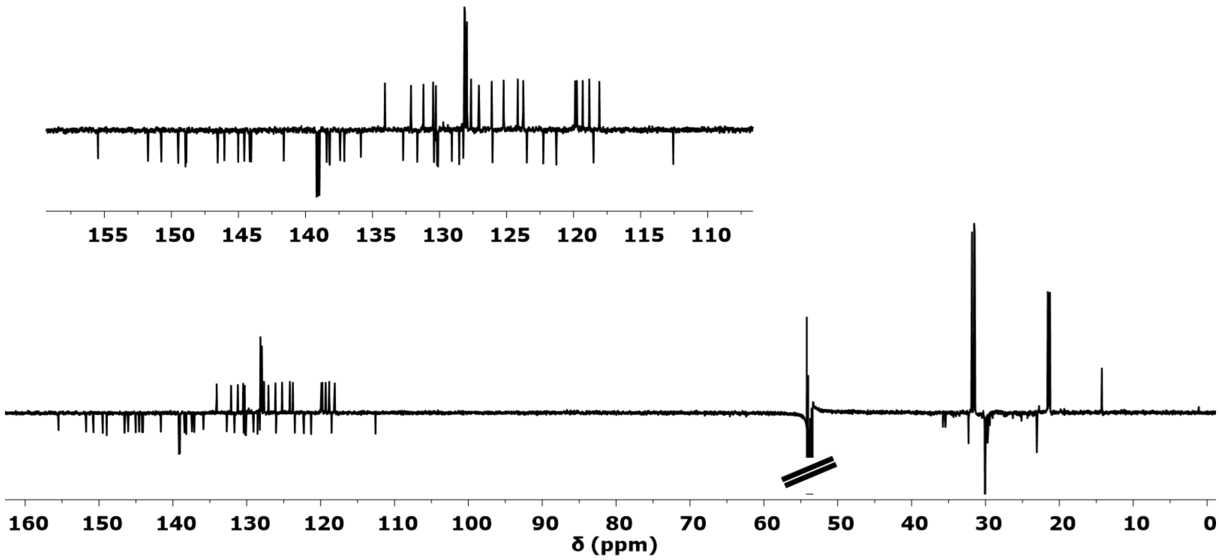

Figure S27. ^{1}H and ^{13}C NMR (DEPTQ135) of **PorDbtc**.

^{1}H- ^{1}H COSY (601 MHz, CD_2Cl_2, rt)

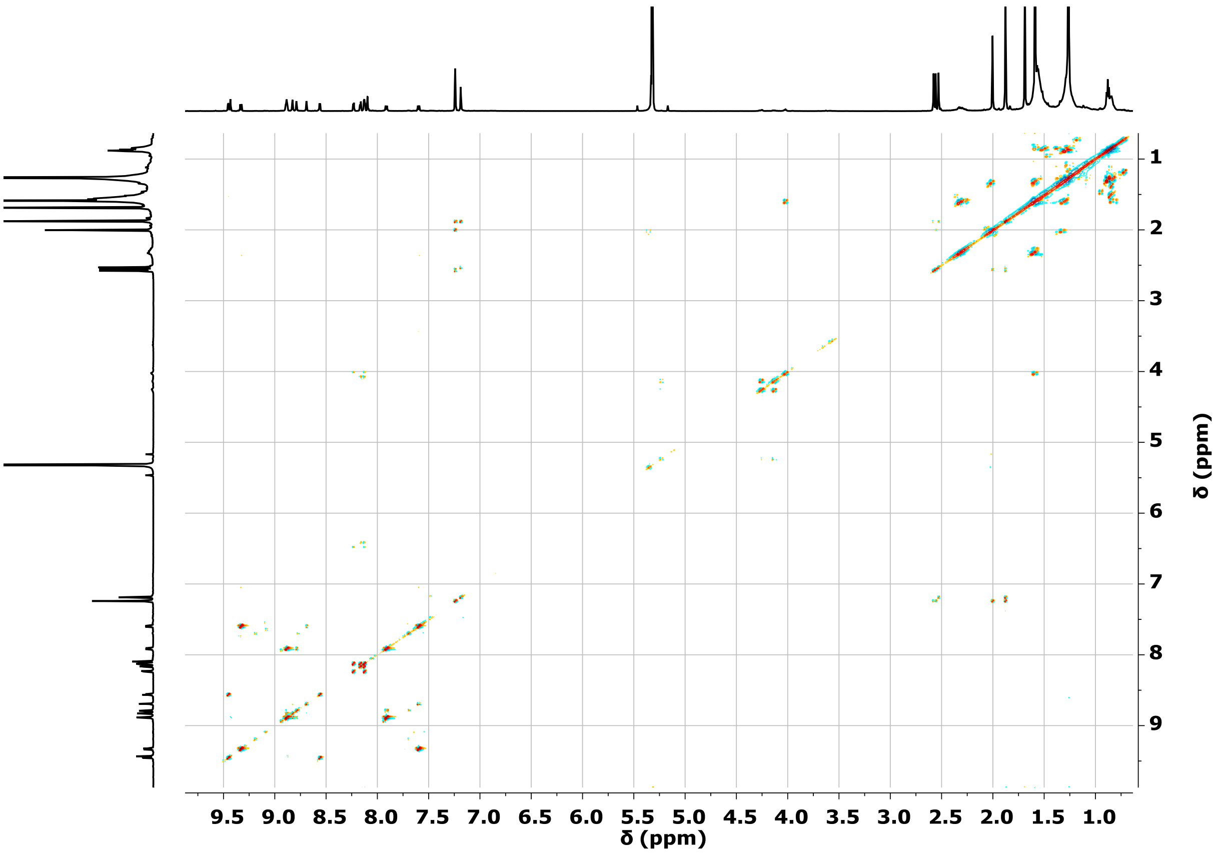

Figure S28. ^{1}H- ^{1}H COSY of **PorDbtc**.

^{1}H- ^{13}C HSQC (601 MHz, CD_2Cl_2, rt)

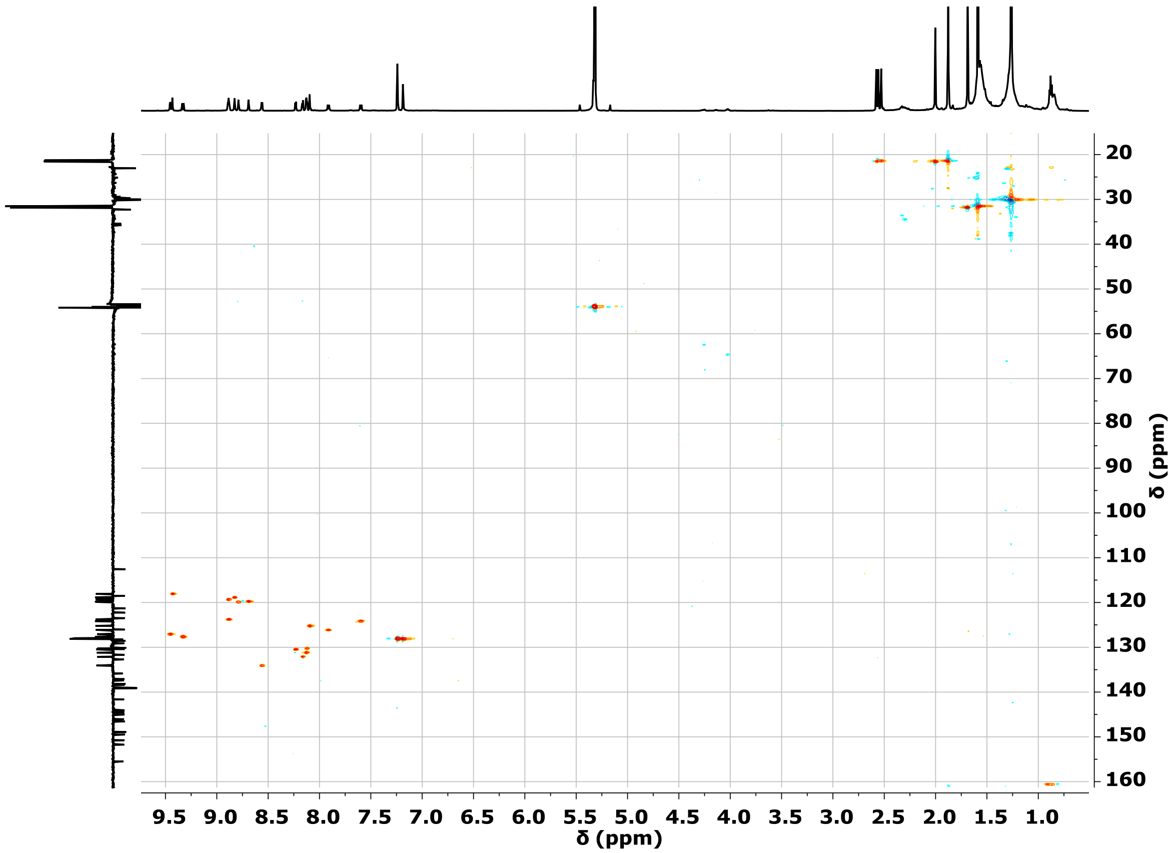

Figure S29. ^{1}H- ^{13}C HSQC of **PorDbtc**.

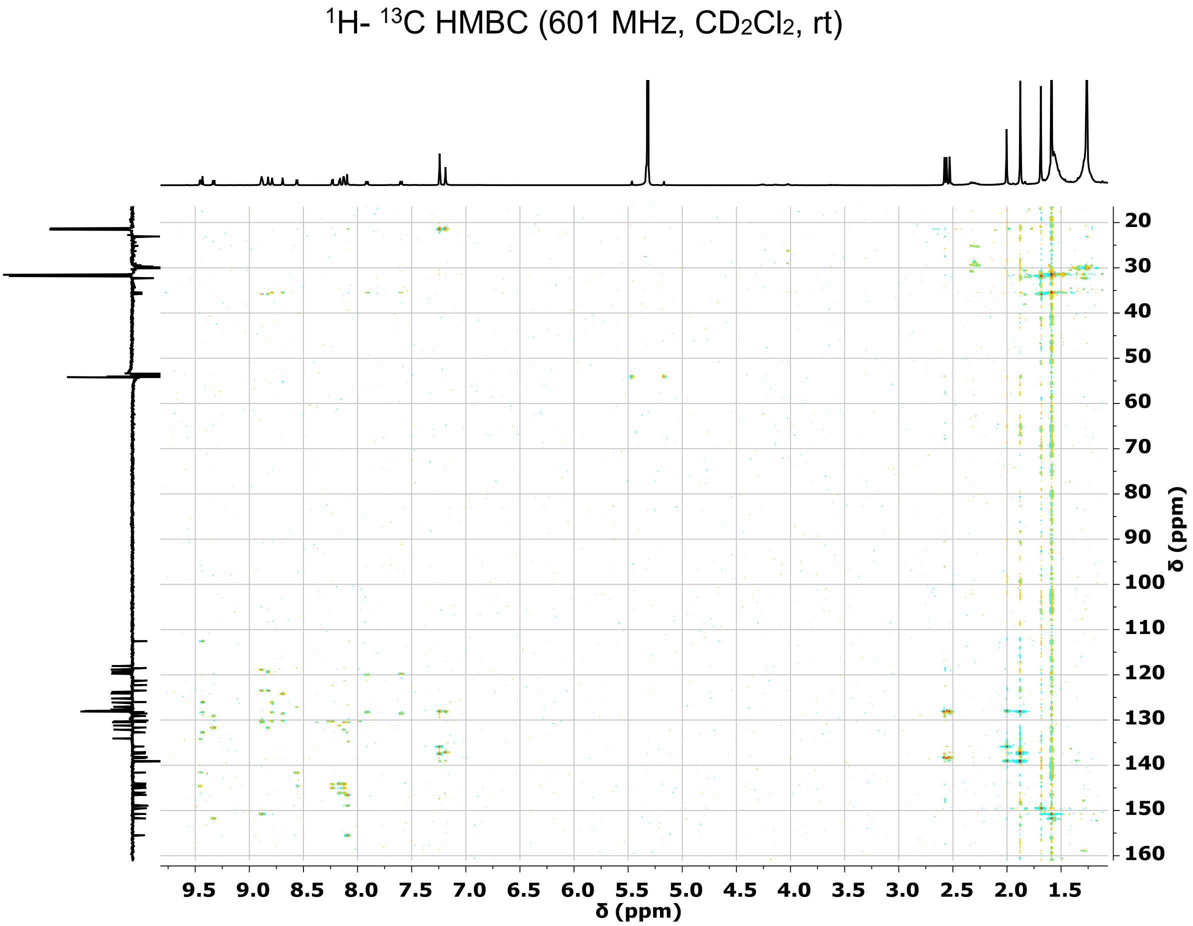

Figure S30. ^{1}H- ^{13}C HMBC of **PorDbtc**.

MS (MALDI)

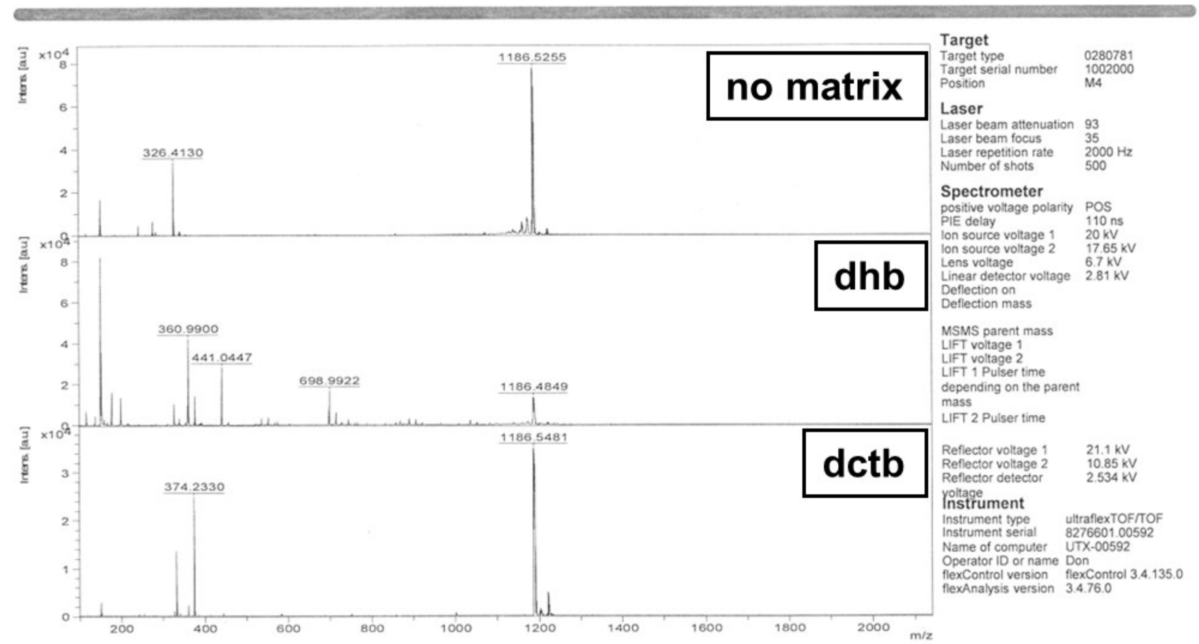

HRMS (MALDI)

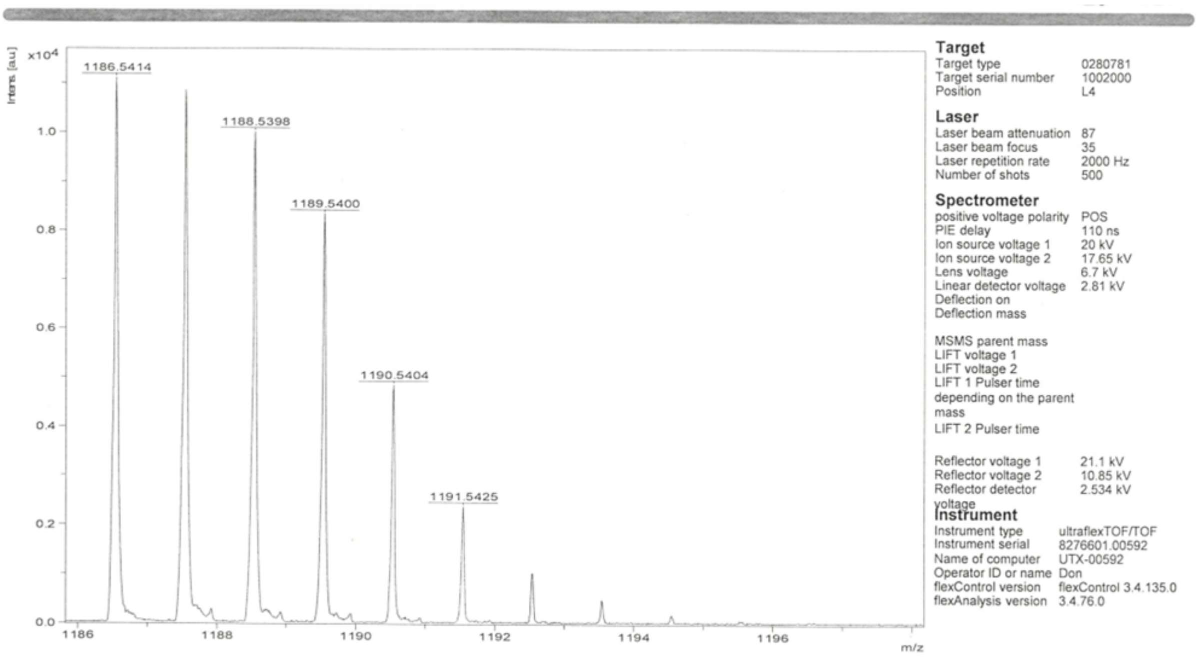

SmartFormula

Formula	Mass	Error	mSigma	DblEq	N rule	Electron Configuration
C 83 H 76 N 4 Ni	1,186.5418	0.3523	116.8344	48.00	ok	odd

Figure S31. MS/HRMS (MALDI) of **PorDbtc**.

^{1}H NMR Assignments of the Fused Porphyrins

Fused naphthalene-porphyrin PorNaph

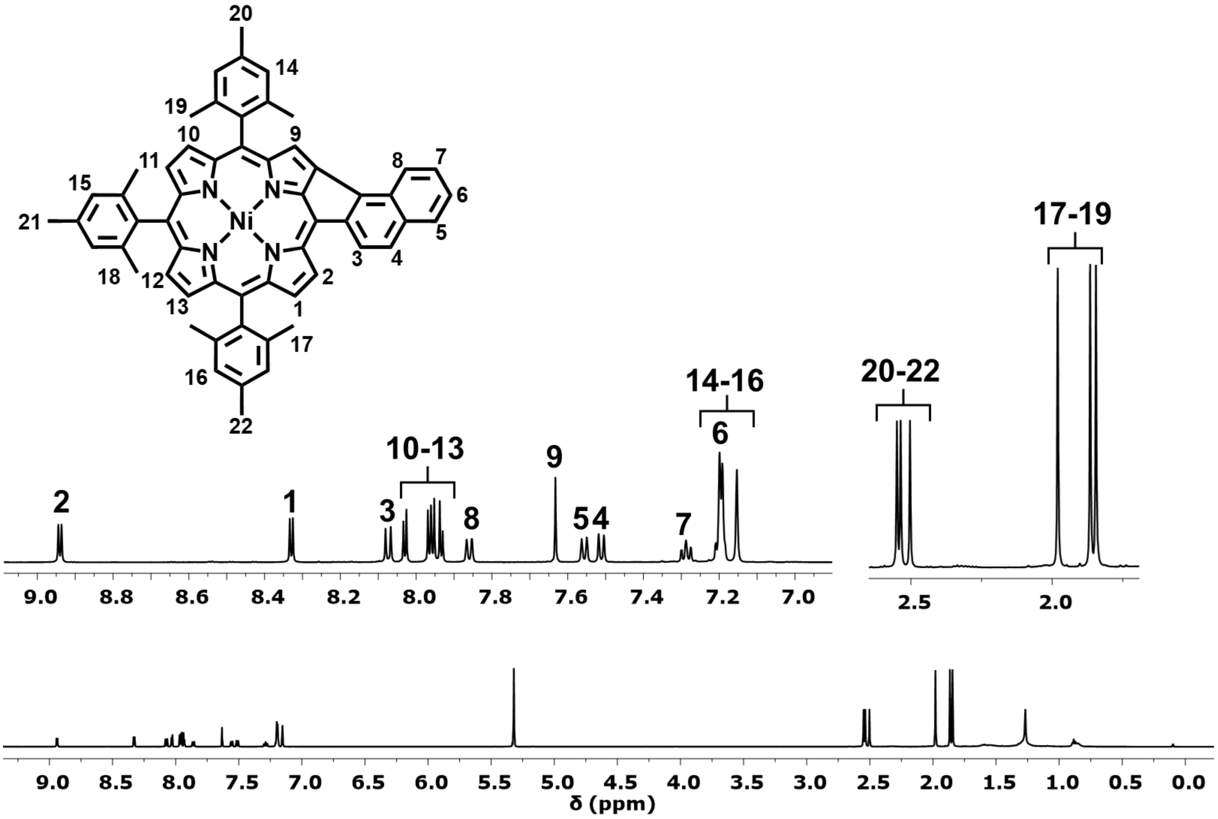

Figure S32. ^{1}H NMR of **PorNaph** with labeled protons.

^{1}H NMR (601 MHz, CD_2Cl_2, rt): δ [ppm]: 8.94 (d, *J* = 4.9 Hz, 1H), 8.33 (d, *J* = 4.9 Hz, 1H), 8.07 (d, *J* = 8.3 Hz, 1H), 8.03 (d, *J* = 4.8 Hz, 1H), 7.98-7.92 (m, 3H), 7.86 (d, *J* = 8.2 Hz, 1H), 7.63 (s, 1H), 7.57-7.54 (m, 1H), 7.51 (d, *J* = 8.3 Hz, 1H), 7.30-7.27 (m, 1H), 7.23-7.18 (m, 5H), 7.15 (s, 2H), 2.55 (s, 3H), 2.54 (s, 3H), 2.50 (s, 3H), 1.98 (s, 6H), 1.87 (s, 6H), 1.85 (s, 6H).

Fused triphenylene-porphyrin PorTrip

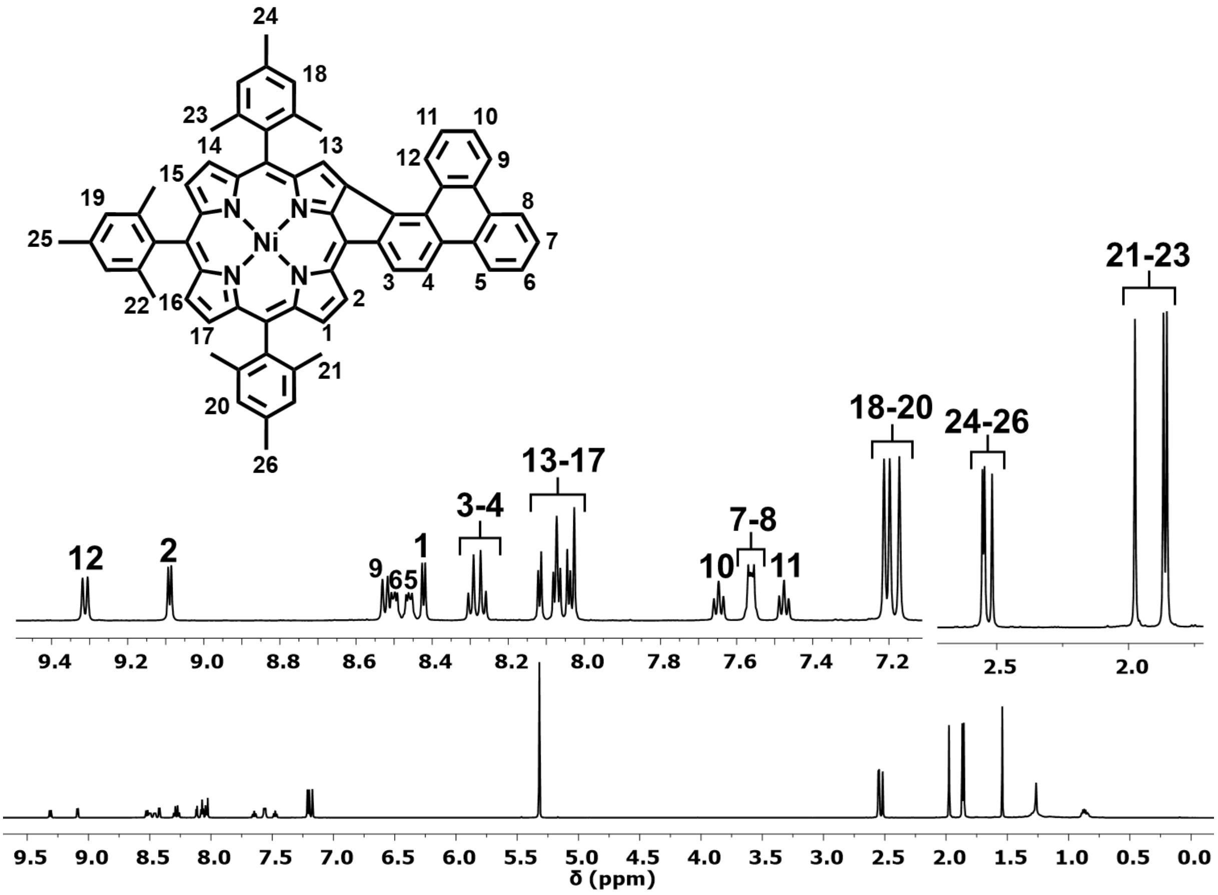

Figure S33. ^{1}H NMR of **PorTrip** with labeled protons.

^{1}H NMR (601 MHz, CD_2Cl_2, rt): δ [ppm]: 9.31 (d, *J* = 8.0 Hz, 1H), 9.09 (d, *J* = 4.9 Hz, 1H), 8.55-8.44 (m, 3H), 8.42 (d, *J* = 4.9 Hz, 1H), 8.32-8.24 (m, 2H), 8.15-7.99 (m, 5H), 7.68-7.63 (m, 1H), 7.58-7.56 (m, 2H), 7.49-7.46 (m, 1H), 7.21-7.17 (m, 6H), 2.55 (m, 6H), 2.52 (s, 3H), 1.98 (s, 6H), 1.87-1.86 (m, 12H).

Fused dibenzotetracene-porphyrin PorDbtc

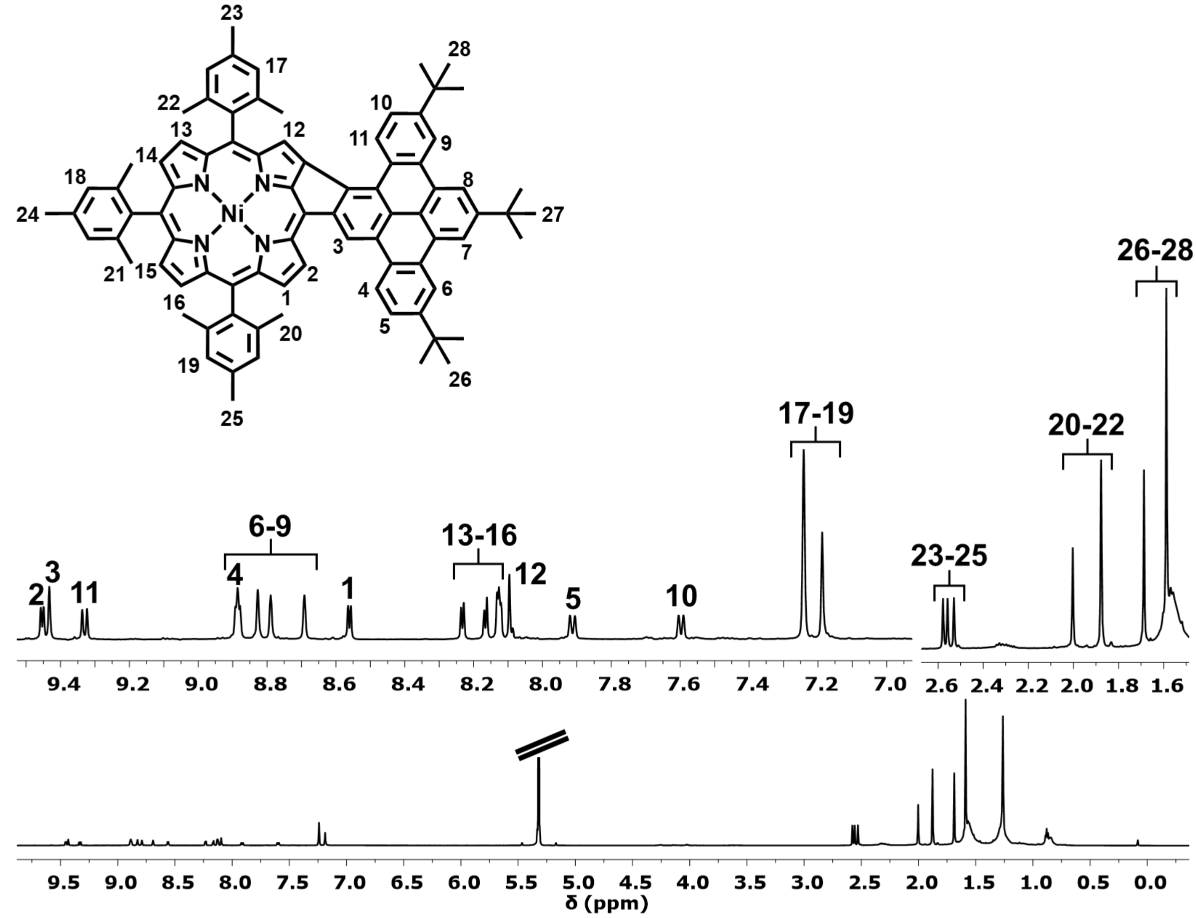

Figure S34. ^{1}H NMR of **PorDbtc** with labeled protons.

^{1}H NMR (601 MHz, CD_2Cl_2, rt): δ [ppm]: 9.47-9.41 (m, 2H), 9.33 (d, *J* = 8.4 Hz, 1H), 8.89-8.88 (m, 2H), 8.83 (s, 1H), 8.79 (s, 1H), 8.69 (s, 1H), 8.58-8.56 (m, 1H), 8.24-8.23 (m, 1H), 8.18-8.08 (m, 4H), 7.92-7.90 (m, 1H), 7.61-7.59 (m, 1H), 7.24-7.19 m, 6H), 2.58 (s, 3H), 2.56 (s, 3H), 2.53 (s, 3H), 2.00 (s, 6H), 1.88 (s, 6H), 1.69 (s, 6H), 1.59 (s, 18H), 1.26 (s, 9H).

4 DFT Calculations

Geometries were relaxed using density-functional theory (DFT). The calculations were carried out with the plane-wave code PWScf of the Quantum Espresso software package,[2] utilizing the gradient-corrected Perdew-Burke-Ernzerhof (PBE) exchange-correlation functional,[3] Grimme D3 dispersion correction with Becke-Johnson damping,[4,5] Vanderbilt ultrasoft pseudopotentials,[6] and a plane-wave basis set with a kinetic energy cutoff of 30 Ry. Structures were assumed to be relaxed when a force convergence threshold of 5 meV/Å was reached. The Nudged Elastic Band (NEB) calculations were done using the same settings.

Electronic properties were determined with the ORCA code,[7] using the B3LYP hybrid exchange-correlation functional,[8,9] the triple-zeta def2-TZVPP basis set,[10] and the RIJCOSX approximation with def2/J auxiliary basis functions.[11] Time-dependent density functional theory (TD-DFT) was used for the calculation of absorption spectra, utilizing the same settings but changing to the CAM-B3LYP long-range corrected hybrid exchange-correlation functional.[12] The lowest 150 vertical transitions were included in the TD-DFT calculations. In Figures S39-S41, the transitions were shifted by 88 nm to higher wavelengths to facilitate comparison. Solvation effects in DCM were taken into account by employing the implicit conductor-like continuum polarization model (C-PCM).[7]

**PorNaph**	**PorTrip**	**PorDbtc**	
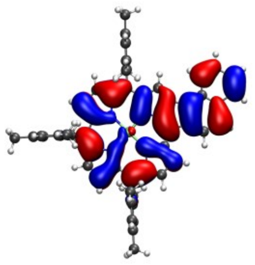	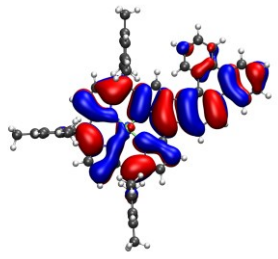	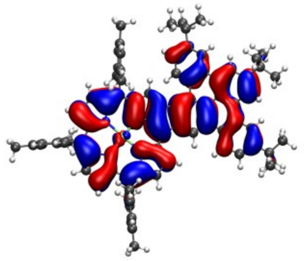	**HOMO**
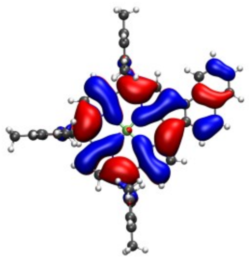	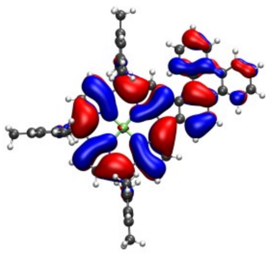	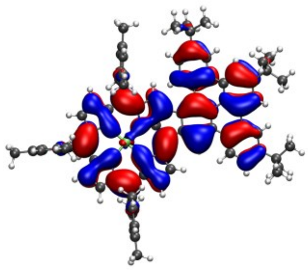	**HOMO-1**
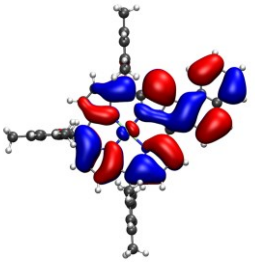	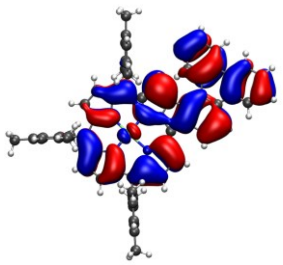	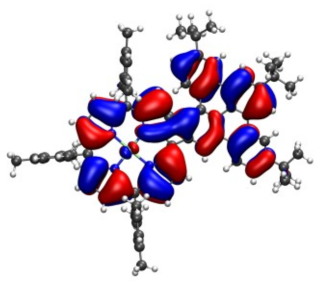	**HOMO-2**
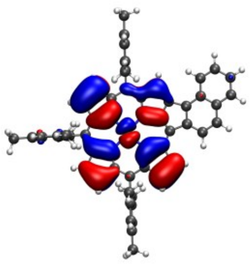	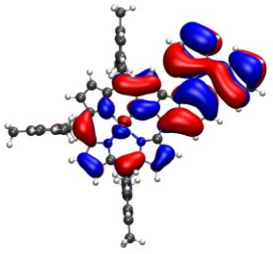	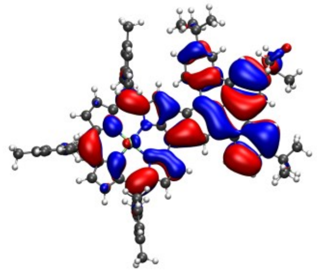	**HOMO-3**

Figure S35. Geometry optimized structures and orbitals of **PorNaph**, **PorTrip**, and **PorDbtc**.

PorNaph	PorTrip	PorDbtc	
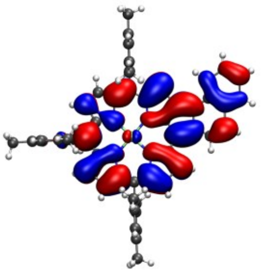	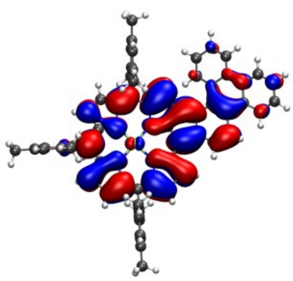	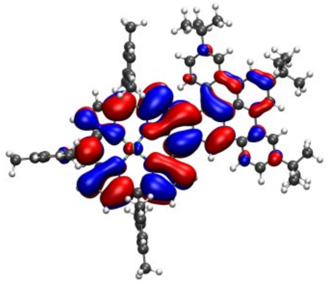	**LUMO**
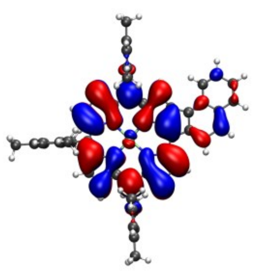	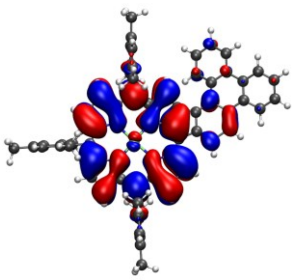	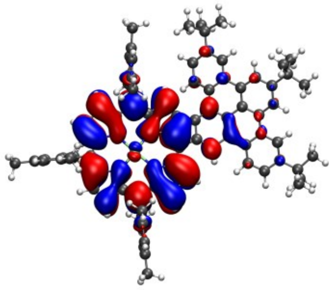	**LUMO+1**
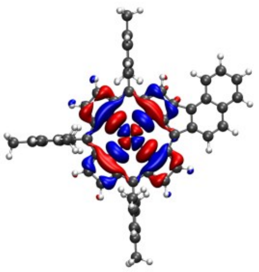	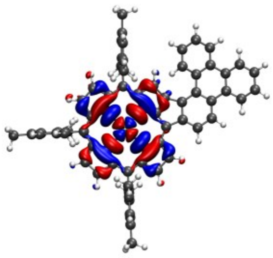	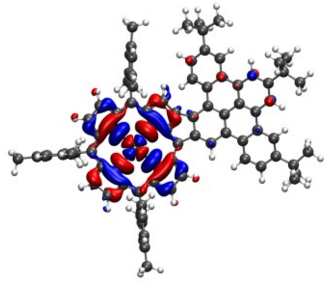	**LUMO+2**
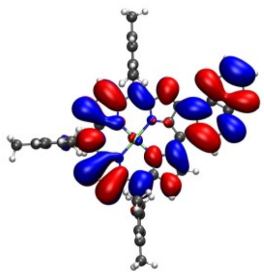	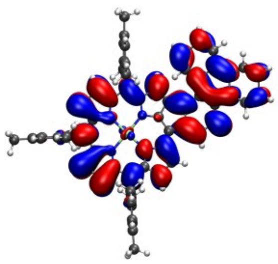	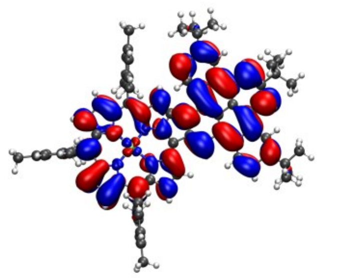	**LUMO+3**

Figure S36. Geometry optimized structures and orbitals of **PorNaph**, **PorTrip**, and **PorDbtc**.

Orbital	Por	Naph	Orbital	Por	Naph
HOMO	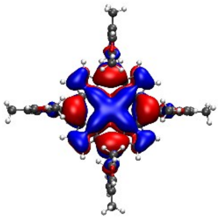	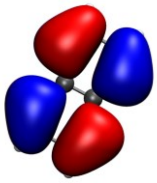	LUMO	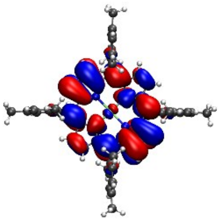	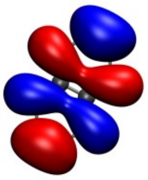
HOMO-1	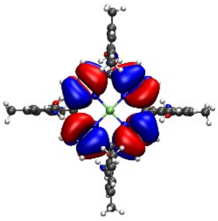	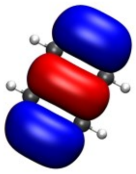	LUMO+1	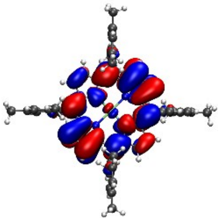	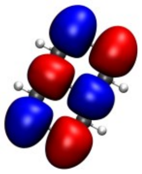
HOMO-2	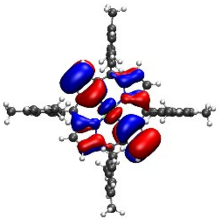	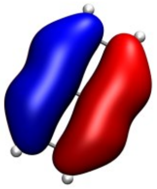	LUMO+2	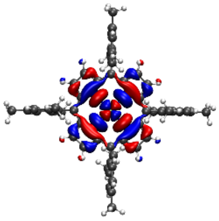	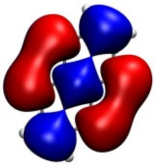
HOMO-3	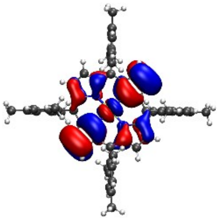	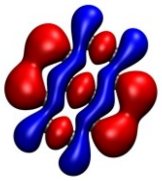	LUMO+3	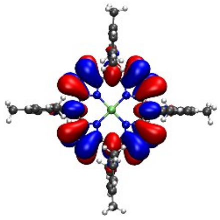	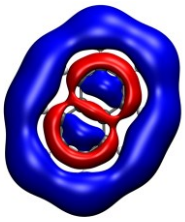

Figure S37. Geometry optimized structures and orbitals of **Por** and **Naph**.

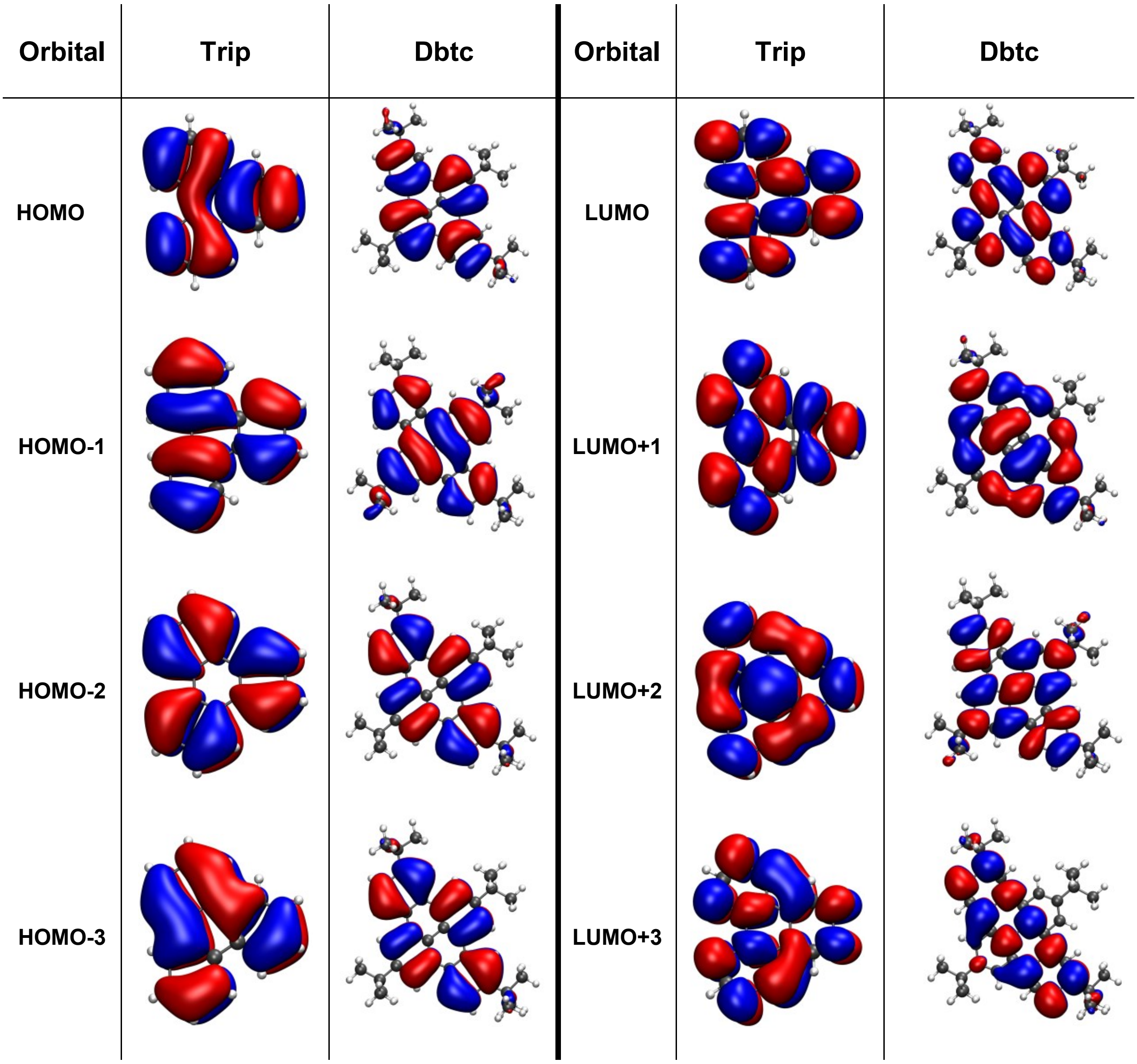

Figure S38. Geometry optimized structures and orbitals of **Trip** and **Dbtc**.

Table S1. Energy eigenvalues of selected orbitals of **PorNaph**, **PorTrip**, and **PorDbtc**.

	**PorNaph**	**PorTrip**	**PorDbtc**
Orbital	Energy (eV)	Energy (eV)	Energy (eV)
HOMO-3	-6.378	-6.248	-6.018
HOMO-2	-5.922	-5.958	-5.747
HOMO-1	-5.597	-5.538	-5.379
HOMO	-5.111	-5.184	-5.133
LUMO	-2.895	-2.885	-2.830
LUMO+1	-2.484	-2.514	-2.516
LUMO+2	-1.706	-1.666	-1.630
LUMO+3	-1.560	-1.529	-1.620
GAP	2.216	2.298	2.302

Table S2. Energy eigenvalues of selected orbitals of **Por**, **Naph**, **Trip**, and **Dbtc**.

	**Por**	**Naph**	**Trip**	**Dbtc**
Orbital	Energy (eV)	Energy (eV)	Energy (eV)	Energy (eV)
HOMO-3	-6.276	-8.996	-7.842	-6.613
HOMO-2	-6.275	-7.939	-6.626	-6.231
HOMO-1	-5.477	-6.825	-6.120	-5.817
HOMO	-5.413	-6.052	-6.120	-5.539
LUMO	-2.438	-1.335	-1.303	-1.536
LUMO+1	-2.437	-0.574	-1.302	-1.191
LUMO+2	-1.516	0.356	-1.179	-1.056
LUMO+3	-0.940	1.593	0.403	-0.540
GAP	2.974	4.717	4.817	4.003

Table S3. TD-DFT excitation energies and oscillator strengths of **PorNaph**.

Excited state	Energy (nm)	Energy (eV)	Oscillator strength *f*	Excited state	Energy (nm)	Energy (eV)	Oscillator strength *f*
1	657.1	1.887	0.028	16	319.4	3.882	0.837
2	596.7	2.078	0.000	17	315.4	3.931	0.000
3	573.3	2.163	0.000	18	311.0	3.986	0.000
4	571.2	2.171	0.000	19	309.7	4.004	0.059
5	546.1	2.270	0.009	20	304.8	4.068	0.023
6	473.4	2.619	0.001	21	300.9	4.120	0.000
7	437.2	2.836	0.024	22	296.8	4.177	0.021
8	397.9	3.116	2.471	23	296.3	4.185	0.000
9	381.0	3.254	0.001	24	290.6	4.266	0.018
10	364.3	3.403	0.616	25	290.1	4.273	0.013
11	355.3	3.490	0.460	26	289.4	4.285	0.003
12	342.9	3.615	0.288	27	287.7	4.309	0.000
13	340.5	3.641	0.000	28	283.2	4.378	0.005
14	331.5	3.740	0.924	29	282.3	4.392	0.000
15	320.6	3.868	0.013	30	280.9	4.414	0.000

Table S4. Orbital transitions and their relative contributions to optically active excitations with large oscillator strengths from the TD-DFT calculations of **PorNaph**. Only transitions with contributions exceeding 10% are listed.

State	Energy (eV)	Transition	Contribution (%)
1	1.887	HOMO → LUMO	86.6
5	2.270	HOMO-1 → LUMO	46.8
		HOMO → LUMO+1	48.0
7	2.836	HOMO-2 → LUMO	43.9
		HOMO-1 → LUMO+1	43.6
8	3.116	HOMO-1 → LUMO	39.4
		HOMO → LUMO+1	42.0
10	3.403	HOMO-9 → LUMO	10.9
		HOMO-2 → LUMO	29.5
		HOMO-1 → LUMO+1	22.7
11	3.490	HOMO-9 → LUMO	45.3
12	3.615	HOMO-10 → LUMO	23.2
		HOMO-2 → LUMO+1	35.4
14	3.740	HOMO-10 → LUMO	25.7
		HOMO-9 → LUMO+1	12.0
		HOMO-2 → LUMO+1	13.5
16	3.882	HOMO → LUMO+2	52.5

Table S5. TD-DFT excitation energies and oscillator strengths of **PorTrip**.

Excited state	Energy (nm)	Energy (eV)	Oscillator strength *f*	Excited state	Energy (nm)	Energy (eV)	Oscillator strength *f*
1	628.5	1.973	0.046	16	321.4	3.858	0.002
2	587.3	2.111	0.000	17	314.5	3.942	0.000
3	564.5	2.196	0.000	18	310.8	3.990	0.007
4	562.3	2.205	0.000	19	309.9	4.001	0.112
5	546.9	2.267	0.021	20	308.9	4.014	0.123
6	470.8	2.634	0.001	21	303.7	4.082	0.029
7	432.8	2.864	0.067	22	300.8	4.122	0.001
8	404.2	3.067	2.965	23	299.2	4.144	0.049
9	370.1	3.350	0.043	24	298.9	4.149	0.032
10	369.7	3.353	0.425	25	293.5	4.224	0.041
11	356.6	3.477	0.202	26	289.7	4.279	0.001
12	346.1	3.583	0.353	27	289.1	4.288	0.002
13	338.0	3.669	0.001	28	289.0	4.291	0.019
14	332.6	3.728	0.509	29	287.8	4.308	0.207
15	330.0	3.757	0.637	30	285.7	4.339	0.189

Table S6. Orbital transitions and their relative contributions to optically active excitations with large oscillator strengths from the TD-DFT calculations of **PorTrip**. Only transitions with contributions exceeding 10% are listed.

State	Energy (eV)	Transition	Contribution (%)
1	1.973	HOMO → LUMO	83.3
5	2.267	HOMO-1 → LUMO	50.8
		HOMO → LUMO+1	44.2
7	2.864	HOMO-2 → LUMO	35.9
		HOMO-1 → LUMO+1	42.8
8	3.067	HOMO-1 → LUMO	36.0
		HOMO → LUMO+1	40.7
9	3.350	HOMO → LUMO+4	47.6
		HOMO → LUMO+5	26.7
10	3.353	HOMO-3 → LUMO	11.4
		HOMO-2 → LUMO	25.3
		HOMO-1 → LUMO+1	15.5
11	3.477	HOMO-11 → LUMO	56.2
14	3.728	HOMO-12 → LUMO	24.8
		HOMO-11 → LUMO+1	15.6
15	3.757	HOMO-3 → LUMO	30.8
		HOMO-2 → LUMO+1	11.2

Table S7. TD-DFT excitation energies and oscillator strengths of **PorDbtc**.

Excited state	Energy (nm)	Energy (eV)	Oscillator strength *f*	Excited state	Energy (nm)	Energy (eV)	Oscillator strength *f*
1	617.5	2.008	0.022	16	320.1	3.874	0.002
2	582.2	2.129	0.000	17	318.4	3.894	0.092
3	560.1	2.214	0.000	18	315.9	3.924	0.013
4	557.7	2.223	0.000	19	312.5	3.967	0.024
5	550.5	2.252	0.033	20	311.5	3.980	0.341
6	469.2	2.642	0.001	21	311.3	3.983	0.051
7	448.8	2.763	0.405	22	307.9	4.027	0.001
8	415.2	2.986	2.789	23	301.5	4.112	0.077
9	385.6	3.215	0.246	24	300.5	4.125	0.003
10	364.8	3.399	0.005	25	299.4	4.141	0.314
11	361.3	3.431	0.622	26	298.9	4.149	0.004
12	354.6	3.496	0.376	27	294.4	4.211	0.332
13	341.2	3.634	0.166	28	290.1	4.273	0.002
14	338.0	3.668	0.572	29	288.9	4.292	0.049
15	337.8	3.671	0.243	30	288.6	4.296	0.080

Table S8. Orbital transitions and their relative contributions to optically active excitations with large oscillator strengths from the TD-DFT calculations of **PorDbtc**. Only transitions with contributions exceeding 10% are listed.

State	Energy (eV)	Transition	Contribution (%)
1	2.008	HOMO → LUMO	81.7
5	2.252	HOMO-1 → LUMO	48.2
		HOMO → LUMO+1	40.0
7	2.763	HOMO-2 → LUMO	33.0
		HOMO-1 → LUMO	10.9
		HOMO-1 → LUMO+1	27.1
8	2.986	HOMO-1 → LUMO	29.5
		HOMO-1 → LUMO+1	11.2
		HOMO → LUMO+1	40.1
9	3.215	HOMO-2 → LUMO	26.0
		HOMO-2 → LUMO+1	14.6
11	3.431	HOMO-13 → LUMO	16.6
		HOMO-2 → LUMO+1	17.8
		HOMO-1 → LUMO+1	18.6
14	3.668	HOMO-3 → LUMO	15.5
		HOMO-2 → LUMO+1	18.7
		HOMO-1 → LUMO+4	12.9

Calculated Absorption Spectra

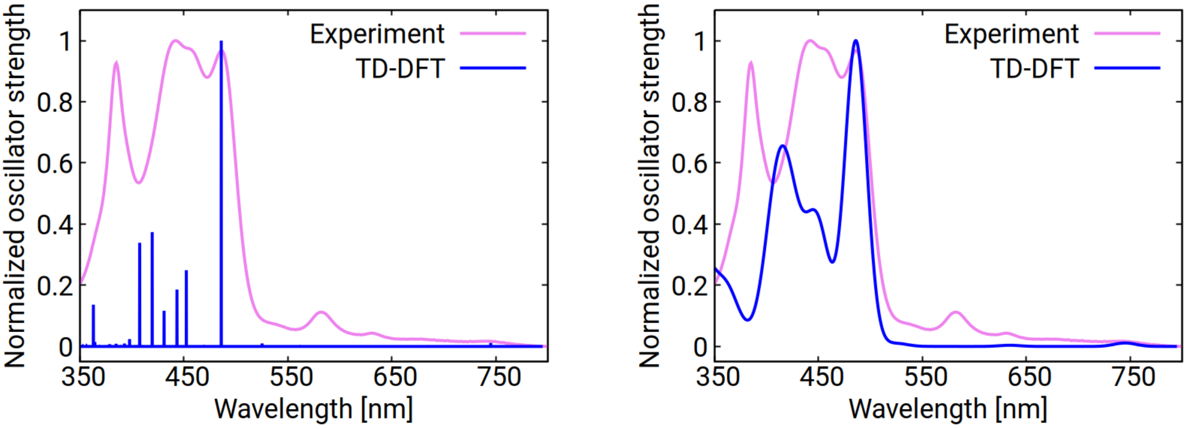

Figure S39. *Left*: Experimental UV/Vis spectrum overlaid with the TD-DFT calculated transitions for **PorNaph** (line spectrum). *Right:* The calculated transitions are homogeneously broadened by a Gaussian function with a width of 12 nm.

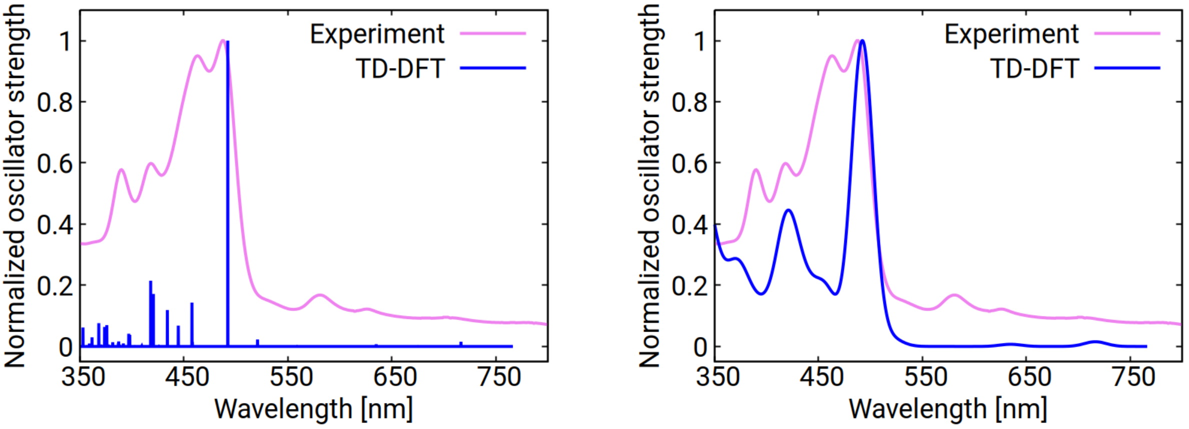

Figure S40. *Left*: Experimental UV/Vis spectrum overlayed with the TD-DFT calculated transitions for **PorTrip** (line spectrum). *Right:* The calculated transitions are homogeneously broadened by a Gaussian function with a width of 12 nm.

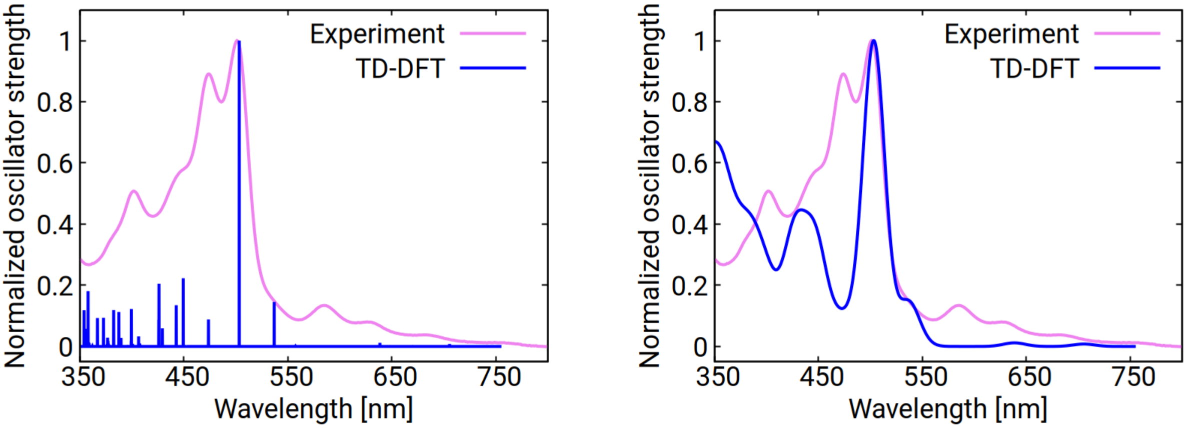

Figure S41. *Left*: Experimental UV/Vis spectrum overlayed with the TD-DFT calculated transitions for **PorDbtc** (line spectrum). *Right:* The calculated transitions are homogeneously broadened by a Gaussian function with a width of 12 nm.

Cartesian Coordinates of Calculated Structures

Fused naphthalene-porphyrin PorNaph

Cartesian Coordinates (Å)

	X	Y	Z
C	24.49980	19.96976	15.24833
N	23.11607	19.75335	15.27595
N	20.32056	19.64119	15.21882
C	18.31858	18.45134	15.15341
C	18.93592	19.75095	15.12598
C	18.20959	20.92915	15.04901
C	24.20672	22.34327	15.16015
Ni	21.61159	21.08254	15.20974
C	25.21258	18.72618	15.34263
H	17.26752	20.02282	17.63741
C	26.42840	21.78250	15.13321
C	22.98707	18.37775	15.38195
C	20.55793	18.27797	15.31192
C	19.32147	17.53949	15.27714
C	21.80001	17.65861	15.40772
C	24.27864	17.74024	15.43231
H	17.24758	18.28370	15.09127
H	26.29210	18.62405	15.34946
C	25.04598	21.23902	15.16897
H	19.25595	16.45737	15.33669
H	24.42661	16.66813	15.52097
C	24.94928	23.57225	15.14972
C	24.00637	24.56873	15.18613
C	22.71441	23.90231	15.20578
C	20.30298	23.80287	15.17921
C	19.00879	24.41934	15.12777
C	18.08947	23.41284	15.05668
C	21.48922	24.53978	15.21414
C	21.43564	26.03058	15.23797
C	21.42193	26.75245	14.02804
C	21.37681	28.15068	14.07665
C	21.34693	28.84699	15.28921
C	21.35803	28.10632	16.47631
C	21.40323	26.70807	16.47355
H	18.84518	25.49285	15.13823
H	24.11736	25.64938	15.19899
C	21.32944	30.35248	15.31856
H	22.34980	30.75608	15.42300
H	20.90563	30.76695	14.39344
H	20.74200	30.73038	16.16737
C	21.44972	26.03592	12.70396
H	20.58131	25.36820	12.59330
H	21.44343	26.75069	11.87076
H	22.34558	25.40327	12.60986
C	21.41322	25.94126	17.76940
H	20.55287	25.25754	17.83527
H	22.31606	25.31759	17.85773
H	21.37954	26.62263	18.62945
H	21.32835	28.63048	17.43580
H	21.36199	28.71009	13.13699
N	22.86575	22.51289	15.19314
N	20.18836	22.41535	15.15328
C	18.82248	22.17890	15.07810
H	17.00733	23.47986	14.99941
C	16.72058	20.85866	14.95676
C	14.55070	20.77536	16.02165
C	15.94403	20.83848	16.13284
C	16.59987	20.88537	17.48748
H	17.22190	21.78664	17.59982
H	13.94939	20.76364	16.93521
H	15.84841	20.88222	18.28777
C	16.10004	20.81887	13.69266
C	14.70337	20.75514	13.62662
C	13.91034	20.72923	14.77845
C	12.41090	20.62654	14.68482
H	12.03875	21.03949	13.73698
H	12.08474	19.57486	14.73562
H	11.91951	21.16001	15.51074
C	16.92368	20.84796	12.43242
H	17.63998	20.01263	12.40438
H	16.28363	20.78419	11.54280
H	14.22302	20.72679	12.64445
H	17.51798	21.77243	12.36503
C	21.96161	16.03054	12.98616
H	22.84374	16.67925	12.87027
H	21.98492	15.27324	12.19166
H	21.07996	16.67062	12.82926
C	21.87870	14.15977	16.86056
C	21.98388	13.99136	14.46882
C	21.96073	13.35903	15.71721
C	22.04613	11.85934	15.82374
H	21.47388	11.36683	15.02443
H	23.08993	11.51699	15.73408
H	21.66456	11.50350	16.79057
H	22.03971	13.38394	13.56087
H	21.85159	13.68558	17.84570
C	21.93410	15.38394	14.34583
C	21.85669	16.16940	15.51427
C	21.82695	15.55675	16.78215
H	22.62224	17.04342	18.13749
C	21.74328	16.38808	18.03491
H	20.86198	17.04729	18.02065
H	21.68402	15.75025	18.92651
C	26.36173	23.20130	15.12463
C	27.67886	21.14029	15.10178
C	28.83939	21.89775	15.07002
C	28.80452	23.31462	15.06603
C	27.53163	23.99184	15.09219
C	27.50969	25.41259	15.08381
C	29.99082	24.09448	15.03430
C	29.93506	25.47193	15.02808
C	28.68300	26.13516	15.05277
H	27.75073	20.05342	15.09740
H	29.81182	21.40103	15.04575
H	30.95434	23.57968	15.01456
H	30.85576	26.05713	15.00365
H	28.65071	27.22605	15.04682
H	26.54751	25.92599	15.10219

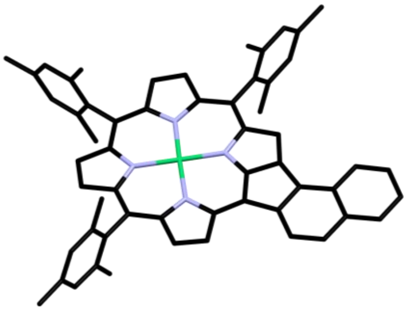

Fused triphenylene-porphyrin PorTrip

Cartesian Coordinates (Å)

	X	Y	Z
C	21.90101	19.65398	15.77571
N	20.52879	19.41033	15.90885
N	17.74030	19.23893	15.96980
C	15.76677	18.00585	15.90582
C	16.35520	19.31897	15.86913
C	15.60447	20.48441	15.81566
C	21.55184	22.01929	15.64014
Ni	18.99984	20.70276	15.92436
C	22.64588	18.43133	15.89025
H	14.64427	19.44625	18.37313
C	23.77108	21.50053	15.48493
C	20.43634	18.03962	16.09038
C	18.00817	17.88389	16.09026
C	16.78785	17.11864	16.06159
C	19.26611	17.29737	16.18920
C	21.74286	17.43307	16.09617
H	14.70039	17.81325	15.83657
H	23.72509	18.34801	15.83015
C	22.40902	20.93187	15.59805
H	16.74441	16.03670	16.14222
H	21.92224	16.37060	16.23079
C	22.26698	23.26513	15.59284
C	21.31296	24.22802	15.82848
C	20.04711	23.53277	15.95796
C	17.64254	23.38877	16.09286
C	16.33509	23.98029	16.10789
C	15.43486	22.96323	15.97278
C	18.81625	24.14469	16.10994
C	18.74901	25.63099	16.22011
C	18.69738	26.41822	15.05227
C	18.66084	27.81179	15.17846
C	18.67519	28.44090	16.42749
C	18.71880	27.63626	17.57135
C	18.75761	26.24017	17.49092
H	16.15116	25.04718	16.19369
H	21.40819	25.30580	15.91422
C	18.67184	29.94248	16.53961
H	19.69701	30.32976	16.65766
H	18.24421	30.41110	15.64253
H	18.09518	30.27855	17.41310
C	18.67738	25.77641	13.69006
H	17.80960	25.10844	13.57592
H	18.63366	26.53629	12.89893
H	19.57241	25.15704	13.52507
C	18.80958	25.40367	18.74188
H	17.93827	24.73423	18.81137
H	19.70171	24.75900	18.75434
H	18.82971	26.03759	19.63788
H	18.72484	28.10732	18.55838
H	18.61770	28.42181	14.27165
N	20.22107	22.15353	15.84335
N	17.55238	22.00414	15.97729
C	16.19145	21.74432	15.90283
H	14.35107	23.01291	15.93126
C	14.11786	20.38738	15.71342
C	11.94295	20.22552	16.75876
C	13.33435	20.30833	16.88244
C	13.97934	20.31429	18.24312
H	14.60171	21.21058	18.38748
H	11.33586	20.16954	17.66681
H	13.22121	20.28833	19.03671
C	13.50617	20.38454	14.44431
C	12.11143	20.29945	14.36588
C	11.31152	20.21643	15.51028
C	9.81459	20.09416	15.40191
H	9.44006	20.55588	14.47774
H	9.50621	19.03599	15.38850
H	9.31018	20.56977	16.25479
C	14.33731	20.47349	13.19179
H	15.04658	19.63431	13.12343
H	13.70167	20.46155	12.29678
H	11.63804	20.30114	13.37995
H	14.94011	21.39456	13.17608
C	19.42658	15.58174	13.82775
H	20.29288	16.24327	13.67179
H	19.45262	14.79505	13.06251
H	18.52914	16.19714	13.66184
C	19.44709	13.85928	17.77150
C	19.51605	13.60177	15.38641
C	19.52692	13.01719	16.65762
C	19.64813	11.52505	16.82075
H	19.15233	10.98997	15.99849
H	20.70550	11.21365	16.82200
H	19.20613	11.18764	17.76851
H	19.56975	12.96119	14.50148
H	19.44796	13.42285	18.77437
C	19.43517	14.98763	15.21140
C	19.35909	15.81497	16.35041
C	19.36293	15.25049	17.64109
H	20.07488	16.88153	18.87226
C	19.27344	16.12736	18.86202
H	18.32220	16.68167	18.88665
H	19.34762	15.53097	19.78076
C	23.69307	22.93128	15.44757
C	25.01539	20.87494	15.46514
C	26.16845	21.65127	15.50674
C	26.12893	23.05757	15.50375
C	24.86408	23.71801	15.36262
C	27.34081	23.85048	15.71280
C	27.26827	25.27141	15.73986
H	25.10477	19.78993	15.45991
H	27.12681	21.13764	15.55766
C	26.02189	25.93451	15.36313
C	24.85254	25.15958	15.12577
C	23.72312	25.80106	14.57587
C	25.96124	27.32837	15.14974
C	24.81256	27.94618	14.68012
C	23.69028	27.16974	14.36245
H	26.84541	27.93979	15.32536
H	24.80042	29.02602	14.52363
H	22.79934	27.63209	13.93382
H	22.87113	25.19766	14.27587
C	28.42515	26.00529	16.07858
C	28.58953	23.24022	15.96912
C	29.71858	23.98352	16.27125
C	29.63346	25.38103	16.34162
H	28.67642	22.15494	15.95111
H	30.66510	23.47808	16.46927
H	30.51025	25.97580	16.60228
H	28.37528	27.09174	16.14020

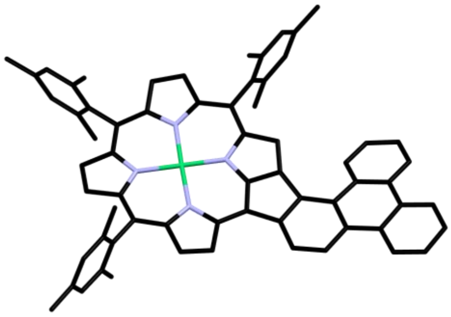

Fused dibenzotetracene-porphyrin PorDbtc

Cartesian Coordinates (Å)

	X	Y	Z				
C	21.95887	19.74076	15.62679	C	19.50627	15.50014	13.95810
N	20.60159	19.46470	15.83282	H	20.32665	16.20018	13.73712
N	17.82380	19.22017	15.99026	H	19.54920	14.67822	13.23154
C	15.88652	17.93212	15.98846	H	18.56974	16.05574	13.79665
C	16.43662	19.25954	15.90288	C	19.75828	13.98112	17.97658
C	15.65314	20.40426	15.83446	C	19.72616	13.60561	15.60688
C	21.53926	22.09333	15.48737	C	19.81227	13.08616	16.90319
Ni	19.03979	20.71406	15.88638	C	19.98532	11.60801	17.13388
C	22.74068	18.54207	15.74370	H	19.47627	11.01796	16.35868
H	14.73500	19.37297	18.40083	H	21.05112	11.32787	17.10949
C	23.76541	21.63824	15.23012	H	19.58996	11.30516	18.11342
C	20.55492	18.09950	16.06631	H	19.75789	12.92273	14.75310
C	18.13251	17.87801	16.15311	H	19.81657	13.59600	18.99860
C	16.93378	17.07965	16.16822	C	19.59732	14.97843	15.36773
C	19.40941	17.33105	16.24034	C	19.55018	15.86052	16.46664
C	21.87648	17.52835	16.03026	C	19.62840	15.36130	17.78201
H	14.82543	17.70734	15.93627	H	20.38103	17.03854	18.92447
H	23.81816	18.48524	15.63631	C	19.56992	16.29533	18.96178
C	22.42406	21.02961	15.40528	H	18.62692	16.86334	18.97490
H	16.92135	16.00052	16.28888	H	19.65247	15.74183	19.90634
H	22.09183	16.47664	16.19382	C	23.63597	23.07190	15.20966
C	22.21559	23.36074	15.41912	C	25.01334	21.05284	15.14805
C	21.24804	24.29547	15.70712	C	26.18399	21.84394	15.13587
C	20.01047	23.56540	15.88584	C	26.06665	23.26144	15.17337
C	17.61596	23.36274	16.09604	C	24.77467	23.88445	15.10130
C	16.29314	23.91867	16.13508	C	27.23888	24.07454	15.36942
C	15.41863	22.87916	15.99911	C	27.12392	25.49110	15.51937
C	18.77066	24.14644	16.08801	H	25.10340	19.97016	15.14874
C	18.69812	25.63246	16.20898	C	25.84143	26.13520	15.23483
C	18.61555	26.42683	15.04698	C	24.71075	25.33197	14.94687
C	18.64354	27.82014	15.17707	C	23.55536	25.97150	14.45375
C	18.75219	28.44392	16.42463	C	25.70851	27.54018	15.16953
C	18.81070	27.63378	17.56346	C	24.53231	28.17173	14.77985
C	18.78785	26.23697	17.47868	C	23.46185	27.34755	14.37463
H	16.08288	24.97983	16.23421	H	26.57133	28.15509	15.41429
H	21.31929	25.37410	15.79875	C	24.37350	29.69465	14.74296
C	18.83828	29.94325	16.53565	H	22.54227	27.79074	13.98768
H	19.88833	30.27862	16.50560	H	22.72773	25.36300	14.09895
H	18.31293	30.43900	15.70739	C	28.25514	26.22918	15.87910
H	18.40866	30.30218	17.48138	C	28.52146	23.46899	15.50343
C	18.51134	25.79188	13.68546	C	29.62250	24.26853	15.85896
H	17.63033	25.13557	13.61642	C	29.51238	25.64110	16.06696
H	18.43528	26.55585	12.90065	H	30.58957	23.79319	16.00033
H	19.38772	25.16135	13.46948	H	28.15778	27.30426	16.02429
C	18.87561	25.39476	18.72374	C	27.50452	21.23394	15.10731
H	17.99969	24.73569	18.82403	C	28.66388	22.03399	15.28242
H	19.75931	24.73876	18.69902	C	27.68662	19.84815	14.89351
H	18.93924	26.02412	19.62098	C	28.94207	19.27470	14.85664
H	18.88577	28.10033	18.54971	H	29.02473	18.20063	14.68132
H	18.58208	28.43457	14.27425	H	26.82150	19.20612	14.73105
N	20.21649	22.19180	15.75279	C	29.93511	21.41714	15.22787
N	17.56024	21.97690	15.97148	C	30.10752	20.05527	15.02312
C	16.20614	21.68023	15.91140	H	30.81806	22.04266	15.33335
H	14.33327	22.89976	15.97126	C	23.17233	30.09419	15.62710
C	14.16908	20.26526	15.73784	H	22.24094	29.62236	15.28333
C	12.00040	20.05323	16.78811	H	23.33735	29.79052	16.67124
C	13.38929	20.17542	16.90873	H	23.02966	31.18562	15.60251
C	14.03540	20.21277	18.26844	C	25.61920	30.42635	15.26131
H	14.62207	21.13363	18.40797	H	26.50277	30.22004	14.63909
H	11.39629	19.98881	17.69750	H	25.44473	31.51208	15.23944
H	13.28048	20.16016	19.06383	H	25.85235	30.14498	16.29905
C	13.55497	20.23420	14.46997	C	24.10965	30.14674	13.29153
C	12.16303	20.10965	14.39483	H	23.97446	31.23863	13.25164
C	11.36725	20.01529	15.54117	H	24.95439	29.87942	12.64007
C	9.87399	19.85107	15.43604	H	23.20455	29.67885	12.87974
H	9.48426	20.30376	14.51364	C	30.70322	26.51466	16.48051
H	9.59505	18.78480	15.42094	C	30.38927	27.19897	17.82719
H	9.35808	20.31068	16.29079	H	31.22749	27.84511	18.13041
C	14.38047	20.33955	13.21490	H	29.48707	27.82344	17.76432
H	15.12934	19.53473	13.15963	C	31.99612	25.70245	16.64185
H	13.74608	20.28295	12.32066	H	32.29272	25.21645	15.70041
H	11.68805	20.08938	13.40991	H	32.81518	26.37177	16.94323
H	14.93866	21.28798	13.18324	H	31.89696	24.92721	17.41611

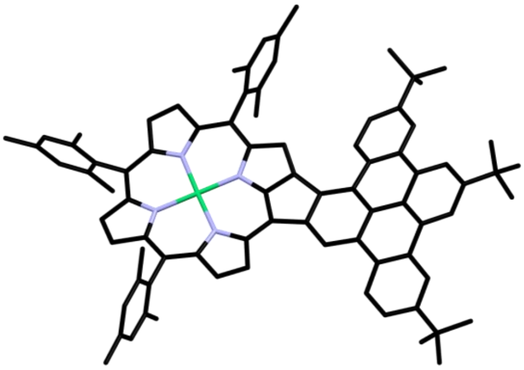

C	30.94046	27.59275	15.40233
H	31.78983	28.23371	15.68465
H	31.16516	27.12976	14.43034
H	30.05981	28.23740	15.27272
H	30.22655	26.45103	18.61693
C	31.48553	19.38934	14.96162
C	31.58044	18.32253	16.07254
H	30.80491	17.55171	15.96233
H	32.56088	17.82316	16.03702
C	31.66293	18.71168	13.58665
H	31.59943	19.45180	12.77567
H	32.64613	18.21984	13.52948
H	30.89310	17.94818	13.40764
C	32.63125	20.39233	15.15611
H	33.59437	19.86333	15.10773
H	32.63529	21.16388	14.37190
H	32.57451	20.89227	16.13450
H	31.46207	18.78100	17.06521

5 References

[1] R. M. Al-Zoubi, H. A. Futouh, R. McDonald, *Aust. J. Chem.*, **2013**, *66*, 1570.

[2] P. Giannozzi, *et al.*, *J. Condens. Matter Phys.,* **2009**, *21*, 395502.

[3] J. P. Perdew, K. Burke, M. Ernzerhof, *Phys. Rev. Lett.,* **1996**, *77*, 3865-3868.

[4] S. Grimme, S. Ehrlich, L. Goerigk, *J. Comput. Chem.,* **2011**, *32*, 1456-1465.

[5] S. Grimme, J. Antony, S. Ehrlich, H. Krieg, *J. Chem. Phys.,* **2010**, *132*, 154104.

[6] D. Vanderbilt, *Phys. Rev. B,* **1990**, *41*, 7892-7895.

[7] F. Neese, *WIREs Comput. Mol. Sci.,* **2012**, *2*, 73-78.

[8] A. D. Becke, *J. Chem. Phys.,* **1993**, *98*, 1372-1377.

[9] C. Lee, W. Yang, R. G. Parr, *Phys. Rev. B,* **1988**, *37*, 785-789.

[10] F. Weigend, R. Ahlrichs, *Phys. Chem. Chem. Phys.,* **2005**, *7*, 3297.

[11] F. Neese, F. Wennmohs, A. Hansen, U. Becker, *Chem. Phys.,* **2009**, *356*, 98-109.

[12] T. Yanai, D. P. Tew, N. C. Handy, *Chem. Phys. Lett.,* **2004**, *393*, 51-58.
